# Functional characterisation of brassinosteroid receptor *MtBRI1* in *Medicago truncatula*

**DOI:** 10.1038/s41598-017-09297-9

**Published:** 2017-08-24

**Authors:** Xiaofei Cheng, Xiaoping Gou, Hongju Yin, Kirankumar S. Mysore, Jia Li, Jiangqi Wen

**Affiliations:** 1 Noble Research Institute, 2510 Sam Noble Parkway, Ardmore Oklahoma, 73401 USA; 2 Ministry of Education Key Laboratory of Cell Activities and Stress Adaptations, School of Life Sciences, Lanzhou University, Lanzhou Gansu Province, 730000 China; 3 College of Pastoral Agriculture Science and Technology, Lanzhou University, Lanzhou Gansu Province, 730000 China

## Abstract

Brassinosteroids are phytohormones involved in plant development and physiological processes. Brassinosteroids Insensitive 1 (BRI1) is required for BR perception and initiation of subsequent signal transduction in Arabidopsis. In this study, the orthologue of *BRI1* in the model legume species *Medicago truncatula*, *MtBRI1*, was identified and characterised. Three allelic *Tnt1* insertion mutants, *mtbri1-1*, *mtbri1-2*, and *mtbri1-3*, were obtained from the *M. truncatula Tnt1* insertion population. *mtbri1* mutants displayed characteristic *bri1* mutant phenotypes: extreme dwarfness, dark green curled leaves, short primary roots, less lateral roots, and insensitive to exogenous brassinolide (BL). Moreover, *mtbri1* mutants show decreased total nodule number and defects in nitrogen fixation. *MtBRI1* is able to complement an Arabidopsis *BRI1* mutant*, bri1-5*. Similar to the interaction of BRI1 and BAK1 in Arabidopsis, MtBRI1 interacts with MtSERK1 *in vivo*. Global gene expression profiling revealed that the expression of BR biosynthesis genes and *SAUR* genes are significantly altered in *mtbri1* mutants. MapMan analysis indicated that genes involved in signaling, hormone, cell wall, and biotic stress responses are over-represented in differentially expressed genes. Taken together, the results indicate that MtBRI1 is the BR receptor in *M. truncatula* and that BR signaling may play a conserved role in balancing plant growth and defenses.

## Introduction

Brassinosteroids (BRs) are phytohormones that play important roles in regulating plant growth, development, and stress responses. It is crucial to adjust and maintain appropriate levels of endogenous BRs for normal plant growth and development^1, 2^. A combination of genetics and analytical biochemistry approaches has helped elucidate the BR biosynthetic pathways in Arabidopsis^1^. BR biosynthesis is regulated by two major mechanisms. First, the level of endogenous BRs is regulated by modulating the transcriptional activity of the biosynthetic genes, such as *De-etiolated-2* (*DET2*)^3^, *Constitutive Photomorphogenesis and Dwarfism* (*CPD*)^4^, and *DWARF4* (*DWF4*)^5^. Second, BRs are inactivated, resulting in reduced levels of bioactive BRs^1^. The BR biosynthetic pathway is subject to feedback regulation at multiple points to ensure the homeostasis of endogenous BRs^6, 7^. When the endogenous BR level is high, accumulated Brassinazole-Resistant 1 (BZR1) and BRI1-EMS-Suppressor 1 (BES1)/BZR2 repress the expression of BR biosynthetic genes and activate genes for BR degradation as a feedback inhibition of BR function; when the endogenous BR level is low, the expression of BR biosynthetic genes is increased^7^. Besides the regulation of BR biosynthesis, inactivation of BRs is also pivotal in maintaining the hormonal homeostasis. *PhyB Activation-tagged Suppressor1* (*BAS1*) is the first yet the most important BR-inactivating gene identified^8^. Impairment or mutation of genes in BR biosynthesis leads to characteristic phenotypes including dwarfism, curled leaves, male sterility, and light-grown morphology in the dark^1, 2^.

In addition to biosynthesis, BR perception and subsequent signal transduction are also key components of BR functions in plant growth and development. Mutants of BR perception and signaling show similar phenotypes as BR biosynthesis mutants. While mutants impaired in BR biosynthesis can be rescued by application of exogenous BRs^4, 9–12^, BR signaling mutants are insensitive to BR treatment^13, 14^. With the help of genome sequencing and establishment of large scale mutant populations in model plant Arabidopsis, the BR signal transduction pathway and the downstream transcription regulatory network have been well elucidated by molecular, biochemical, genetics, and proteomics studies^15–20^. BR is perceived by the leucine rich repeat receptor protein kinase (LRR-RLK) BRI1 (Brassinosteroids Insensitive 1) located on the cell surface. Binding of BRs to BRI1 induces the association and transphosphorylation between BRI1 and BRI1-Associated Kinase 1 (BAK1), the co-receptor of BRs^21, 22^. The activated BRI1 phosphorylates BRI1 Kinase Inhibitor 1 (BKI1), resulting in the dissociation of BKI1 from BRI1 and thus from the plasma membrane^23^. Dissociated BKI1 phosphorylates Constitutive Differential Growth 1 (CDG1) and BR-Signaling Kinase 1 (BSK1) to promote their binding to the Phosphatase BRI1 Suppressor 1 (BSU1), which subsequently dephosphorylates BR-Insensitive 2 (BIN2) to inactivate its kinase activity^24^. When the BR level is low, BIN2 is phosphorylated and is in the active form. The phosphorylated BIN2 inactivates the transcription factors BZR1 and BES1/BZR2 to suppress the transcriptional activity. In contrast, when the BR level is high, BIN2 is dephosphorylated and inactivated by BSU1, such that BZR1 and BES1/BZR2 are dephosphorylated by PP2A and translocated into the nucleus to promote the BR-regulated downstream transcriptional network, thus altering the expression of genes and changing cellular events^14, 25, 26^. BZR1 and BES1/BZR2 share 88% overall amino acid identity and 97% identity in the DNA-binding domain. Both BZR1 and BES1/BZR2 bind to BR responsive elements (BRRE) and E-box that are present in promoters of BR responsive genes, directly or indirectly interacting with other transcription factors and functioning as transcription repressors or activators to coordinate the BR signaling, biosynthesis, and growth responses in the BR signaling pathway^27–30^.

BRI1 is a leucine-rich repeat (LRR) transmembrane receptor-like kinase and functions as the BR receptor. Binding of BR to the LRR domain activates BRI1 and initiates the BR-mediated signal transduction. Mutation of *BRI1* in Arabidopsis leads to dwarf plants with small curled dark green leaves, photomorphogenesis in dark, insensitiveness to exogenous BL treatment, accumulation of endogenous BRs, and feedback regulation of BR biosynthesis gene expression^7, 13, 31, 32^. *BRI1* orthologous genes have been isolated and characterised from several species, including dicots such as tomato (*Lycopersicon esculentum*) and pea (*Pisum sativum*), and monocots such as rice (*Oryza sativa*), barley (*Hordeum vulgare*) and *Brachypodium distachyon*. Mutations in *BRI1* orthologues cause similar pleiotropic phenotypes in pea, tomato, rice and barley^33–38^. The similarity in both gene sequences and mutant phenotypes indicates functional conservation among *BRI1* genes in different species.

To get insights of BR functions in the model legume species *M. truncatula*, we isolated *BRI1* orthologue, *MtBRI1*, and obtained three independent *Tnt1* insertion mutants of *MtBRI1*. Characterization of the mutants and the gene expression analysis indicated that *MtBRI1* is the BR receptor and plays important and conserved roles in the BR signaling pathway for plant growth and defense in *M. truncatula*.

## Results

### *MtBRI1* is the orthologue of *BRI1* in *M. truncatula*

**Figure 1:**
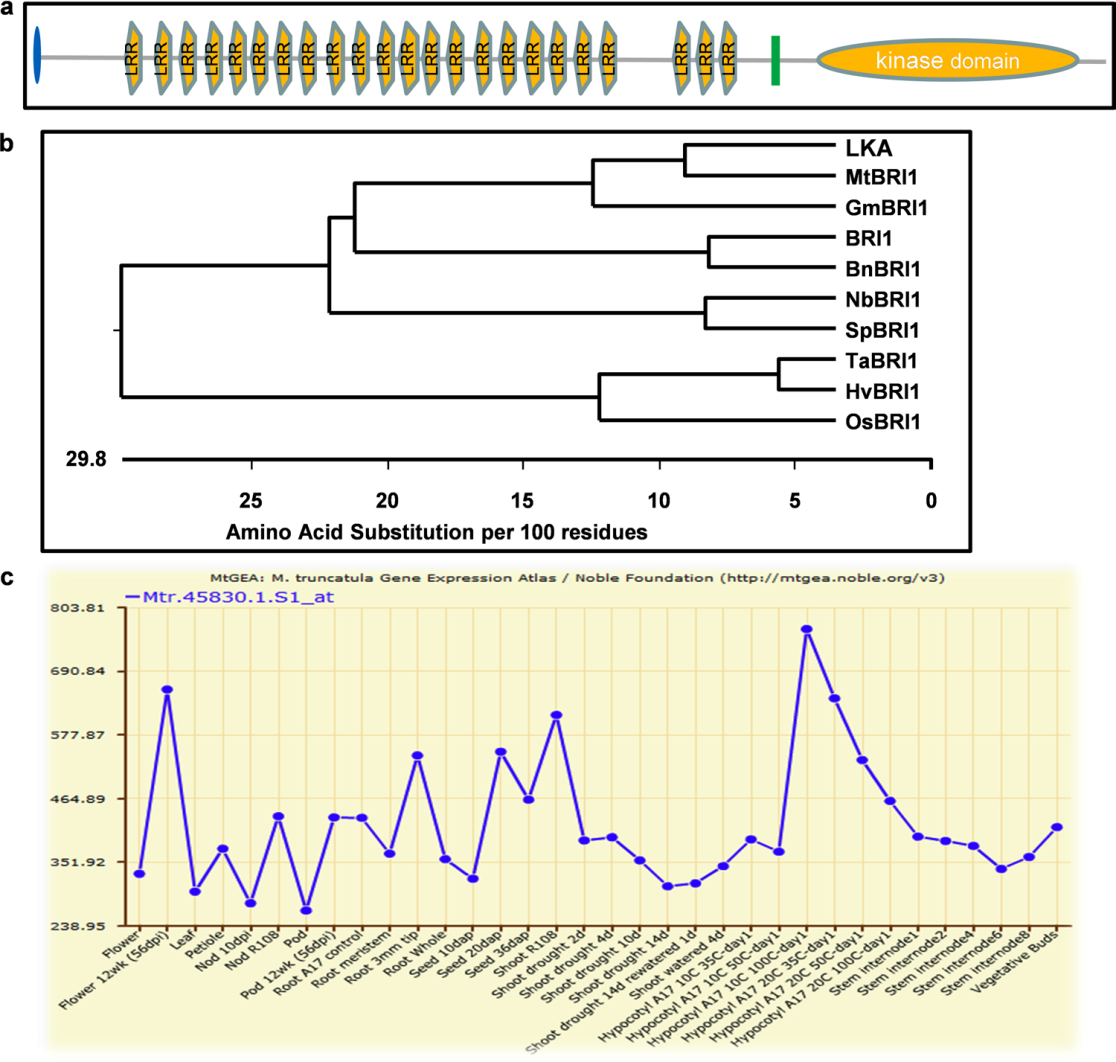
(**a**) MtBRI1 protein structure showing leucine-rich domains (LRR) and the kinase domain analysed in the Expysa-Prosite website.  Represents the signal peptide;  represents the transmembrane domain. (**b**) Phylogenetic tree of MtBRI1and other known BRI proteins analysed using the MegAlign software (DNAstar). LKA, pea, AB104529.1; GmBRI1: soybean, FJ014768.1; BRI1: Arabidopsis, NM120100.2; BnBRI1: *Brasssica napus*, JX868513.1; NbBRI1: *Nicotiana benthamiana*, EF471738.1; SpBRI1: *Solanum pimpinellifolium*, EF471736.1; TaBRI1: *Triticum aestivum*, DQ655711.1; HvBRI1: *Hordeum vulgare*, AB088206.1; OsBRI1: *Oryza sativa (Japonica)*, NM_001050612.1. (**c**) *MtBRI* expression patterns from the Medicago Gene Expression Atlas, which is represent by probe set Mtr.45830.1.S1_at.

By searching the genome sequence database of *M. truncatula*, an LRR receptor-like protein kinase (MTR3g095100) that has high homology with Arabidopsis BRI1, was identified and designated MtBRI1. The genomic sequence of *MtBRI1* is 3564 base pairs without introns and the deduced protein consists of 1188 amino acids, containing a signal peptide, 23 extracellular leucine-rich repeats, a transmembrane domain, and a cytoplasmic serine/threonine kinase domain at its C-terminus (Fig. 1a). MtBRI1 shares 67% and 88% identity at the amino acid level with Arabidopsis BRI1 and pea orthologue LKA, respectively. Phylogenetic analysis of known BRI1 proteins indicated that MtBRI1 falls into the same clade with orthologues from two other legume species pea (LKA) and soybean (GmBRI1) (Fig. 1b). According to *Medicago truncatula* Gene Expression Atlas (MtGEA), *MtBRI1* is expressed in all tissue types (Fig. 1c).

### Isolation of *mtbri1* mutants from the *Tnt1* insertion population in *M. truncatula*

To better understand the functions of *MtBRI1*, we took advantage of the *M. truncatula Tnt1* insertion mutant population generated at the Samuel Roberts Noble Foundation to identify *MtBRI1* mutants. Based on the *MtBRI1* genomic sequence, primers were designed and PCR-based reverse screening was performed in pooled genomic DNAs from 21,000 *Tnt1* insertion lines following the standard screening protocol^39, 40^. Three independent *Tnt1* insertion mutant lines, *mtbri1-1*, *mtbri1-2*, and *mtbri1-3*, were identified for *MtBRI1*. Sequence alignment indicated that *Tnt1* inserts at 709^th^ bp, 15^th^ bp and 468^th^ bp downstream of the start codon in the three mutants, respectively (see Supplementary Fig. S1). Homozygosity of *Tnt1* insertions was confirmed by genotyping (see Supplementary Fig. S1) and by semi-quantitative RT-PCR analysis with gene-specific primer pairs flanking *Tnt1* insertion sites (see Supplementary Fig. S1). From the results of RT-PCR, no *MtBRI1* transcript was detected by using the primer pairs flanking the *Tnt1* insertions (see Supplementary Fig. S1).

### Phenotypes of *mtbri1* mutants

**Figure 2:**
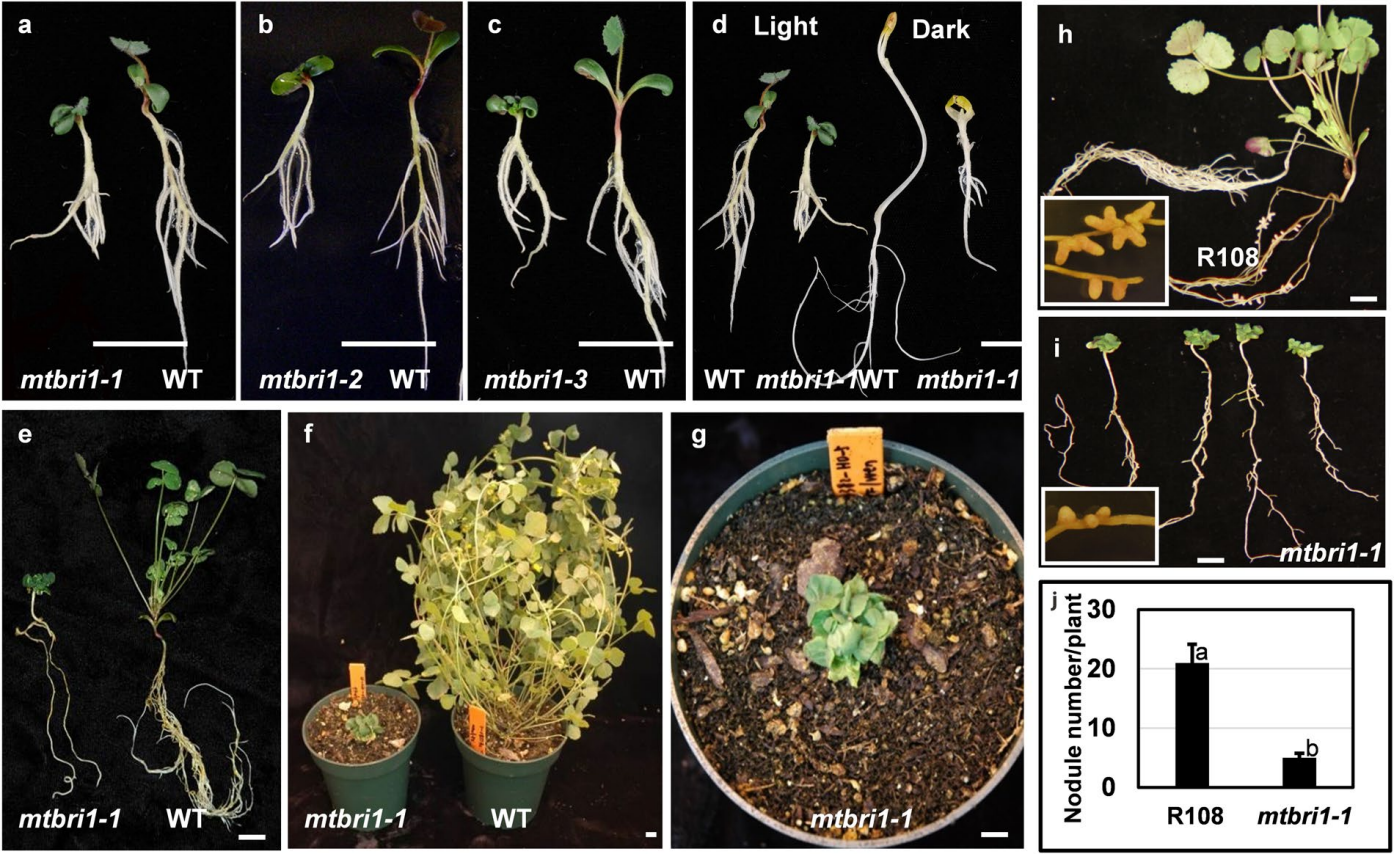
Phenotypes of *mtbri1* mutants. (**a–c**) 8-day-old seedlings grown on ½ MS media under light. (**a**) *mtbri1-1* & wild type (WT); (**b**) *mtbri1-2* & WT; (**c**) *mtbri1-3* & WT. (**d**) Comparison of 8-day-old seedlings between wild type and mutant seedlings grown under light or dark. (**e**) One-month-old *mtbri1-1* and wild type plants grown in soil. (**f,g**) Two-month-old *mtbri1-1* and wild type plants grown in soil. (**h**) Wild type R108 plants at 21 days post-inoculation with nodules shown in the inset. (**i**) *mtbri1-1* plants at 21 days post-inoculation with nodules shown in the inset. (**j**) Nodule numbers per plant in wild type R108 and *mtbri1-1*. Error bars represent the standard error of the means (*n* ≥ 10). Means with different letters are significantly different (P < 0.005; Tukey’s test). Scale bars: 10 mm.

Though *Tnt1* inserts are at different locations of *MtBRI1*, all three *mtbri1* mutant alleles display similar defective phenotypes: extreme dwarfism and compact shoots with curly dark green leaves, shortened leaf blades and petioles, and reduced lateral roots (Fig. 2a–c). No elongated stems are formed (Fig. 2e,g). Flowers are rarely observed and no pods are produced in all mutants (Fig. 2f). The dark grown *mtbri1* seedlings exhibit typical photomorphogenesis with short hypocotyls and open cotyledons, in contrast to the etiolated morphology with elongated hypocotyls and closed cotyledons in wild type seedlings (Fig. 2d). Interestingly, *mtbri1* mutants have significantly reduced nodule numbers compared to wild type plants (Fig. 2h–j). In addition, unlike pink nodules in wild type plants, nodules in *mtbri1* mutants are small and mostly white, which are defective in nitrogen fixation (Fig. 2h,i, insets). This phenotype is different from the results observed by Ferguson *et al*.^41^ in another legume species, pea. Ferguson *et al*. only observed reduced nodule number but not reduced nodule size and the nitrogen fixation activity in brassinosteroid responsive mutant *lka*. In *mtbri1* mutants, both total nodule number and nodule size are reduced. The small nodules in *mtbri1* mutants are also defective in nitrogen fixation.

### *mtbri1* seedlings are insensitive to brassinolide and brassinazole treatment

**Figure 3:**
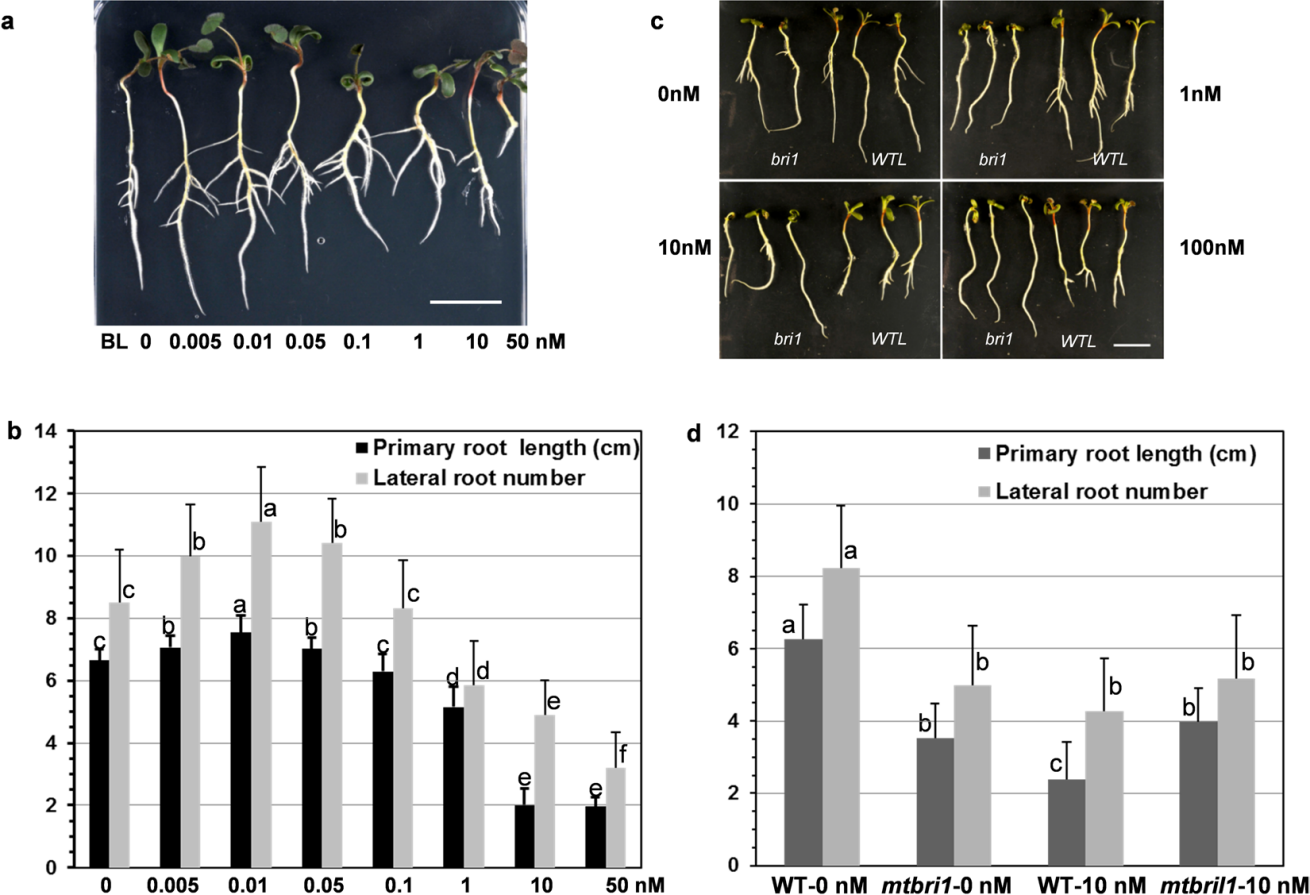
Effects of exogenous BL on the primary root length and lateral root number of wild type (WT) and *mtbri1* seedlings. (**a**) Germinated wild type seeds were grown on ½ MS media supplemented with 0, 0.005, 0.01, 0.05, 0.1, 1.0, 10, or 50 nM BL for 7 days (one representative plant is shown for each concentration); (**b**) The primary root length and the lateral root number of wild type seedlings grown on the media with a series concentration of BL. Error bars represent the standard error of the means (*n* ≥ 20). Means with different letters are significantly different (P < 0.005; Tukey’s test). (**c**) *mtbri1-1* and wild type-like (WTL) seedlings grown on ½ MS media supplemented with 0, 1.0, 10, or 100 nM BL for 7 days; (**d**) Comparison of the primary root length and lateral root number in *mtbri1-1* and wild type-like seedlings grown on ½ MS media with 0 and 10 nM BL for 7 days. Error bars represent the standard error of the means (*n* ≥ 20). Means with different letters are significantly different (P < 0.005; Tukey’s test). Scale bars: 20 mm.

Exogenous application of certain amount of bioactive brassinolide (BL) promotes plant growth. To examine the responsiveness of wild type R108 seedlings to exogenous BL application, we grew germinated seeds on the ½ MS media supplemented with BL at a series of concentrations (0, 0.005, 0.01, 0.05, 0.1, 1.0, 10, and 50 nM) for seven days and measured the primary root length and counted lateral root numbers. Seedlings grown on media containing 0.01 to 0.05 nM of BL showed longer primary roots and more lateral roots than the control seedlings (no BL), whereas the primary root length and the lateral root number gradually decreased with further increase of BL concentrations. Seedlings grown on MS media containing 10 nM BL exhibited very short primary roots and only a few lateral roots, resembling the root phenotypes of *mtbri1* mutants (Fig. 3a,b). The results indicated that BL at low concentrations (≤0.05 nM) promotes the primary root elongation and lateral root formation, whereas at high concentrations BL inhibits both primary root elongation and lateral root formation in *M. truncatula*.

To understand how *mtbri1* mutants respond to exogenous BL application, we also grew *mtbri1* mutant seedlings on MS media with a series of BL concentrations. In contrast to the inhibited primary root elongation in wild type seedlings, no apparent difference was observed in both primary root length and lateral root number of *mtbri1* mutants grown on various BL concentrations (Fig. 3c,d), indicating that *mtbri1* mutants are insensitive to exogenous BL treatment.

Brassinazole (BRZ) is a specific inhibitor of BR biosynthesis and inhibits the hypocotyl elongation in both light and dark in Arabidopsis^7, 27, 42^. To confirm the responses of *M. truncatula* wild type and *mtbri1* mutants to BRZ, we grew germinated seeds on ½ MS media containing different concentrations of BRZ in light or in the dark for five days. Similarly as observed in Arabidopsis, BRZ decreases the hypocotyl length of wild type seedlings by 60% and 56% under light and dark conditions, respectively (see Supplementary Fig. S2). The hypocotyl length of *mtbri1* mutants is very short and show no significant difference with or without BRZ treatment (see Supplementary Fig. S2). In addition, under dark conditions, in contrary to the closed cotyledons of the control seedlings (no BRZ), the BRZ-treated wild type seedlings have opened cotyledons, resembling the phenotype of *mtbri1* mutants (see Supplementary Fig. S2). Under both light and dark conditions, *mtbri1* mutants are insensitive to the BRZ treatment (see Supplementary Fig. S2).

### *MtBRI1* complements Arabidopsis *BRI1* mutant *bri1-5*

**Figure 4:**
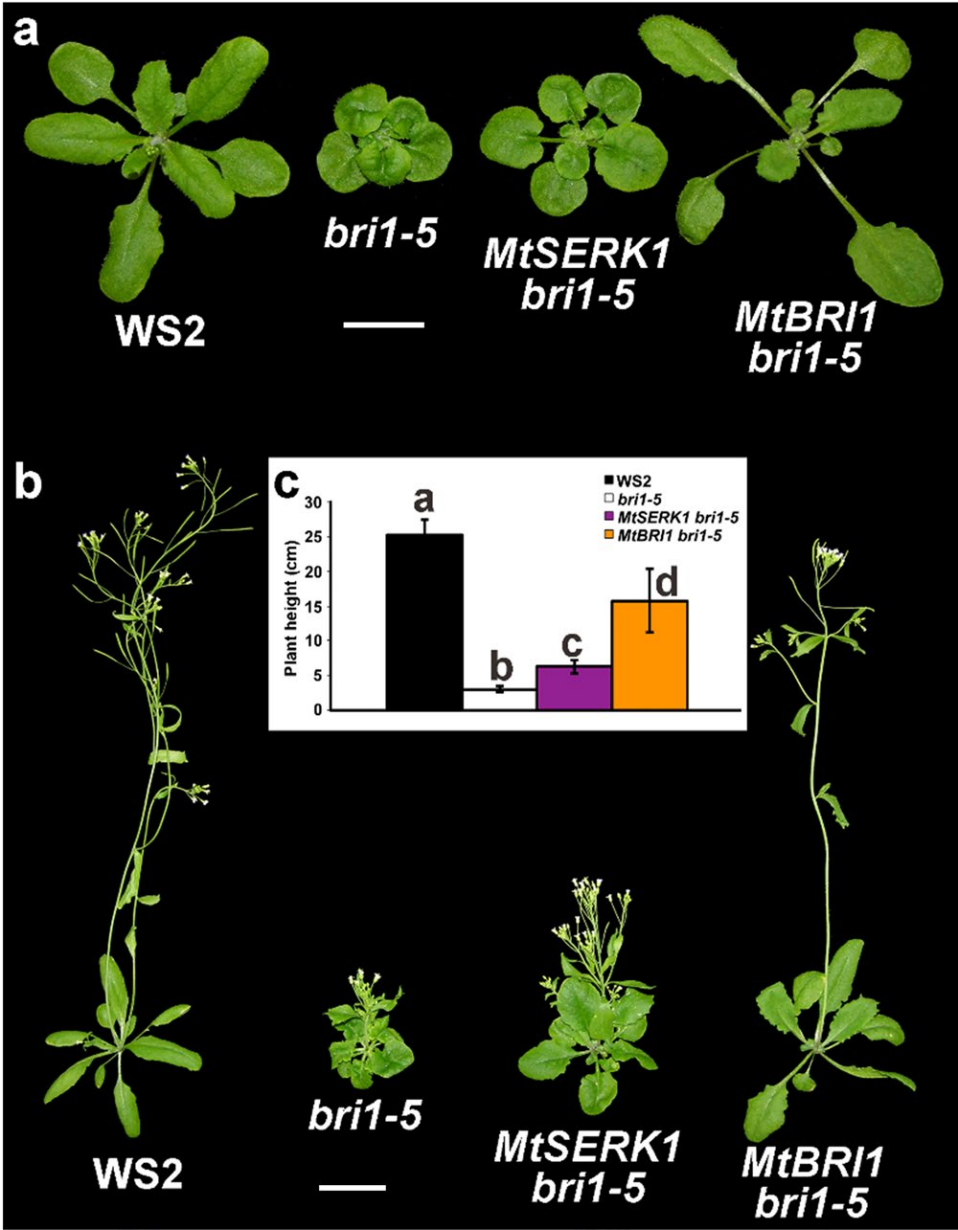
Overexpression of *MtSERK1* and *MtBRI1* suppresses the defective phenotypes of *bri1-5*. (**a**) 18-day-old plants. (**b**) 30-day-old plants. (**c**) Measurements of the plants shown in b. Error bars represent standard error (n > 20). Means with different letters are significantly different (P < 0.005; Tukey’s test). Scale bars: 2 cm.

*bri1-5* is a weak mutant allele of Arabidopsis *BRI1*, containing a point mutation resulting in a C69Y amino acid substitution in the extracellular domain of BRI1^31^. Compared to null alleles of *bri1* mutants, *bri1-5* is semi-dwarf and sets nearly normal amount of seeds, making it an ideal material for BRI1 complementation studies. We transformed *bri1-5* plants with the *MtBRI1* cDNA and the transgenic plants showed nearly complete complementation of the *bri1-5* mutant phenotypes (Fig. 4). The *MtBRI1* complemented transformants resembled those of *BRI1* complemented transgenic lines with elongated petioles of rosette leaves (Fig. 4a), indicating the conserved functionality of MtBRI1. We also cloned the orthologue of Arabidopsis *BAK1*, *MtSERK1*, from *M. truncatula* and introduced it into *bri1-5* plants. The transgenic plants harboring *MtSERK1* partially suppressed the *bri1-5* mutant phenotypes (Fig. 4a), similar to the partial complementation of *BAK1* ^21^.

### MtBRI1 interacts with MtSERK1 *in vivo*

**Figure 5:**
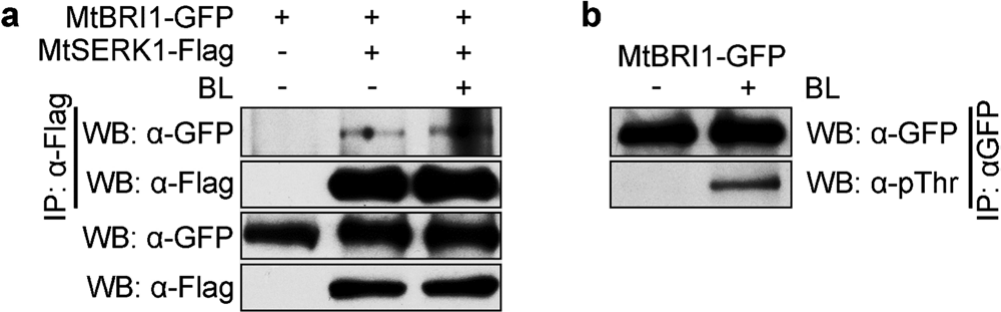
MtSERK1 interacts with MtBRI1 and MtBRI1 responds to exogenously applied BR. (**a**) Co-immunoprecipitation result indicates that MtSERK1 interact with MtBRI1 *in vivo*. (**b**) The phosphorylation of MtBRI1 is elevated in response to BR treatment. Blot images were cropped for better display and comparison. For the full-length western blots see Supplementary Figure S6.

It has been well established that BAK1 is a co-receptor of BRs in Arabidopsis and interacts with BRI1 to initiate the BR signal transduction. To confirm whether MtBRI1 interacts with MtSERK1 *in vivo*, we separately transformed GFP-tagged *MtBRI1* and FLAG-tagged *MtSERK1* into Arabidopsis mutant *bri1-5*. Transformants of either *MtBRI1-GFP* or *MtSERK1-FLAG* suppresses *bri1-5* phenotypes (Fig. 4a,b). Transgenic *MtBRI1-GFP bri1-5* and *MtSERK1-FLAG bri1-5* plants were crossed to generate a double transgenic plant harboring both *MtBRI1-GFP* and *MtSERK1-FLAG*. MtSERK1-FLAG and MtBRI1-GFP in the membrane fraction were immunoprecipitated with either agarose-linked α-FLAG antibody or α-GFP antibody. The immunoprecipitated proteins were detected by both α-FLAG antibody and α-GFP antibody (Fig. 5), indicating *in vivo* interaction of MtBRI1 and MtSERK1 in Arabidopsis. However, the interaction was not enhanced by BL treatment, though the phosphorylation of MtBRI1 was elevated by BL application (Fig. 5b).

### Global gene expression changes in *mtbri1* mutants by Affymetrix microarray and MapMan analysis

**Figure 6:**
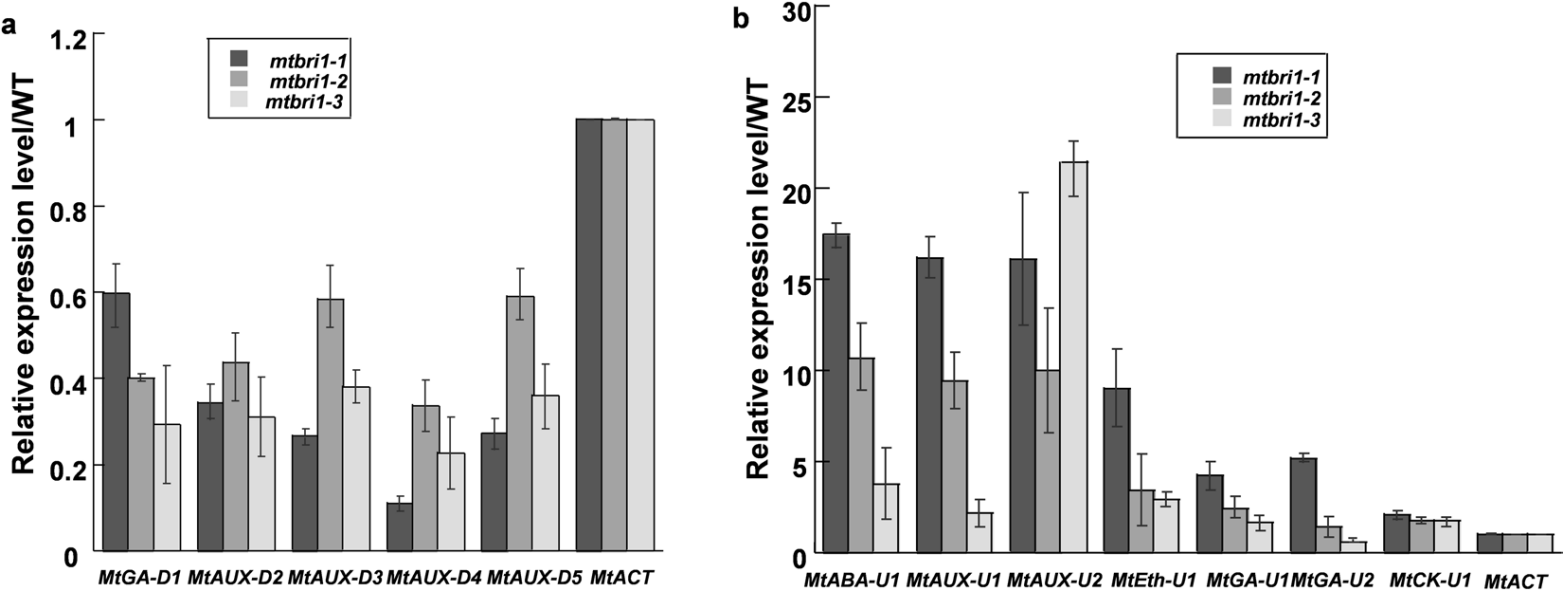
qRT-PCR verification of gene expression levels in three *mtbri1* mutants. (**a**) Expression levels of five down-regulated genes; (**b**) Expression levels of seven up-regulated genes. *MtACT* was used as the internal control.

The mutation of *MtBRI1* leads to dramatic pleiotropic phenotypes in *M. truncatula*. To further decipher the underlying molecular mechanism, we analysed the overall gene expression in 10-day-old seedlings of three *mtbri1* mutants relative to the corresponding wild type-like seedlings from the same segregating progeny by Affymetrix microarray. Totally, 603 genes were up-regulated and 312 genes were down-regulated by at least 2-fold changes in all the three mutant alleles (see Supplementary Table S1). The microarray results were validated by qRT-PCR in 12 out of 15 genes related to hormone metabolism, while the other three genes were expressed at very low levels (Fig. 6).

**Figure 7:**
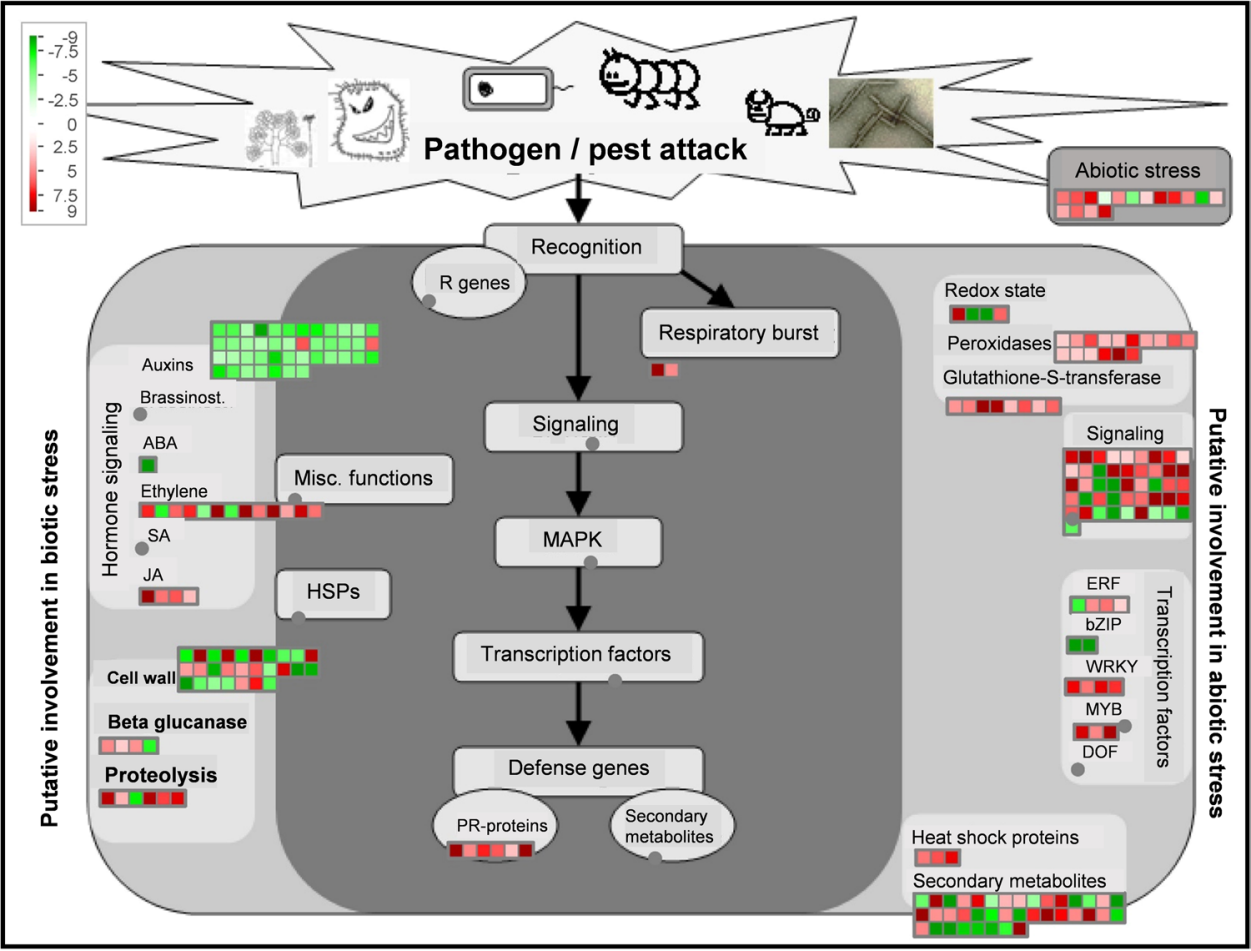
Visualization of differentially expressed genes mapped into the biotic stress pathway with MapMan3.5.

**Figure 8:**
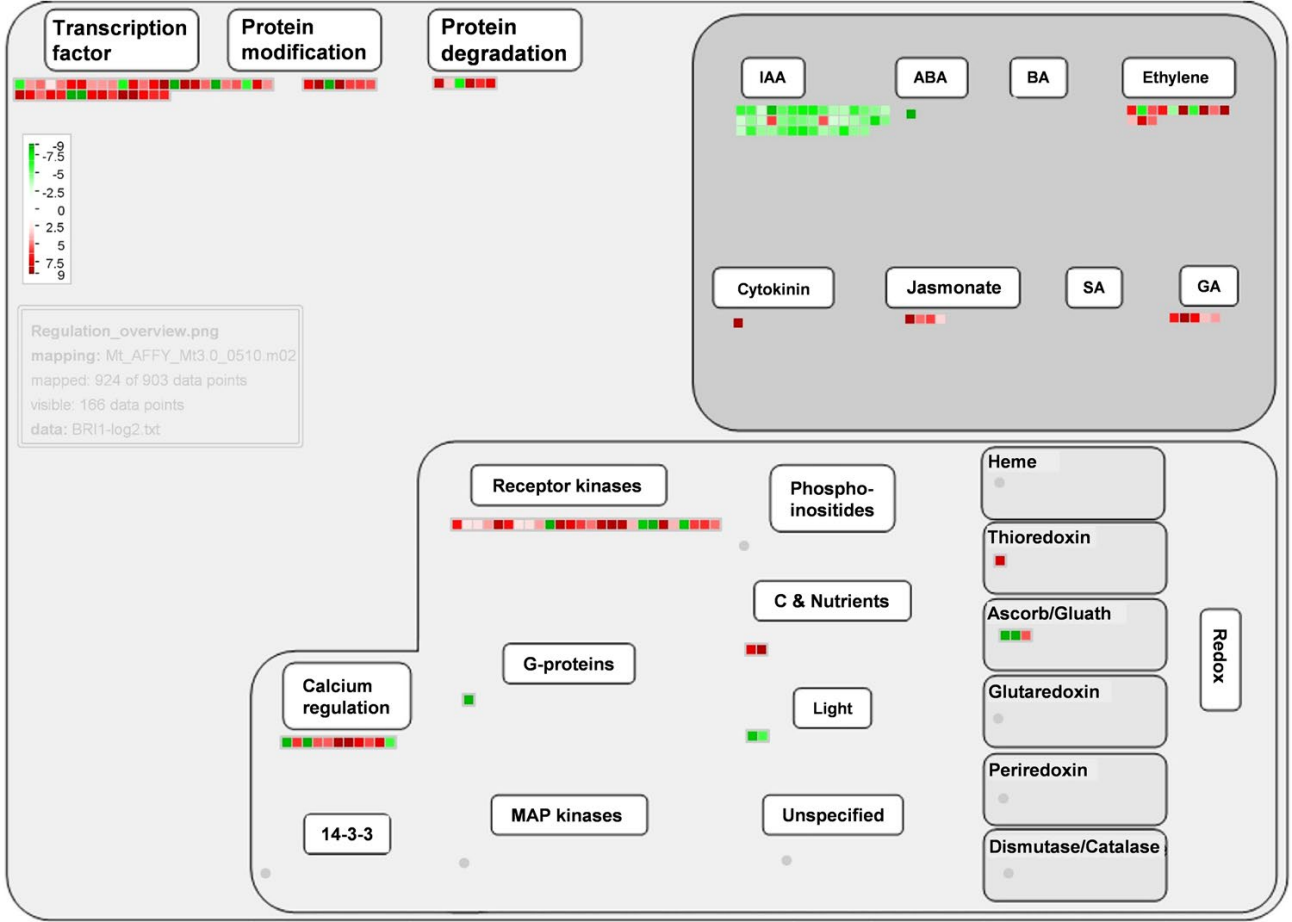
Visualization of differentially expressed genes mapped into the regulation pathway with MapMan3.5.

To better visualize and understand the 915 regulated genes in *mtbri1* mutants, the fold-change values (≥2 or ≤0.5, P ≤ 0.05) from the microarray analysis were converted into log2 values and input to the MapMan software program. The overview pathway by MapMan analysis revealed that 903 out of the 915 regulated genes, which show 924 data points, are mapped and classified to various functional categories and half of them are grouped into unknown functions (see Supplementary Table S2). Among the functionally classified genes, except for a large group of miscellaneous, hormone metabolism, RNA regulation, stress, secondary metabolism, cell wall, and signaling genes are over-represented (see Supplementary Fig. S3). When these genes are mapped into specific pathways by MapMan, two pathways are standout: one is the biotic stress pathway, where 250 out of the 924 data points are mapped (Fig. 7); the other is the regulation pathway, where 190 points are mapped (Fig. 8). Interestingly, a group of 43 auxin-responsive genes, bin 17.2, are significantly regulated in both biotic stress and regulation pathways (Figs 7, 8). Among them, two P450 reductase genes are up-regulated but the other 41 genes, including one auxin-amino acid transferase gene *GH3.3* and 40 *SAUR* (small auxin-upregulated RNA) genes, are down-regulated. The *SAURs* share more than 80% sequence identity among each other and share some similarity to *SAUR14* and *SAUR-AC1*. Nine out of 40 SAUR genes are located between Medtr3g084150 to Medtr3g084250; 30 are clustered between Medtr4g072220.1 to Medtr4g072980.1. Based on MtGEA, most of these *SAUR* genes show similar expression patterns and are detectable in shoots, buds, leaves, flowers, and pods. They are highly expressed in hypocotyls but lowly expressed in roots. They all respond to drought treatments (see Supplementary Fig. S4).

### Expression of BR signaling pathway genes in *mtbri1* mutants

The BR signaling cascade is initiated by BRs binding to BRI1, followed by sequential phosphorylation to BR transcription factors BZR1/2. To understand the response of the gene expression to the impairment of BR signal initiation in *M. truncatula*, we searched the *Medicago* genome database and obtained the following BR signaling pathway orthologous genes in *M. truncatula*: *MtBSK1*, *MtCDG1*, *MtBSU1*, *MtBIN2*, *MtPP2A*, *MtBZR1* and *MtBZR2*. All these genes have represented probe sets in Medicago Affychip, but none of the probe sets was present in the 2-fold up or down change list in the microarray analysis result. By searching the individual gene expression results, *MtBSK1*, *MtCDG1*, *MtPP2A*, *MtBIN2* and *MtBSU1*, which are in the upstream of the signaling pathway, show the expression ratio value (*mtbri1*/WT) close to 1 (see Supplementary Table S3). However, the transcription regulators, *MtBZR1* and *MtBZR2*, show changes in expression levels. *MtBZR1*, which is represented by 3 probe sets, is down-regulated by ~0.75 fold, whereas *MtBZR2* is up-regulated by ~1.5 fold in *mtbri1* mutants (see Supplementary Table S3). The microarray results were further confirmed by qRT-PCR (see Supplementary Fig. S5). In summary, mutations in *MtBRI1* have no effects on the expression of most genes involved in BR signaling pathway, whereas the expression of two core transcription regulators MtBZR1/2 is regulated in opposite directions.

### Regulation of BR metabolism genes in *mtbri1* mutants

The BR biosynthesis pathway is subject to feedback regulation at multiple points to ensure homeostasis of endogenous BRs. To reveal the expression of BR metabolic genes in *mtbri1* mutants, we searched *Medicago* genome database for orthologues of known genes involved in the BR biosynthetic pathway. Six biosynthetic genes that are orthologous to corresponding Arabidopsis genes, *MtBrox1, MtBrox2, MtCPD*, *MtDWF4*, *MtDET2*, and *MtDET2-1*, were retrieved. In addition, we also identified *MtBAS1*, the orthologue of Arabidopsis *BAS1*, which is involved in BR inactivation and photomorphogenesis. Microarray analysis results showed that the expression of *MtDWF4* is increased by 5.6 fold; the expression of *MtDET2* is slightly decreased to 0.88 fold; whereas the expression of *MtBAS1* is decreased to ~0.36 fold in *mtbri1* mutants (see Supplementary Table S3). No probe sets corresponding to *MtBrox1, MtBrox2* and *MtCPD* in *Medicago* Affychip were found and the expression of these genes were analysed by qRT-PCR. The results showed that the expression of these three genes is increased in *mtbri1* mutants. Furthermore, microarray results of *MtDWF4*, *MtDET2*, and *MtBAS1* was also confirmed by qRT-PCR (see Supplementary Fig. S5). The expression pattern analysis indicated that the BR biosynthesis pathway is enhanced, whereas the BR inactivation by MtBAS1 is inhibited in *mtbri1* mutants.

### E-box is the potential common DNA binding domain for BR signaling targets

*BZR1/2* are two core transcription factors in the BR signaling pathway and BZR1 directly or indirectly regulates the expression of about 80% of the genes downstream of *BRI1*. Both *BZR1* and *BZR2* bind to BR responsive element (BRRE) (CGTGT/CG) and E-box (CANNTG) in promoters of BR responsive genes^43, 44^. In this study, gene expression analysis of *mtbri1* mutants revealed the genes directly or indirectly regulated by BR signaling. To get more insights of potential BR targeted genes, we scanned 1 kb genomic sequences upstream of ATG of well-known BZR1/2 target genes for BRRE and E-box elements. First, we scanned for BRRE and E-box in promoter regions of BR down-regulated *SAUR* genes. Two to eleven E-boxes are located in the promoter regions of all 40 *SAUR* genes, with most of the E-boxes inside 200 bp upstream of ATG. For three other auxin-responsive down-regulated genes, one contains two E-boxes, while the other two, including GH3.3, contains no E-box in the 1 kb promoter region. No BRRE was detected in the 1 kb promoter region of all genes examined (see Supplementary Table S4).

We further scanned for BRRE and E-box in the promoter region of up-regulated BR biosynthetic genes and other hormone related genes. Two or more E-boxes are located in the promoter region for BR biosynthetic genes. E-boxes are enriched in all auxin responsive genes. We also found E-boxes in the promoters of three gibberellin-related genes and ethylene-related genes (see Supplementary Table S4). Again, BRRE was not found in the scanned promoter regions of all examined genes, except that one BRRE motif in the *MtCPD* promoter. Therefore, E-boxes are enriched in the promoter regions of BR-regulated genes, and it is the potential common DNA binding element in *MtBZR1/2* targets.

## Discussion

In this study, we demonstrated that *MtBRI1* shares high sequence and protein structure similarity with BR receptors from other plant species; mutations in *MtBRI1* impair the BR signaling pathway and lead to the characteristic developmentally defective phenotypes of *bri1* mutants. Moreover, mutations in *MtBRI1* cause defective nodule development and nitrogen fixation, which is a novel observation in *bri1* mutants. MtBRI1 not only suppresses the defects of Arabidopsis mutant *bri1-5*, but interacts with MtSERK1 *in vivo* as well. In agreement with previous microarray reports, typical BR signaling target genes, such as *SAUR* genes, and BR biosynthetic genes are differentially regulated in *mtbri1* mutants. Over-represented hormone related genes imply that extensive crosstalk exists among different hormone pathways, which is consistent with the dramatic pleiotropic phenotypes of *mtbri1* mutants. Collectively, the results indicated that *MtBRI1* is a BR receptor in *M. truncatula* and has conserved functions as other *BRI1* genes; and the BR signaling plays important roles in plant growth, development, and defense in *M. truncatula*.

BZR1 and BES1/BZR2 are two core transcription factors in the BR signaling pathway. Activation of either *BZR1* or *BES1/BZR2* by dominant mutation suppresses the phenotypes of *bri1* in Arabidopsis. Microarray analysis of *bzr1-1D* and *bri1* mutants indicated that *BZR1* regulates directly or indirectly the expression of 80% of the genes downstream of *BRI1* ^27, 28, 45, 46^. BZR1 and BES1/BZR2 share 88% overall amino acid identity and 97% identity in the DNA-binding domain. Both BZR1 and BES1/BZR2 bind to BR responsive element (BRRE) and E-box present in promoters of BR responsive genes, directly or indirectly interacting with other transcription factors and functioning as transcription repressors and activators to regulate multiple developmental events^17, 47^. The exclusive existence of E-box, but not BRRE, in the promoter regions of both up- and down-regulated genes suggests that E-box is potentially the common DNA-binding motif for both *MtBZR1* and *MtBZR2* to activate or repress the expression of downstream BR signaling genes in *M. truncatula*. Enriched E-box motif in the promoter region of known BR regulated genes indicates that these genes are also the potential targets of *MtBZR1/2*. In *mtbri1* mutants, the expression of *MtBZR1* and *MtBZR2*, is altered in opposite ways. If E-box is the common DNA binding motif, *MtBZR1* and *MtBZR2* may have different regulatory mechanisms. The decreased expression of *MtBZR1* in *mtbri1* may relieve the repression of BR feedback regulation, which supports that *BZR1* plays a role as a transcriptional repressor. The increased expression of *MtBZR2* promotes BR signaling in *mtbri1* mutants, supporting that *BZR2* functions as a transcriptional activator.

In nature, plants constantly encounter changing external and internal environmental cues. It is crucial for plants to adjust and maintain the balance between growth and defense in response to diverse and complex signals. In the crosstalk between defense signaling and growth hormones, BR, salicylic acid, and auxin play important roles in the plant growth-defense tradeoff^48, 49^. It is suggested that BR-mediated growth directly antagonizes innate immune signaling. On one hand, the competition between BRI1 and the flagellin receptor FLS2 for the association of co-receptor BAK1 may facilitate BR-mediated suppression of PR1-mediated defense^50, 51^; on the other hand, BZR1 targets a group of downstream WRKY genes to inhibit PTI (pathogen-associated molecular pattern triggered immunity) and interacts with other hormone signaling transcription factors to mediate plant growth^48^. In *mtbri1* mutants, a large number of genes potentially involved in the biotic stress pathway are differentially regulated. On the defense side, when *MtBRI1* is disrupted, most defense related genes are up-regulated, including genes in defense hormone ethylene/JA pathways, such as signaling components, ERF, WKRY and MYB transcription factors, respiratory burst genes, PR proteins, and ROS reaction genes. Some putative chitinases, glucosidases and glucanases, which were reported to act synergistically to degrade fungal cell walls^52^, are also up-regulated. The results suggest that the entire defense system from hormone signaling, signal transduction, transcription to metabolism is enhanced in *mtbri1* mutants. On the growth side, the auxin-responsive gene *GH3.3* is upregulated and a group of *SAUR* genes are down-regulated in *mtbri1* mutants. GH3 enzymes inactivate IAA by forming conjugates with amino acids. Overexpression of several *GH3* genes retards plant growth. It is suggested that the GH3-mediated growth suppression directs re-allocation of metabolic resources to resistance establishment and represents the fitness costs of induced resistance^48, 53^. *SAURs* are members of a large multigene family in the Arabidopsis genome comprising of 72 members and are early auxin responsive genes with unclear functions. The expression of *SAUR*s correlates well with both auxin-induced and BR-mediated cell elongation. It has been reported that *SAUR19* has functions in cell expansion, *SAUR6*3 promotes hypocotyl and stamen filament elongation, and *AAM1* is related to apical hook development^54–57^. In *M. truncatula*, *SAURs* are highly expressed in elongating tissues, indicating they may also have functions in cell elongation or expansion to mediate plant growth. Combining with the dwarf phenotype, it is indicated that plant growth is compromised in *mtbri1* mutants. Taken together, we concluded that the repression of plant growth caused by BR signaling deficiency triggers the plant biotic defense system and turns the plant from growth into the defense mode. Recently, it is reported that mutation of *BRI* enhances plant resistance to fungal and viral pathogens in both barley and *B. distachyon* ^38, 58^. However, how *mtbri1* mutants respond to pathogen attack needs to be tested in future experiments.

Nodulation is a unique feature for legume species. In pea, there is no difference in nodule size and nitrogen fixation activity between wild type and BR response mutant *lka*. Disruption of BR signaling only reduces the nodule number. However, in *M. truncatula*, mutation in *MtBRI1* leads to reductions in nodule number, nodule size and nitrogen fixation activity. The mechanism of *MtBRI1* involvement in nodulation will be further investigated in the future. Since all the *mtbri1* mutants are extremely small, weak mutant alleles from the BR signaling pathway could be better to study the function of *BRI1* in legume plant development.

## Material and Method

### Seed treatment and seedling growth

Seeds of wild type *M. truncatula* R108 and *mtbri1* heterozygous lines were scarified with concentrated sulfuric acid for 8 minutes and rinsed with water, followed by sterilization in 30% bleach for 10 minutes, rinse with ddH_2_O, germinated for 2 days in dark at room temperature, and then transferred the germinated seeds onto ½ MS medium and grown in a growth chamber with 18-h-light/25 °C and 6-h-dark/ 22 °C photoperiod. For microarray experiments, shoots of 7-day-old seedlings of wild type like (including wild type and heterozygous plants in the same segregating progeny) and mutants were sampled. After 2 weeks, seedlings were transferred into Metro-Mix 350 (Scotts) composite soil and grown in the greenhouse until maturation.

All Arabidopsis plants including WS2, Col, *bri1-5*, and corresponding transgenic plants were grown at 22 °C in a long-day condition (16 h of light and 8 h of dark) in a greenhouse.

### Nodulation of *M. truncatula* plants

Germinated wild type R108 and *mtbri1* seedlings were transferred to plastic cones containing a 2:1 ratio of turface/vermiculite. Plants were cultivated under a 16-h/8-h light/dark regime with 200 μE m^−2^ s^−1^ light irradiance at 21 °C and 40% relative humidity. After seven days of growth under normal nitrogen condition, plants in each cone were inoculated with 50 mL of *Sinorhizobium meliloti* strains *Sm1021*. Rhizobia *S*. *meliloti* were grown overnight in TY (tryptone yeast extract) liquid medium, with shaking at 250 rpm, to OD_600_ approximately 1.0, pelleted by centrifugation, and resuspended in half-strength Broughton and Dilworth (BD) solution with 0.5 mM KNO_3_ at OD_600_ approximately 0.02 for inoculation. After inoculation, plants were watered once a week with half-strength BD solution with 0.5 mM KNO_3_ for three weeks. At 21 days post-inoculation, plant roots were washed in water for nodule phenotyping.

### PCR-based reverse screening for *mtbri1* mutants in *Tnt1* insertion population in *M. truncatula*

*MtBRI1* genomic sequence was retrieved by searching the *M. truncatula* genome database using the Arabidopsis *BRI1* sequence. Two pairs of primers (MtBRI1-F, MtBRI1-F1, MtBRI1-R and MtBRI1-R1, see sequences in Supplementary Table S5) were designed at 5′ and 3′ end of the *MtBRI1* genomic sequence. PCR-based reverse screening was carried out in *Tnt1* insertion population^59^ as described previously^39, 40^.

### Brassinolide and brassinazole treatment

After sterilization, the seeds of wild type *M. truncatula* R108 and mutant lines were germinated on filter paper in dark for two days. The germinated seeds were transferred to solidified ½ MS medium supplemented with various concentrations of BL (0, 0.005, 0.01, 0.05, 0.1, 1, 10, 50, and 100 nM) or BRZ (0, 1, 5 μM). Seedlings were grown in a growth chamber with a regime of 18-h-light/25 °C and 6-h-dark/ 22 °C photoperiod for seven (for BL) or five (for BRZ) days. For BRZ treatment in dark, seedlings (wild type and *mtbri1* mutant) were vertically grown on plates covered with aluminum foil for five days in the same growth chamber as for light treatment. The primary root length of the seedlings was measured and the lateral root number was counted for BL treatment. For BRZ-treated seedlings, the hypocotyl length was measured.

### Complementary DNA cloning and plant transformation

The coding sequences of *M. truncatula SERK1* (*MtSERK1*) and *BRI1* (*MtBRI1*) were cloned with the Gateway technology for overexpression in *bri1-5* and Col using primers MtBRI1PB1, MtBRI1PB2, MtSERK1PB1, and MtSERK1PB2. The amplified CDS sequences were introduced into the destination vectors pB35GWF and pH35GWG with the help of Gateway technology^60^. The cloned sequences were confirmed by DNA sequencing and Arabidopsis plants were transformed by the floral dip method.

### Membrane protein extraction, co-immunoprecipitation and western blot analysis

Nine-day-old liquid-cultured seedlings of transgenic plants harboring *35 S::MtSERK1-*FLAG, or *35 S::MtSERK1-*FLAG and 35 S*::MtBRI1-GFP* transgenes were treated with or without 24-epiBL for 90 min and ground to fine powder in liquid nitrogen for total protein preparation. MtSERK1-FLAG was immunoprecipitated with agarose-linked α-FLAG antibody (Sigma, St. Louis, MO). For phosphorylation analysis, transgenic plants of *35 S::MtBRI1-GFP* were treated with mock solution or 24-epiBL for 90 min, and ground to fine powder in liquid nitrogen for membrane protein isolation^21^. MtBRI1-GFP was immunoprecipitated with the α-GFP antibody (Invitrogen, Carlsbad, CA) and protein G beads (Roche, Indianapolis, IN). The immunoprecipitated proteins were separated on 7.5% SDS polyacrylamide gel for western blot analyses with α-GFP, α-FLAG or α-phosphothreonine antibodies as previously described^60^.

### Microarray analysis and qRT-PCR

For gene expression analysis, control (wild type-like) and mutant shoot samples were harvested from 10-day-old seedlings. Three biological replicates were performed for control plants and each of the homozygous *mtbri1-1, mtbri1-2* and *mtbri1-3* plants. Total RNA was extracted using RNeasy Plant Mini Kit (Qiagen). Purified RNA was treated with Turbo DNase I (Ambion). For microarray analysis, 10 µg of total RNA from 3 control samples and 3 mutant samples of *mtbri1-1*, *mtbri1-2* and *mtbri1-3* were used for probe labeling. Hybridization and scanning for microarray analysis were conducted according to the manufacturer’s instructions (Affymetrix). Differentially expressed genes between wild type-like plants and homozygous *mtbri1* mutants were selected using associative analysis as previously described^61^. For RT-PCR and qRT-PCR, three µg of total RNA of each samples were used for reverse transcription using SuperScript III Reverse Transcriptase (Invitrogen) with oligo (dT)^20^ primer. Two µl of 1:20 diluted cDNA were used as templates. Gene-specific primers used for RT-PCR and qRT-PCR were listed in Supplementary Table S5. All qRT-PCR was carried out using a 7900HT Fast Real-Time PCR System (Applied Biosystems) and the data were analyzed using SDS 2.2.1 software (Applied Biosystems). The transcript levels were determined by relative quantification using the *M. truncatula* actin gene (tentative consensus no. 107326) as the reference gene.

## Electronic supplementary material

Supplementary Information
