# Supplementary material for: Functional characterisation of brassinosteroid receptor MtBRI1 in Medicago truncatula
Source: Sci Rep. 2017 Aug 24;7:9327. doi: 10.1038/s41598-017-09297-9 (PMC5570916; doi:10.1038/s41598-017-09297-9)
Supplement: Supplementary file 1 — Supplementary Information [file 41598_2017_9297_MOESM1_ESM.pdf]

Supplementary Information

Title: Functional characterisation of brassinosteroid receptor *MtBRI1* in *Medicago truncatula*

Xiaofei Cheng, Xiaoping Gou, Hongju Yin, Kirankumar S. Mysore, Jia Li, Jiangqi Wen

Supplementary Table S1. Microarray results showing up- and down-regulated genes.

Supplementary Table S2. Total up- or down-regulated genes analysed by MapMan.

Supplementary Table S3. Fold changes in expression of genes related to BR metabolism and signaling in microarray analysis of *mtbri1* mutants.

Supplementary Table S4. E-box and BRRE in the promoter regions of regulated genes in *mtbri1* mutants.

Supplementary Table S5. Primers used in this study.

Supplementary Figure S1. Identification of *mtbri1* mutants.

Supplementary Figure S2. Effects of brassinazole on the elongation of hypocotyls of wildtype and *mtbri1* seedlings under light and dark conditions.

Supplementary Figure S3. Overview of functional classification of the 903 differentially expressed genes.

Supplementary Figure S4. *SAUR* gene expression patterns from the Medicago Gene Expression Atlas.

Supplementary Figure S5. qRT-PCR expression analysis of genes in BR metabolism and BR signaling pathway in *mtbri1* mutants.

Supplementary Figure S6. Full-length western blots from Figure 5. Lanes used in Figure 5 are outlined with a black rectangle.

Supplementary Table S1. Microarray results showing up- and down-regulated genes. Samples S1, S3, S5 are three bilogical replicates for *mtbri1* mutants (MT). Samples S2, S4, S6 are three bilogical replates for wildtype (WT). Shoots of 10-day-old WT or MT seedlings were used for total RNA isolation. Ten micrograms of purified RNA samples were used for probe labeling. Hybridisation with Affymetrix Medicago Genechip and subsequenct scanning were conducted following the manufacturers' instructions.

IMAGA3.5 Annotation	Probesets	S2	S4	S6	S1	S3	S5	Ave WT	SD WT	Ave MT	SD MT	Pts(MT/WT)	Pta(MT/WT)	Ratio(MT/WT)
IMGA|Medtr2g045280.1	Msa.1060.1.S1_at	57.76271	40.25523	25.6835	88.7927	101.960	184.5453	41.23383	16.06195	125.099	51.90075	0.05559203	1.51428E-1	3.033907221
	Msa.1245.1.S1_at	39.04983	46.46942	39.77599	136.4275	160.9694	187.3533	41.76508	4.090226	161.5834	25.46844	0.001295651	0	3.868864345
	Msa.1277.1.S1_s_at	52.6183	73.69831	81.89249	93.19954	358.1177	174.8612	69.40304	15.10237	208.7262	135.667	0.15182713	1.80164E-57	3.007449977
	Msa.1328.1.S1_at	5.532806	5.337863	5.497426	6.867916	30.96503	11.45927	5.456032	0.103855	16.43074	12.7947	0.211563372	0	3.011481483
IMGA|Medtr5g030620.1	Msa.1569.1.S1_at	62.14862	58.69036	69.65314	255.8116	330.3875	378.9616	63.49737	5.604463	321.7202	62.03084	0.001991873	0	5.066670154
IMGA|Medtr5g030920.1	Msa.1643.1.S1_at	42.85484	31.22597	27.08367	118.2804	119.3477	128.6609	33.72149	8.176378	122.0963	5.710085	0.00010512	0	3.620726683
	Msa.1669.1.S1_at	381.1565	375.2438	373.865	1095.488	1076.958	976.3015	376.7551	3.873549	1049.583	64.13595	5.43391E-05	0	2.785848399
IMGA|Medtr2g034480.1	Msa.1736.1.S1_at	579.1983	395.865	772.0085	1847.987	2264.915	3122.01	582.3573	188.0917	2411.637	649.5609	0.009410617	1.14144E-63	4.141164981
	Msa.1879.1.S1_at	5.805465	6.626965	6.998846	13.89154	14.22012	11.20775	6.477092	0.610644	13.10647	1.652526	0.002861056	7.25068E-79	2.023511323
IMGA|Medtr5g097940.1	Msa.1879.1.S1_s_at	27.89693	33.08316	50.93498	506.6339	431.8067	464.3881	37.30502	12.08536	467.6096	37.51747	4.60721E-05	0	12.53476242
	Msa.1902.1.S1_at	82.03165	81.755	97.38014	202.5316	236.1983	187.2348	87.0556	8.942388	208.6549	25.04949	0.00137641	0	2.396800834
	Msa.2530.1.S1_at	10.54691	13.32155	18.15998	53.4155	41.86509	45.24048	14.00948	3.852875	46.84036	5.939081	0.001303653	0	3.343475712
	Msa.2530.1.S1_s_at	54.80817	58.75935	77.52223	257.1818	250.3011	196.5316	63.69658	12.13525	234.6715	33.20878	0.001111414	0	3.68420884
	Msa.2535.1.S1_at	648.767	470.3162	188.156	946.8577	879.2816	816.6983	435.7464	232.2432	880.9459	65.09567	0.032993482	0	2.021694112
	Msa.2671.1.S1_s_at	6663.115	6261.448	6104.078	21615.53	24889.7	23352.07	6342.881	288.2774	23285.77	1638.091	6.06122E-05	0	3.671166112
	Msa.2738.1.S1_at	119.4358	134.7102	73.93833	272.7216	405.0213	265.715	109.3614	31.61369	314.486	78.48412	0.013706768	2.64238E-29	2.875656598
	Msa.2767.1.S1_at	148.5804	127.4959	149.0092	356.9504	402.1983	311.5316	141.6952	12.2988	356.8935	45.33336	0.001365448	9.3959E-202	2.518741326
IMGA|Medtr3g088460.1	Msa.2831.1.S1_at	22.80206	20.73347	32.11425	65.60568	73.75257	98.64703	25.21659	6.062427	79.33509	17.21355	0.006808974	6.28389E-54	3.146146459
	Msa.2861.1.S1_at	29.29486	9.77835	13.94102	56.08615	80.63034	118.0626	17.67141	10.27913	84.92638	31.21079	0.023905305	9.0503E-30	4.805862984
IMGA|Medtr5g020800.1	Msa.2901.1.S1_s_at	37.16211	35.89199	61.89228	241.5907	169.8393	156.9507	44.98212	14.65839	189.4602	45.60395	0.006409539	2.41132E-65	4.211900217
IMGA|Medtr2g035100.1	Msa.2942.1.S1_s_at	337.5199	180.6281	281.7191	1750.351	1819.905	2243.666	266.6223	79.52795	1937.974	267.0112	0.000484428	0	7.26861028
	Msa.3005.1.S1_at	30.38031	40.09646	26.73987	182.728	255.54	273.7935	32.40554	6.904765	237.3538	48.17969	0.001878824	0	7.324482344
	Msa.3122.1.S1_at	5.836568	6.661197	7.297236	33.33134	34.5339	22.16835	6.598334	0.73236	30.01119	6.818667	0.004095193	0	4.548298987
	Msa.3124.1.S1_at	693.2278	669.4706	987.143	1871.493	1471.039	1582.701	783.2805	176.9493	1641.744	206.653	0.005450949	4.35255E-17	2.095985429
	Msa.3172.1.S1_at	47.5823	34.53202	48.55987	103.6175	112.9087	95.53442	43.55806	7.832047	104.0202	8.694141	0.000862291	0	2.388081925
	Msa.886.1.S1_s_at	1158.479	1394.421	2195.365	3609.409	3793.406	4367.118	1582.755	543.4938	3923.311	395.2052	0.003805791	0	2.478786244
	Msa.897.1.S1_s_at	12.48303	12.66205	20.27651	67.06511	188.8344	124.9297	15.14053	4.448788	126.9431	60.90963	0.033831334	0	8.38432329
	Msa.902.1.S1_at	13.22113	9.68027	12.22618	36.88133	67.22599	41.03182	11.70919	1.826165	48.37971	16.45276	0.018508572	4.8291E-265	4.131771239
	Msa.952.1.S1_at	32.163	53.39556	58.83807	142.6832	242.4483	149.2635	48.13221	14.09493	178.1317	55.79696	0.017356521	1.91028E-57	3.700882824
	Msa.982.1.S1_at	190.4689	251.2686	279.2579	546.32	806.571	638.6854	240.3318	45.39364	663.8588	131.9391	0.006265338	9.62479E-59	2.762259346
	Mtr.10096.1.S1_at	7.00715	7.22317	7.140877	28.46996	43.40126	35.18224	7.123732	0.109026	35.68449	7.478311	0.002708851	0	5.009240007
IMGA|Medtr6g036450.1	Mtr.10146.1.S1_at	17.35882	20.8871	23.36454	41.74367	42.21428	46.56759	20.53682	3.018145	43.50851	2.659667	0.000586441	0	2.118561626
	Mtr.1025.1.S1_at	6.147004	9.126064	8.155046	74.03116	57.08686	97.00771	7.809371	1.519315	76.04191	20.03624	0.00417642	0	9.737264041
IMGA|Medtr5g029190.1	Mtr.10253.1.S1_at	13.70826	16.35563	13.44251	26.46532	30.02507	37.02266	14.50213	1.610663	31.17102	5.37115	0.006750291	7.53636E-72	2.14940898
	Mtr.10272.1.S1_at	31.74475	32.71999	32.96428	149.1398	113.8621	125.4466	32.47634	0.645244	129.4828	17.98186	0.000732263	0	3.986988711
IMGA|Medtr8g077290.1	Mtr.10283.1.S1_at	22.21201	18.64213	28.6663	45.5425	55.88657	69.08103	23.17348	5.08078	56.8367	11.798	0.010504696	1.74431E-30	2.452661297
	Mtr.10302.1.S1_x_at	5.828572	6.147004	7.606522	23.87658	66.53165	41.37001	6.527366	0.948042	43.92608	21.4421	0.039238534	0	6.729526121
	Mtr.10308.1.S1_at	83.02882	102.8836	55.49517	216.9738	426.9091	292.6872	80.46919	23.79767	312.19	106.3178	0.021132105	8.11404E-64	3.879621952
IMGA|Medtr2g035190.1	Mtr.10311.1.S1_at	617.5316	649.701	662.2227	2292.098	2136.556	1949.365	643.1518	23.05411	2126.006	171.6097	0.000120274	0	3.305605784
IMGA|Medtr2g035220.1	Mtr.10317.1.S1_at	69.89532	82.19831	52.4874	403.6983	405.6655	380.3251	68.19368	14.92837	396.563	14.09675	1.01028E-05	0	5.815245704
IMGA|Medtr2g035210.1	Mtr.10319.1.S1_at	273.3281	153.865	220.74	3271.923	3536.11	2813.215	215.9777	59.87376	3207.083	365.7833	0.000151973	0	14.84914099
IMGA|Medtr8g074330.1	Mtr.10332.1.S1_at	95.51574	77.68012	63.97691	209.2636	126.1092	646.7162	79.05759	15.81447	327.363	279.6757	0.19950161	7.4051E-163	4.140816742
IMGA|Medtr2g029750.1	Mtr.10356.1.S1_at	78.84449	108.702	60.61201	434.7107	481.565	561.9033	82.71949	24.27803	492.7264	64.32664	0.000495809	0	5.956593052
IMGA|Medtr8g045490.1	Mtr.10361.1.S1_at	6.21158	6.037514	7.072166	26.12327	17.26252	20.45616	6.44042	0.553987	21.28065	4.487545	0.004727645	0	3.304233109

	Mtr.10368.1.S1_at	106.2865	98.24891	91.69831	713.7018	314.5129	265.0316	98.74457	7.306705	431.0821	246.0031	0.079475648	0	4.365628729
	Mtr.10382.1.S1_at	10.76431	9.941019	9.714197	10.49459	79.03165	11.75492	10.13984	0.552568	33.76039	39.21113	0.355722193	0	3.329478649
IMGA|Medtr2g035100.1	Mtr.10391.1.S1_at	7.069996	5.569564	6.19899	25.51238	22.6444	31.80257	6.279517	0.75345	26.65312	4.684438	0.001745192	0	4.244453368
	Mtr.10434.1.S1_at	11.77107	9.471738	10.18506	97.10706	157.9427	255.3056	10.47595	1.176943	170.1185	79.79902	0.025707639	0	16.23894589
	Mtr.10435.1.S1_s_at	4.817929	7.536633	5.753596	14.69557	14.55345	21.24035	6.036053	1.381186	16.82979	3.820316	0.010014901	9.65784E-42	2.788211037
	Mtr.10462.1.S1_at	18.0112	12.97775	16.99932	116.6565	366.4755	99.84376	15.99609	2.662475	194.3253	149.3233	0.107452591	0	12.14829723
	Mtr.10466.1.S1_at	36.69929	66.47832	110.1489	346.7826	250.9243	195.3734	71.10883	36.94309	264.3601	76.5936	0.01701464	1.29916E-19	3.717682491
IMGA|Medtr3g088110.1	Mtr.10488.1.S1_at	549.5941	574.4854	561.7531	1021.634	1170.301	1331.399	561.9442	12.44675	1174.444	154.9243	0.002408956	0	2.089966274
	Mtr.10502.1.S1_s_at	6.858476	9.310968	6.672904	40.68436	24.23186	20.24559	7.614116	1.472443	28.38727	10.8345	0.030197448	7.2095E-132	3.728242079
IMGA|Medtr4g122480.1	Mtr.10517.1.S1_at	265.4756	269.2553	361.8958	758.5063	805.0629	647.9593	298.8756	54.6098	737.1761	80.69458	0.001463708	0	2.46649852
IMGA|Medtr8g012550.1	Mtr.10572.1.S1_at	22.13736	22.80882	27.04205	57.6208	100.6432	263.865	23.99607	2.65917	140.7097	108.8033	0.13680833	0	5.863862325
	Mtr.10592.1.S1_at	155.584	194.6327	277.691	1096.91	855.9642	886.5099	209.3026	62.36132	946.4613	131.1842	0.000923815	0	4.521976767
IMGA|Medtr1g079810.1	Mtr.10683.1.S1_at	273.7863	334.865	338.0311	703.3747	906.1635	1127.463	315.5608	36.2124	912.3338	212.1116	0.008625469	3.3489E-179	2.891150767
IMGA|Medtr8g027180.1	Mtr.10700.1.S1_at	90.84837	137.2498	124.3425	227.8356	201.6143	295.1144	117.4802	23.94976	241.5215	48.22908	0.016267159	2.94687E-19	2.055847678
	Mtr.10743.1.S1_at	27.25805	13.54983	19.22512	68.93565	66.36812	84.3016	20.011	6.887819	73.20179	9.69806	0.001497111	0	3.658077591
IMGA|Medtr3g101900.1	Mtr.10760.1.S1_at	26.11349	31.30271	33.66601	144.9892	139.1983	149.3485	30.36074	3.863369	144.512	5.091874	6.50744E-06	0	4.759831365
IMGA|Medtr5g005170.2	Mtr.10838.1.S1_at	364.1588	487.2638	454.3804	942.0454	990.2918	962.5316	435.2677	63.7392	964.9563	24.21441	0.000176492	0	2.216926126
IMGA|Medtr4g082600.1	Mtr.10898.1.S1_at	42.67728	30.30891	38.23911	105.2531	82.19831	79.75746	37.0751	6.265803	89.06963	14.06835	0.004265509	7.66742E-47	2.402411166
	Mtr.10941.1.S1_at	48.23314	75.107	134.1624	271.7806	303.364	631.8112	85.83417	43.9575	402.3186	199.3728	0.054944018	1.08294E-35	4.687162049
IMGA|Medtr5g021060.1	Mtr.10969.1.S1_at	32.77055	69.9161	18.86448	98.71687	125.0316	117.3113	40.51704	26.39267	113.6866	13.52666	0.012916307	0	2.805896242
IMGA|Medtr7g065260.2	Mtr.11080.1.S1_s_at	30.206	27.56827	26.23883	78.08316	85.45518	90.79904	28.00437	2.019218	84.77913	6.384841	0.000125134	0	3.027353877
IMGA|Medtr7g114020.1	Mtr.11120.1.S1_at	22.35509	28.12631	17.02424	46.01694	50.10124	47.01166	22.50188	5.552492	47.70994	2.129807	0.001832472	0	2.120264794
IMGA|Medtr4g061610.1	Mtr.11257.1.S1_at	8.755139	7.72646	5.39893	13.36129	15.29109	19.94683	7.29351	1.719482	16.19974	3.385493	0.015316477	2.93229E-19	2.221116546
IMGA|Medtr7g037040.1	Mtr.11264.1.S1_at	72.12523	79.05986	83.10453	170.865	151.0766	168.4009	78.09654	5.552676	163.4475	10.7841	0.000260152	0	2.092890171
IMGA|Medtr3g069050.1	Mtr.11267.1.S1_at	8.828637	11.23276	10.25998	24.83673	29.55141	38.47721	10.10712	1.209328	30.95512	6.927732	0.006816231	6.7638E-196	3.062702699
IMGA|Medtr2g084010.1	Mtr.11354.1.S1_at	45.66791	55.50314	81.25988	365.0777	527.8723	565.6359	60.81031	18.37993	486.1953	106.5768	0.002426304	0	7.995277725
	Mtr.11532.1.S1_at	9.424871	6.801641	7.338471	19.4024	25.19065	39.43831	7.854994	1.385796	28.01045	10.3113	0.028424211	5.0184E-140	3.565941507
IMGA|Medtr7g111240.1	Mtr.1156.1.S1_at	283.1809	245.5275	282.3954	996.9524	1022.212	1259.696	270.3679	21.51604	1092.954	144.9546	0.000626637	0	4.042467529
	Mtr.11654.1.S1_at	26.10356	27.57364	32.64572	62.55713	62.2188	51.41324	28.77431	3.43237	58.72972	6.338515	0.001974229	1.26775E-51	2.041047289
	Mtr.11689.1.S1_at	33.21607	20.23205	44.41082	150.0494	176.1527	125.0316	32.61964	12.10042	150.4113	25.56247	0.001957918	8.7606E-64	4.611063901
IMGA|Medtr4g094240.1	Mtr.11811.1.S1_s_at	999.0316	809.9558	1012.532	2241.556	2069.655	2434.071	940.5064	113.2614	2248.427	182.3054	0.000455768	0	2.390656301
IMGA|Medtr2g084080.1	Mtr.12006.1.S1_at	10.89957	22.48727	14.78698	50.24557	313.6665	181.2857	16.05794	5.897471	181.7326	131.711	0.095115478	0	11.31730467
IMGA|Medtr2g084080.1	Mtr.12025.1.S1_at	10.43691	8.517623	8.171712	25.92695	146.72	91.90172	9.042081	1.220274	88.1829	60.48235	0.08611518	0	9.752500365
IMGA|Medtr8g106150.1	Mtr.12088.1.S1_at	399.4377	494.1191	534.5189	950.4093	1006.84	940.1156	476.0253	69.33452	965.7883	35.92255	0.000407524	0	2.028859388
IMGA|Medtr8g103250.1	Mtr.12141.1.S1_at	21.20955	20.46242	28.00017	106.4947	101.128	290.5719	23.22405	4.153078	166.0649	107.8597	0.083663967	0	7.150557979
IMGA|Medtr2g098540.2	Mtr.12161.1.S1_at	7.078533	9.334525	8.821642	13.97928	24.88422	26.80883	8.411567	1.18258	21.89078	6.918804	0.029210515	9.45564E-87	2.602461373
	Mtr.12193.1.S1_at	6.48975	5.36137	6.606853	21.84452	14.91763	12.39792	6.152658	0.687772	16.38669	4.891645	0.022994473	1.8693E-146	2.663351767
	Mtr.12246.1.S1_at	7.240464	10.58614	7.952506	170.2174	521.2115	310.7464	8.593038	1.762414	334.0584	176.6545	0.033186295	0	38.87547241
IMGA|Medtr1g106870.1	Mtr.12311.1.S1_at	8.881766	10.63183	9.943245	86.59911	183.6983	170.784	9.818948	0.88163	147.0271	52.72907	0.010769656	0	14.97381661
	Mtr.12423.1.S1_at	87.36441	109.9057	85.91643	348.2481	269.9701	320.8221	94.39552	13.45173	313.0134	39.71892	0.00083324	0	3.315977441
IMGA|Medtr7g093850.1	Mtr.12424.1.S1_at	97.84434	200.862	266.0111	449.3065	368.7249	343.1647	188.2392	84.79105	387.0654	55.39675	0.027273946	5.0818E-10	2.056242412
IMGA|Medtr4g100380.1	Mtr.12456.1.S1_at	227.0379	368.7752	385.0851	604.2137	691.0799	749.9314	326.9661	86.92371	681.7417	73.3063	0.005676718	0	2.085053448
	Mtr.12474.1.S1_at	5.076418	7.919811	5.876794	20.10334	36.19537	44.7082	6.291008	1.466254	33.66897	12.49547	0.019619439	1.8901E-229	5.351920145
	Mtr.12486.1.S1_at	29.3112	33.3343	27.64597	169.255	362.5316	195.2241	30.09716	2.924479	242.3369	104.8984	0.024826912	0	8.051820454
	Mtr.12502.1.S1_at	137.7681	142.8438	230.6531	376.6983	515.865	501.7708	170.4217	52.22366	464.778	76.60411	0.005330972	1.62879E-22	2.727223739
IMGA|Medtr5g090920.1	Mtr.12513.1.S1_at	11.6676	20.60827	18.89251	61.58197	96.44181	77.14666	17.05613	4.744797	78.39014	17.46316	0.004205249	4.9851E-111	4.596010935
IMGA|Medtr7g005280.1	Mtr.12514.1.S1_at	420.8405	433.9953	593.6983	1128.782	959.2816	1067.293	482.8447	96.22712	1051.786	85.80753	0.001574105	0	2.178310369
IMGA|Medtr1g099310.1	Mtr.12525.1.S1_at	75.7384	73.1747	70.93327	389.5185	136.5029	188.9667	73.28212	2.404363	238.3294	133.5355	0.099039528	0	3.252216865
IMGA|Medtr2g011080.1	Mtr.12528.1.S1_at	12.33336	10.93941	12.36594	29.75135	25.49025	24.17864	11.87957	0.814363	26.47342	2.913544	0.001121712	0	2.228482681
	Mtr.12566.1.S1_at	5.285027	5.748574	5.836942	61.75386	7.082153	10.77641	5.623514	0.29645	26.53747	30.55417	0.301429335	0	4.719019417

	Mtr.12569.1.S1_at	83.49227	94.00783	51.69831	491.1019	775.525	1035.865	76.39947	22.0285	767.4973	272.4702	0.011884991	0	10.04584559
IMGA|Medtr1g018640.1	Mtr.12626.1.S1_at	2964.508	2208.152	3774.947	7355.597	5859.741	6927.263	2982.536	783.5534	6714.2	770.3523	0.004174773	0	2.25117194
IMGA|Medtr4g126970.1	Mtr.12664.1.S1_at	13.22113	13.24716	15.0342	60.4568	30.37514	36.44038	13.83416	1.039345	42.42411	15.9085	0.036006508	0	3.06661902
	Mtr.12668.1.S1_at	137.9262	144.365	124.1983	342.3196	262.3233	350.0138	135.4965	10.30055	318.2189	48.55963	0.003104565	2.6383E-207	2.348539516
	Mtr.12685.1.S1_at	26.2383	27.75687	32.28442	63.81564	50.69832	60.23415	28.75986	3.145377	58.24937	6.780158	0.002398367	2.68109E-59	2.025370185
	Mtr.12696.1.S1_at	9.8359	8.843747	5.694105	16.9408	20.85228	30.48935	8.124584	2.162525	22.76081	6.972997	0.02552787	9.75762E-32	2.801473815
IMGA|Medtr8g091390.1	Mtr.12710.1.S1_at	57.60323	59.46605	74.52595	216.9301	172.4594	243.7755	63.86508	9.27945	211.055	36.01924	0.002371939	3.6258E-166	3.304701474
	Mtr.12748.1.S1_at	11.00271	13.02511	12.49683	20.86728	74.40402	85.03144	12.17488	1.048933	60.10091	34.39032	0.073344202	0	4.936467098
IMGA|AC158498_5.1	Mtr.12925.1.S1_at	21.63082	17.25539	15.069	51.55101	41.41699	63.87802	17.98507	3.341208	52.282	11.24834	0.007167888	1.02511E-70	2.906966927
	Mtr.13033.1.S1_at	542.7295	378.5206	142.8922	932.9683	827.7067	726.476	354.7141	200.9789	829.0503	103.2527	0.022040923	1.77636E-15	2.337235323
	Mtr.13058.1.S1_at	41.60201	38.69873	47.60101	159.4787	137.6816	181.742	42.63392	4.539961	159.6341	22.03059	0.000840499	0	3.744298639
	Mtr.13186.1.S1_at	16.80994	11.50916	17.23431	36.63725	38.02428	36.11734	15.18447	3.189976	36.92629	0.985779	0.000352106	0	2.431845491
IMGA|Medtr6g081810.1	Mtr.13237.1.S1_at	52.86498	50.16306	46.19833	113.547	122.3194	101.5394	49.74212	3.3532	112.4686	10.43192	0.000580865	0	2.261033503
IMGA|Medtr8g095790.1	Mtr.13471.1.S1_at	8.658785	9.192508	7.620994	17.96815	19.32686	26.2383	8.490762	0.799117	21.17777	4.434889	0.008181863	1.8997E-166	2.494212671
IMGA|Medtr5g016440.1	Mtr.13532.1.S1_at	11.36088	11.6107	13.6995	80.77754	38.16917	25.91012	12.22369	1.284176	48.28561	28.7987	0.096161306	0	3.950164686
IMGA|Medtr3g033810.1	Mtr.13584.1.S1_at	8.672115	8.839309	8.391123	36.07872	34.77371	57.15473	8.634182	0.226488	42.66905	12.56193	0.009363833	0	4.941875175
	Mtr.13989.1.S1_at	7.92433	21.4371	30.28533	178.5838	107.6322	275.0501	19.88225	11.26129	187.0887	84.0324	0.026881879	7.4889E-146	9.409832758
IMGA|Medtr8g038210.1	Mtr.14183.1.S1_at	29.0649	33.8862	15.62	62.3651	82.81686	80.95423	26.19036	9.466295	75.37873	11.30855	0.004458843	2.25704E-19	2.878109257
IMGA|Medtr8g079550.1	Mtr.14263.1.S1_at	65.69831	74.4575	101.1127	181.1547	125.4082	178.7934	80.42284	18.44543	161.7854	31.52574	0.018174337	2.17124E-14	2.011685142
IMGA|Medtr4g112460.1	Mtr.14271.1.S1_at	12.66522	14.12224	14.01728	35.70156	40.17394	45.40412	13.60158	0.81261	40.42654	4.856212	0.000703226	0	2.972194575
IMGA|Medtr3g014180.1	Mtr.14281.1.S1_at	212.9429	273.0876	225.4235	863.4488	771.0316	596.0316	237.1513	31.74118	743.504	135.8172	0.003267456	4.7709E-168	3.135146083
IMGA|Medtr8g091660.1	Mtr.14283.1.S1_at	1045.326	937.768	1025.775	2765.625	2297.823	3208.448	1002.956	57.29486	2757.299	455.3699	0.002699138	0	2.7491711
IMGA|Medtr4g062080.1	Mtr.14303.1.S1_at	50.41591	52.62534	31.47798	105.4412	93.9157	104.5704	44.83974	11.62424	101.3091	6.417667	0.001809863	0	2.259359701
	Mtr.14757.1.S1_at	5.109534	4.852313	4.432092	7.434876	21.61933	16.00565	4.79798	0.341974	15.01995	7.143417	0.068528734	0	3.130474639
IMGA|Medtr2g093760.1	Mtr.1493.1.S1_at	72.10979	68.56597	103.7928	441.0848	451.2491	794.0316	81.48952	19.39632	562.1219	200.904	0.014559069	0	6.898087736
IMGA|Medtr1g086450.1	Mtr.14994.1.S1_at	11.0076	9.311398	15.45214	124.5606	7.77715	32.53648	11.92371	3.171215	54.95806	61.53566	0.29300101	3.6792E-122	4.609140498
IMGA|Medtr1g086510.1	Mtr.15010.1.S1_s_at	7.010345	5.501826	6.037856	24.22159	10.35539	40.3819	6.183342	0.76471	24.98629	15.02785	0.096417723	0	4.04090393
IMGA|Medtr4g087510.1	Mtr.15070.1.S1_s_at	7.847143	8.939342	10.26677	44.61449	35.30177	61.90121	9.01775	1.211715	47.27249	13.49746	0.008105848	0	5.242160258
	Mtr.1509.1.S1_at	39.18284	55.65704	44.84012	203.2178	233.7436	173.6122	46.56	8.370679	203.5245	30.0669	0.000956475	0	4.371231313
	Mtr.15261.1.S1_s_at	70.03165	95.23482	66.66145	262.6656	434.0604	519.1781	77.30931	15.61514	405.3014	130.6521	0.012472441	8.4145E-290	5.242595088
IMGA|Medtr1g043080.1	Mtr.15332.1.S1_at	26.86633	40.46691	41.77962	90.69831	57.95361	94.11359	36.37096	8.257374	80.92184	19.96423	0.02334063	9.1974E-21	2.224902737
IMGA|Medtr1g017830.1	Mtr.15537.1.S1_at	11.14126	9.223665	15.84085	46.47045	107.6983	124.8799	12.06859	3.404666	93.01622	41.21509	0.027523645	0	7.707297145
IMGA|Medtr5g097940.1	Mtr.15678.1.S1_s_at	48.53131	56.22541	94.48772	1015.82	811.0316	957.0776	66.41482	24.61434	927.9763	105.4499	0.000160671	0	13.97242886
IMGA|Medtr5g037710.1	Mtr.15831.1.S1_s_at	32.38488	41.57588	81.23993	170.5961	177.8381	283.6548	51.73356	25.96321	210.6963	63.28755	0.015799436	2.83418E-26	4.072720605
	Mtr.1587.1.S1_at	9.38672	9.225548	15.37992	88.84869	88.24935	89.53236	11.33073	3.507625	88.8768	0.641963	2.96689E-06	0	7.843873781
IMGA|Medtr1g008220.1	Mtr.15921.1.S1_at	108.3112	142.9219	185.0059	323.443	343.5528	381.6542	145.413	38.40801	349.55	29.56538	0.001877387	0	2.403843246
IMGA|Medtr4g086620.1	Mtr.16207.1.S1_at	109.178	82.09145	124.3233	326.1399	353.1188	357.1996	105.1976	21.39546	345.4861	16.87811	0.000107206	0	3.284163436
IMGA|Medtr7g077160.1	Mtr.16278.1.S1_at	14.61252	25.64133	53.74831	209.2002	299.0839	537.365	31.33405	20.17939	348.5497	169.5824	0.032365764	3.0733E-163	11.12367111
	Mtr.16773.1.S1_x_at	23.64417	13.01508	20.97148	27.77542	183.6929	38.91199	19.21024	5.529091	83.46011	86.98257	0.270755482	4.28307E-90	4.344562933
IMGA|Medtr4g127930.1	Mtr.16818.1.S1_s_at	27.68789	45.68433	52.46636	115.7908	108.0878	141.2633	41.94619	12.80521	121.714	17.36276	0.003053735	3.85896E-27	2.901668734
IMGA|Medtr2g035850.1	Mtr.16822.1.S1_at	49.21243	26.69318	82.51571	177.1819	119.8956	149.4089	52.80711	28.08434	148.8288	28.64756	0.014310297	3.18117E-09	2.81834812
IMGA|Medtr5g089250.1	Mtr.17039.1.S1_s_at	10.99841	22.00212	11.07449	72.918	60.81806	57.185	14.69167	6.33115	63.64036	8.237463	0.001227524	0	4.331729417
IMGA|Medtr3g088630.1	Mtr.17273.1.S1_s_at	71.17587	66.16062	83.23108	183.3527	184.6709	201.3736	73.52252	8.773837	189.799	10.04549	0.000112114	0	2.581508909
IMGA|Medtr3g088460.1	Mtr.17288.1.S1_at	60.07634	43.05808	70.30205	222.2865	274.9586	359.723	57.81216	13.76239	285.656	69.33991	0.005049194	7.8147E-181	4.941106175
IMGA|Medtr5g097940.1	Mtr.17404.1.S1_s_at	36.72334	37.45656	71.72121	776.9338	538.5603	651.6595	48.6337	19.99773	655.7179	119.2385	0.000962349	0	13.48278772
IMGA|Medtr7g092540.1	Mtr.17497.1.S1_at	30.66127	25.48048	56.43935	72.40503	82.19831	94.70362	37.52703	16.58212	83.10232	11.17675	0.016853382	1.63225E-12	2.214465624
IMGA|Medtr3g095420.1	Mtr.17526.1.S1_at	33.01979	17.95419	22.19661	351.0064	448.7582	396.0551	24.3902	7.768652	398.6066	48.92583	0.000197003	0	16.34290031
IMGA|Medtr3g095340.1	Mtr.17531.1.S1_at	40.53159	34.70688	39.46562	98.52236	56.78664	83.86628	38.2347	3.10132	79.72509	21.1738	0.028353439	8.7613E-119	2.085150515
IMGA|Medtr7g016800.1	Mtr.17621.1.S1_x_at	75.06488	51.79686	75.55214	407.4751	214.9484	279.8451	67.47129	13.57664	300.7562	97.95197	0.015023881	1.2309E-194	4.45754346
IMGA|Medtr1g014240.1	Mtr.17910.1.S1_at	28.38516	20.95624	33.82984	94.82768	119.0584	166.1693	27.72375	6.462237	126.6851	36.27713	0.00964932	5.0909E-155	4.56955283

IMGA|Medtr5g074290.1	Mtr.18.1.S1_at	175.1648	158.5985	222.5333	322.688	339.5472	451.8471	185.4322	33.18102	371.3608	70.21107	0.014295777	2.85731E-22	2.0026765
IMGA|Medtr4g059730.1	Mtr.18369.1.S1_at	18.07694	25.3401	22.6444	47.71824	36.69929	50.23736	22.02048	3.671556	44.88496	7.200028	0.008043963	3.99732E-27	2.038328169
IMGA|Medtr5g014100.1	Mtr.18570.1.S1_at	8.97505	7.604541	8.878652	14.17464	25.25278	15.47759	8.486081	0.764956	18.30167	6.054988	0.049532035	2.0262E-109	2.156669296
IMGA|Medtr3g098390.1	Mtr.18629.1.S1_at	64.16479	91.82816	134.0092	325.9427	241.3347	347.3085	96.66737	35.17276	304.862	56.04382	0.005506757	1.15494E-24	3.153721458
IMGA|Medtr2g034480.1	Mtr.18650.1.S1_at	343.4249	230.8756	459.843	1152.809	1385.221	2062.768	344.7145	114.4892	1533.599	472.7774	0.013335842	2.50541E-72	4.448897423
IMGA|Medtr3g116440.1	Mtr.1872.1.S1_at	20.23245	25.17798	26.36489	328.7218	344.218	490.135	23.9251	3.252532	387.6916	89.05631	0.002111664	0	16.20438661
	Mtr.1887.1.S1_at	48.82566	35.48447	47.11124	90.34688	91.86498	94.92009	43.80712	7.258426	92.37732	2.329257	0.00038329	0	2.108728182
IMGA|Medtr8g102150.1	Mtr.19134.1.S1_at	289.846	389.7435	190.3245	711.2746	1180.698	784.7276	289.9713	99.70956	892.2335	252.5031	0.018420265	1.29275E-25	3.076971744
IMGA|AC233577_9.1	Mtr.1954.1.S1_at	19.17137	24.26972	24.7662	39.5855	55.86498	46.73617	22.73576	3.096818	47.39555	8.159744	0.008079477	2.8407E-43	2.084625289
IMGA|Medtr1g043350.1	Mtr.19570.1.S1_at	11.18492	10.40329	14.83832	108.7282	21.2032	67.51723	12.14218	2.367414	65.81621	43.78729	0.101338088	0	5.420462378
IMGA|Medtr1g043320.1	Mtr.19575.1.S1_at	11.7955	17.66616	10.63816	48.8558	119.1226	159.5032	13.36661	3.768219	109.1605	55.99235	0.041696089	0	8.166660258
IMGA|Medtr1g083580.1	Mtr.19594.1.S1_at	24.30692	26.75732	32.78857	56.60485	41.90793	76.2744	27.95094	4.364992	58.26239	17.24309	0.041900575	2.54092E-33	2.084452209
IMGA|Medtr8g077810.1	Mtr.19623.1.S1_at	9.040911	7.9761	7.264361	19.33446	15.83804	17.95419	8.093791	0.894103	17.70889	1.76107	0.001083267	0	2.187960425
IMGA|Medtr2g020710.1	Mtr.19796.1.S1_at	20.32088	34.6355	31.22652	88.03165	63.63745	131.0316	28.72763	7.477325	94.23358	34.12246	0.031435015	5.27191E-52	3.280241806
IMGA|Medtr8g078270.1	Mtr.19871.1.S1_at	62.1447	89.40866	126.1428	219.7519	166.8702	216.2755	92.56539	32.11562	200.9659	29.57882	0.012643086	2.18668E-10	2.171069375
IMGA|Medtr4g076430.1	Mtr.19888.1.S1_at	10.65816	15.4709	20.6229	140.5889	300.8239	79.79522	15.58399	4.983336	173.736	114.1817	0.074617744	0	11.14836767
IMGA|Medtr4g083130.1	Mtr.19978.1.S1_at	22.44814	29.37937	34.99502	70.09622	57.06326	60.10689	28.94084	6.284927	62.42212	6.81797	0.003333659	0	2.156886814
IMGA|Medtr4g083000.2	Mtr.19990.1.S1_at	436.7679	370.1983	289.1804	801.0316	621.646	976.181	365.3822	73.91157	799.6196	177.2718	0.01730578	2.53884E-24	2.188446906
	Mtr.20002.1.S1_s_at	262.5976	270.8651	286.7624	696.0694	613.0445	701.5261	273.4084	12.28147	670.2133	49.58476	0.000176556	0	2.45132704
IMGA|Medtr4g086320.1	Mtr.20215.1.S1_s_at	18.55114	16.37684	18.86448	103.9929	59.19831	81.83816	17.93082	1.354873	81.67647	22.39774	0.007926266	0	4.555089135
IMGA|Medtr8g038100.1	Mtr.20386.1.S1_s_at	2140.577	1054.834	1364.532	6224.389	4419.222	3627.964	1519.981	559.3147	4757.192	1330.798	0.017778338	1.1861E-23	3.129770393
IMGA|Medtr6g012640.1	Mtr.20391.1.S1_at	238.6012	132.8786	328.5076	535.1187	577.1012	785.5851	233.3291	97.92101	632.6017	134.1402	0.014098832	1.63614E-12	2.711199184
IMGA|Medtr7g016700.1	Mtr.20464.1.S1_x_at	50.66746	60.64609	63.89711	245.2565	146.1927	137.9676	58.40355	6.894026	176.4723	59.7107	0.027220524	2.2716E-193	3.021602076
IMGA|Medtr5g043070.1	Mtr.20555.1.S1_s_at	8.114542	7.900799	8.606409	15.18654	14.19688	21.84452	8.20725	0.361825	17.07598	4.159218	0.021215713	0	2.080597399
IMGA|Medtr8g076520.1	Mtr.20735.1.S1_at	39.66425	39.86525	70.2459	121.0712	79.92075	104.865	49.92513	17.59859	101.9523	20.72928	0.0295448	3.04712E-07	2.042104085
IMGA|Medtr3g079340.1	Mtr.20784.1.S1_s_at	87.41005	107.7599	90.50291	516.865	143.6004	207.0651	95.22429	10.96575	289.1768	199.7207	0.16835407	4.1903E-206	3.036797089
IMGA|Medtr4g100600.3	Mtr.20884.1.S1_at	644.5186	671.4772	865.213	1528.239	1645.95	1533.117	727.0696	120.3926	1569.102	66.59682	0.000448266	0	2.158118345
	Mtr.2090.1.S1_at	22.27874	29.18546	19.54126	47.46854	44.82757	70.53159	23.66849	4.970026	54.2759	14.13963	0.024075161	1.4579E-26	2.293171547
IMGA|Medtr1g083950.1	Mtr.21000.1.S1_at	125.7714	109.6581	137.0368	270.5529	311.9311	295.3143	124.1554	13.76067	292.5994	20.82228	0.000306238	0	2.356719066
	Mtr.2103.1.S1_at	87.36498	78.54954	69.62002	156.2578	187.9459	184.865	78.51152	8.872541	176.3562	17.47382	0.00098354	0	2.246246681
IMGA|Medtr7g024760.1	Mtr.2105.1.S1_at	49.16145	54.73191	47.58668	235.9945	182.7097	252.5547	50.49335	3.754205	223.753	36.49621	0.001216663	0	4.43133605
IMGA|Medtr2g037790.1	Mtr.21098.1.S1_at	29.26072	15.18569	18.76619	106.4508	77.56055	125.3039	21.07087	7.315069	103.1051	24.04686	0.004824591	4.84445E-84	4.893253035
	Mtr.21324.1.S1_at	55.84232	44.93395	80.95496	151.3243	108.2559	195.5192	60.57707	18.47138	153.6998	43.74443	0.027358767	2.50062E-18	2.537260256
IMGA|Medtr4g118800.2	Mtr.21662.1.S1_at	82.72042	106.2751	59.94661	277.4533	243.7474	360.0815	82.98071	23.16534	293.7607	59.85696	0.004717366	5.87947E-56	3.540108775
IMGA|Medtr6g012830.1	Mtr.21790.1.S1_at	17.87958	18.8044	29.0271	90.57148	47.31522	118.8758	21.90369	6.186357	85.58751	36.03971	0.039298391	4.12489E-71	3.907446942
IMGA|Medtr6g012810.1	Mtr.21791.1.S1_at	15.60048	17.185	20.92459	92.06346	68.69831	121.3472	17.90335	2.733783	94.03631	26.37981	0.007639947	0	5.252440921
IMGA|Medtr5g032910.1	Mtr.21812.1.S1_at	15.96549	10.00605	14.22455	32.17869	18.97705	36.02755	13.39869	3.064353	29.0611	8.942563	0.045478798	8.53795E-19	2.16895026
IMGA|Medtr5g032790.1	Mtr.21822.1.S1_at	120.5277	75.07469	89.51394	191.0131	249.3349	238.8833	95.03878	23.22472	226.4104	31.09723	0.004225968	1.15498E-22	2.382295086
IMGA|Medtr7g050870.1	Mtr.21871.1.S1_at	273.1514	342.9153	368.4972	725.7268	908.1153	979.2307	328.188	49.34954	871.0243	130.7588	0.002543135	6.29327E-81	2.654040839
IMGA|Medtr8g032300.1	Mtr.22068.1.S1_at	31.61996	45.25574	41.58471	80.30094	78.22128	91.69681	39.4868	7.055815	83.40634	7.25466	0.001676571	0	2.112258687
	Mtr.2238.1.S1_s_at	5229.698	4861.865	4596.282	14340.86	19398.53	16161.7	4895.948	318.0808	16633.7	2561.657	0.00140492	0	3.397441568
IMGA|Medtr6g071400.1	Mtr.22423.1.S1_at	12.19176	11.59599	9.506168	85.05679	16.82927	16.57553	11.09797	1.410359	39.4872	39.46463	0.281032068	2.6107E-266	3.558055792
IMGA|Medtr4g127560.1	Mtr.22444.1.S1_at	10.62635	15.50348	11.41413	79.01202	28.10908	20.61503	12.51465	2.618201	42.57871	31.77388	0.177741654	5.1128E-88	3.402308952
IMGA|Medtr2g008030.1	Mtr.22592.1.S1_at	33.70154	29.37937	37.43909	88.54389	45.1493	175.0368	33.50667	4.033392	102.91	66.12474	0.143783636	3.5021E-195	3.071329298
IMGA|Medtr2g045280.1	Mtr.22656.1.S1_s_at	24.49372	16.94226	21.48941	77.76202	72.99677	145.7358	20.97513	3.801908	98.83155	40.69014	0.029941	1.4632E-275	4.711844292
IMGA|Medtr4g016920.1	Mtr.2288.1.S1_at	15.48197	14.57123	17.37074	56.08788	52.14284	78.03808	15.80798	1.427945	62.0896	13.95193	0.004635078	0	3.927737597
IMGA|Medtr7g021010.1	Mtr.23418.1.S1_s_at	170.6168	187.1983	205.3781	2201.086	2128.397	2895.764	187.7311	17.38681	2408.415	423.618	0.000818345	0	12.82907247
IMGA|Medtr3g034610.1	Mtr.23565.1.S1_at	3.620191	6.988346	12.97858	9.88213	192.3851	144.2499	7.862371	4.74002	115.5057	94.58596	0.120350539	0	14.6909496
IMGA|Medtr7g023650.1	Mtr.23575.1.S1_x_at	165.1563	158.3588	235.5492	378.8325	495.9383	622.9182	186.3547	42.73901	499.2297	122.0761	0.013808695	7.66838E-37	2.678921243
IMGA|Medtr7g023650.1	Mtr.23578.1.S1_s_at	196.8419	119.7359	222.2719	1362.271	2784.367	3498.152	179.6166	53.39423	2548.263	1087.339	0.019629257	0	14.18724008

IMGA|Medtr7g023740.1	Mtr.23580.1.S1_at	32.43681	41.61961	57.0672	125.061	194.7081	157.7816	43.70788	12.44727	159.1836	34.84469	0.005671411	4.2382E-58	3.641988829
IMGA|Medtr3g009020.1	Mtr.23863.1.S1_at	85.6111	83.35712	160.0593	234.2948	249.5806	317.1223	109.6759	43.64793	266.9992	44.07556	0.011756341	4.29382E-10	2.434439363
IMGA|Medtr7g071970.1	Mtr.23965.1.S1_s_at	1217.905	808.0316	979.1312	3010.079	2883.656	3084.853	1001.689	205.8656	2992.863	101.6973	0.000114484	0	2.987815557
IMGA|Medtr1g102240.1	Mtr.24211.1.S1_at	81.47401	133.5737	147.1744	1233.059	220.6983	469.865	120.7407	34.67925	641.2075	527.482	0.163331008	5.6932E-149	5.310615353
IMGA|Medtr4g070590.1	Mtr.24242.1.S1_at	106.865	116.5082	149.6983	291.0752	267.6389	353.9651	124.3572	22.4695	304.2264	44.64043	0.003373512	1.0303E-43	2.446392187
IMGA|Medtr1g019440.1	Mtr.24415.1.S1_at	17.34119	17.57769	17.61708	72.41323	149.6983	156.6917	17.51198	0.149221	126.2677	46.77026	0.015765519	0	7.210361796
IMGA|Medtr1g019440.1	Mtr.24415.1.S1_s_at	53.61852	38.7358	42.47181	196.1699	407.048	385.3766	44.94204	7.742763	329.5315	116.0017	0.013265212	0	7.332366327
IMGA|Medtr8g023060.1	Mtr.24476.1.S1_at	96.67045	106.9801	97.37161	179.4238	376.1642	273.6098	100.3407	5.760522	276.3993	98.39985	0.036442601	0	2.754607804
IMGA|Medtr8g023060.1	Mtr.24476.1.S1_s_at	61.30268	75.31902	75.16051	143.5799	291.8189	214.8641	70.59407	8.046975	216.7543	74.13757	0.027409552	3.0759E-217	3.070432091
IMGA|Medtr8g088860.1	Mtr.24519.1.S1_at	271.4808	386.255	410.4273	1808.432	2326.035	2158.68	356.0544	74.23333	2097.716	264.1321	0.00038886	0	5.891560099
IMGA|Medtr8g093990.1	Mtr.24708.1.S1_at	5.877876	5.258006	5.340203	9.283153	10.63479	14.04084	5.492028	0.336672	11.31959	2.451655	0.015113381	2.2669E-197	2.06109546
IMGA|Medtr3g052760.1	Mtr.2494.1.S1_s_at	11.43835	10.46823	4.802918	20.18113	55.44071	20.96949	8.903165	3.583895	32.19711	20.13341	0.119766465	2.12376E-29	3.616366554
IMGA|Medtr7g074710.1	Mtr.25021.1.S1_at	94.66341	104.5754	123.9832	469.5859	534.8533	436.8789	107.7407	14.91398	480.4394	49.8808	0.00024321	0	4.459220926
IMGA|Medtr5g018750.1	Mtr.25125.1.S1_at	48.40404	55.45817	39.19532	112.2858	97.24869	145.1665	47.68584	8.155175	118.2337	24.50634	0.009096907	9.42512E-51	2.479429045
IMGA|Medtr5g006780.1	Mtr.25362.1.S1_s_at	26.07186	24.96387	29.78813	172.7243	254.602	220.5932	26.94129	2.526916	215.9732	41.13391	0.001359274	0	8.016439142
IMGA|Medtr1g102370.1	Mtr.25887.1.S1_at	23.29658	33.10321	31.10475	54.86498	70.76669	94.93942	29.16818	5.182206	73.5237	20.17897	0.021063562	1.01063E-49	2.52068186
IMGA|Medtr6g092620.1	Mtr.25950.1.S1_at	6.663752	5.946856	6.209657	13.82431	70.80042	16.57951	6.273422	0.362677	33.73475	32.12936	0.212896862	0	5.377407863
IMGA|Medtr7g072270.1	Mtr.25969.1.S1_at	12.48303	19.65177	19.65177	65.76527	36.3661	84.80472	17.26219	4.138873	62.31203	24.40325	0.034432861	2.79826E-79	3.609740963
	Mtr.2604.1.S1_x_at	351.6141	292.9597	300.2968	2142.372	4347.032	2511.641	314.9568	31.95739	3000.348	1180.787	0.016993227	0	9.526220069
IMGA|Medtr4g078660.1	Mtr.26151.1.S1_s_at	29.9511	33.87024	32.51694	172.9426	85.334	102.1594	32.11276	1.990585	120.1454	46.49128	0.030595949	0	3.741358576
IMGA|Medtr5g078220.1	Mtr.26370.1.S1_at	88.00557	156.9497	69.56126	430.5661	520.6211	431.4067	104.8389	46.06198	460.8646	51.75232	0.000880594	0	4.395933575
	Mtr.26884.1.S1_at	69.97499	100.4485	112.0316	175.0316	218.9386	191.4886	94.1517	21.7239	195.1529	22.18164	0.004882117	8.08884E-16	2.072750026
	Mtr.26895.1.S1_s_at	7.761813	6.687713	7.642456	53.30785	7.789143	8.001094	7.363994	0.588709	23.0327	26.21927	0.359212034	0	3.12774517
	Mtr.26988.1.S1_at	16.09978	25.15141	51.00382	61.34499	64.1209	65.43346	30.75167	18.1134	63.63312	2.087427	0.035405428	0	2.069257044
	Mtr.2742.1.S1_at	90.93926	92.19831	115.5034	208.2134	183.6983	218.775	99.54698	13.83297	203.5622	17.99495	0.001363983	0	2.044885941
IMGA|Medtr3g085020.1	Mtr.27693.1.S1_at	25.37992	25.32397	13.64865	96.53165	47.29295	107.075	21.45084	6.75696	83.63319	31.91003	0.029877615	3.36767E-57	3.898829606
IMGA|Medtr5g089180.1	Mtr.27718.1.S1_at	13.58065	12.59177	5.418434	8.587357	94.81159	7.531337	10.53028	4.454517	36.97676	50.08921	0.413873363	8.39192E-25	3.511468827
IMGA|Medtr7g100240.1	Mtr.27895.1.S1_at	112.5116	99.84329	131.1522	307.66	196.3373	230.008	114.5024	15.74911	244.6684	57.091	0.018991629	1.75449E-46	2.136797852
IMGA|Medtr3g104940.1	Mtr.27950.1.S1_at	27.13378	38.69873	51.85781	82.12218	63.49079	97.28258	39.23011	12.37058	80.96518	16.92558	0.026099852	5.11209E-09	2.063853168
IMGA|Medtr5g035610.1	Mtr.28125.1.S1_s_at	35.28819	62.86059	10.18506	29.32246	1214.922	62.32347	36.11128	26.34741	435.5226	675.1812	0.363789307	5.966E-152	12.06056866
IMGA|Medtr7g110580.1	Mtr.2814.1.S1_at	18.9422	15.26041	41.36515	96.01064	66.74866	80.98421	25.18925	14.12917	81.24784	14.63277	0.008817701	6.32883E-12	3.225496163
IMGA|AC233685_68.1	Mtr.28242.1.S1_at	5.799476	5.443935	6.516506	14.72005	14.17095	13.62055	5.919972	0.546344	14.17052	0.54975	5.09152E-05	0	2.393679824
	Mtr.28311.1.S1_s_at	80.21079	114.8032	114.7236	406.4682	280.5593	548.5185	103.2458	19.94898	411.8487	134.0606	0.016907206	3.7818E-158	3.989009759
	Mtr.2846.1.S1_at	710.5467	500.334	447.4935	1429.915	999.8743	930.5075	552.7914	139.1513	1120.099	270.5413	0.031981118	1.64809E-12	2.026260072
	Mtr.28491.1.S1_at	9.525346	13.15061	18.80234	59.19606	41.74922	60.65448	13.8261	4.675239	53.86659	10.51925	0.003824623	8.84346E-50	3.896007828
IMGA|AC233070_1030.1	Mtr.28786.1.S1_at	203.6667	215.6817	274.9307	442.5026	504.6157	605.0102	231.4264	38.15179	517.3762	82.00186	0.005412216	1.5515E-38	2.235597429
IMGA|Medtr2g027550.1	Mtr.29026.1.S1_at	18.12985	16.90922	26.12697	295.0916	379.2441	564.9921	20.38868	5.006839	413.1093	138.1003	0.007916587	0	20.26169636
IMGA|Medtr2g068760.1	Mtr.29059.1.S1_at	23.23766	23.9392	25.29934	58.99461	63.9856	61.42128	24.15873	1.048228	61.46716	2.49581	1.82629E-05	0	2.544304328
	Mtr.2927.1.S1_at	41.09755	48.25692	42.05794	85.54594	98.92869	83.93788	43.80414	3.886002	89.47083	8.230109	0.000965053	0	2.042520162
	Mtr.29298.1.S1_at	63.27649	50.67713	55.35996	124.0371	93.77761	122.7315	56.43786	6.368466	113.5154	17.10589	0.005631251	2.40424E-54	2.011334212
IMGA|Medtr3g086540.1	Mtr.29535.1.S1_at	60.29905	55.02296	122.1983	155.067	161.5375	184.865	79.17344	37.3539	167.1565	15.67353	0.019742124	0	2.111269818
IMGA|Medtr1g108330.1	Mtr.29620.1.S1_at	25.47109	39.89346	62.62193	250.3969	118.5869	251.6483	42.66216	18.72954	206.8774	76.46439	0.022497502	4.36999E-52	4.849200734
IMGA|Medtr2g009450.1	Mtr.29662.1.S1_at	111.2511	103.2637	146.3761	225.6544	444.1983	580.3819	120.297	22.93558	416.7449	178.9502	0.046582672	5.2484E-111	3.464300748
IMGA|Medtr3g031220.1	Mtr.29902.1.S1_at	78.12368	87.18082	98.88062	253.9369	218.475	266.3947	88.06171	10.40647	246.2689	24.8631	0.000527148	0	2.796548929
	Mtr.30156.1.S1_at	27.56446	28.63735	18.41301	41.93083	53.02588	68.76552	24.87161	5.618971	54.57408	13.48417	0.02441112	5.39594E-20	2.194232186
IMGA|Medtr8g018420.1	Mtr.30415.1.S1_at	12.35225	10.78895	11.89585	26.14947	19.11775	24.83444	11.67902	0.803894	23.36722	3.738429	0.006110932	6.3313E-140	2.000786453
	Mtr.30446.1.S1_at	40.49957	40.66082	20.66402	91.76661	75.30959	51.5723	33.94147	11.49889	72.88283	20.20675	0.044070415	4.47394E-09	2.147309068
	Mtr.30508.1.S1_at	260.5938	306.7833	406.8433	1305.354	951.9992	945.3541	324.7401	74.76006	1067.569	205.9544	0.004200749	2.23604E-66	3.28745641
IMGA|Medtr4g006710.1	Mtr.31163.1.S1_at	7.430685	7.823621	7.964915	15.73183	16.47532	18.71986	7.73974	0.276817	16.97567	1.555583	0.000535683	0	2.193312472
IMGA|Medtr5g055690.1	Mtr.31273.1.S1_at	6.536035	10.76555	7.805265	14.21162	25.79231	28.71901	8.368951	2.170371	22.90765	7.671833	0.034236307	4.00534E-31	2.73721832

IMGA|Medtr1g081550.1	Mtr.31385.1.S1_at	7.012977	6.712751	7.514167	12.88963	17.15538	18.79725	7.079965	0.404886	16.28075	3.049379	0.006603639	0	2.299552375
IMGA|Medtr5g029190.1	Mtr.31491.1.S1_s_at	75.3828	82.02181	75.05751	128.4454	181.4467	165.2948	77.48737	3.930303	158.3956	27.1658	0.006956236	1.9364E-278	2.044147727
	Mtr.31559.1.S1_s_at	76.78317	65.58529	64.17282	164.5994	139.5622	131.4753	68.84709	6.909035	145.2123	17.26978	0.0020665	1.08072E-81	2.10920021
	Mtr.31738.1.S1_at	144.7152	160.9258	190.3859	437.365	468.6839	469.865	165.3423	23.15345	458.6379	18.43239	6.75723E-05	0	2.773869281
	Mtr.31851.1.S1_at	9.914725	7.863057	9.206096	17.42981	14.74542	21.93778	8.994626	1.042053	18.03767	3.634504	0.014345901	4.63758E-51	2.005382807
	Mtr.31998.1.S1_at	200.2529	208.0679	214.8138	384.7244	440.2245	482.6087	207.7115	7.287014	435.8525	49.08841	0.001347735	0	2.098355002
IMGA|Medtr5g006670.1	Mtr.320.1.S1_at	10.19661	14.90479	13.40633	72.88808	31.91667	14.29285	12.83591	2.405364	39.6992	30.06287	0.197752542	2.30904E-83	3.092823717
IMGA|Medtr5g006670.1	Mtr.320.1.S1_x_at	14.62772	15.89785	13.81247	95.23319	45.86616	17.0261	14.77934	1.05093	52.70848	39.54997	0.172154555	0	3.5663614
	Mtr.3204.1.S1_at	11.87089	8.606409	8.563996	30.8582	55.94241	46.62411	9.68043	1.897108	44.47491	12.67946	0.009303564	1.855E-221	4.594311169
IMGA|Medtr1g083290.1	Mtr.32104.1.S1_s_at	11.76473	12.53335	11.1403	25.91819	20.8352	27.27049	11.81279	0.697769	24.67463	3.39309	0.003006774	1.2238E-223	2.088805414
IMGA|Medtr4g106590.1	Mtr.32159.1.S1_at	396.032	443.1323	569.8404	2072.429	1830.199	1463.45	469.6682	89.89133	1788.693	306.6035	0.002024058	1.7074E-142	3.808417632
	Mtr.32184.1.S1_at	103.7388	79.03165	70.53165	291.039	157.1369	335.0316	84.43402	17.25014	261.0692	92.65676	0.031491656	2.22815E-70	3.091990661
IMGA|Medtr7g088790.1	Mtr.32213.1.S1_at	12.95535	11.40101	7.975767	24.66698	52.86498	28.4892	10.77737	2.547694	35.34039	15.2966	0.051718615	1.33079E-62	3.279127992
IMGA|Medtr2g030400.1	Mtr.32248.1.S1_at	66.86498	69.77462	73.77586	146.9295	133.7647	157.5134	70.13849	3.469781	146.0692	11.89772	0.000446387	0	2.082582359
IMGA|Medtr3g008100.2	Mtr.32271.1.S1_at	7.431573	9.08278	9.126064	16.43267	18.0112	21.84452	8.546806	0.966062	18.7628	2.783112	0.003867643	6.21427E-75	2.195299554
IMGA|Medtr3g031220.1	Mtr.32279.1.S1_at	20.24903	23.77894	27.04141	58.85172	55.55597	67.03409	23.6898	3.397069	60.48059	5.909883	0.000729142	0	2.553022913
	Mtr.32284.1.S1_at	39.24566	35.60048	41.22603	168.0684	113.622	264.6829	38.69072	2.853538	182.1244	76.50505	0.031523282	0	4.70718622
	Mtr.32364.1.S1_at	25.99187	36.69929	49.4231	92.89878	56.22185	78.78622	37.37142	11.73006	75.96895	18.50006	0.037958021	1.20318E-08	2.032808581
IMGA|Medtr8g102150.2	Mtr.32738.1.S1_s_at	482.6432	551.6309	274.367	1147.753	1733.115	1118.901	436.2137	144.3454	1333.256	346.5878	0.014395744	5.09503E-27	3.056429258
IMGA|Medtr3g118390.1	Mtr.331.1.S1_at	41.58714	58.06626	32.69791	609.5084	740.8806	781.6542	44.1171	12.87202	710.6811	89.95865	0.0002211	0	16.10897006
IMGA|Medtr4g126920.2	Mtr.33150.1.S1_s_at	104.8679	73.95439	150.4552	328.544	199.865	509.3498	109.7592	38.48425	345.9196	155.4723	0.06304518	2.18884E-26	3.151623361
	Mtr.33212.1.S1_s_at	5.230026	6.100359	5.62493	11.28801	15.48555	31.17701	5.651772	0.435787	19.31686	10.48343	0.08708713	0	3.417840954
	Mtr.33499.1.S1_at	11.00271	9.079426	13.19267	14.80689	43.42721	54.89912	11.0916	2.05806	37.71107	20.6483	0.090418681	3.7116E-111	3.399966605
	Mtr.33808.1.S1_at	112.3284	136.6597	161.1528	380.6495	413.6985	416.3496	136.7136	24.41227	403.5659	19.89038	0.000125362	0	2.951906281
IMGA|Medtr3g117320.1	Mtr.33892.1.S1_at	27.72972	34.60832	31.3054	101.6295	100.4684	127.15	31.21448	3.440197	109.7493	15.08065	0.00092228	0	3.515973955
	Mtr.33931.1.S1_at	26.06397	21.05912	20.88985	131.6077	305.2525	165.1593	22.67098	2.939635	200.6732	92.10895	0.028688186	0	8.851543633
	Mtr.34114.1.S1_s_at	32.68721	24.5522	25.04068	763.4837	332.5567	379.1923	27.4267	4.562276	491.7442	236.4857	0.02727513	0	17.92939994
IMGA|AC233676_15.1	Mtr.34199.1.S1_at	13.99124	15.55987	22.03499	64.50831	39.19858	37.69896	17.19537	4.263991	47.13528	15.06416	0.029590579	4.97159E-34	2.741161762
	Mtr.3430.1.S1_at	38.31093	66.60216	62.11575	140.5326	154.0773	131.4544	55.67628	15.20522	142.0214	11.38469	0.001406675	0	2.550842426
IMGA|Medtr1g098080.1	Mtr.34598.1.S1_at	6.147004	5.131172	7.20351	14.79156	14.05178	21.08	6.160562	1.036236	16.64111	3.861942	0.01049823	1.05279E-68	2.701232723
	Mtr.34634.1.S1_at	21.62452	16.68172	29.2575	62.86542	52.81495	67.86406	22.52125	6.335662	61.18148	7.664575	0.0025341	0	2.716611495
IMGA|AC233676_31.1	Mtr.35009.1.S1_at	213.6663	180.5316	169.8883	395.9266	361.5316	472.4991	188.0288	22.83164	409.9858	56.80396	0.003283612	1.28354E-63	2.180441973
IMGA|Medtr4g100590.2	Mtr.35127.1.S1_s_at	34.94746	29.37937	34.15196	84.28809	86.22047	83.85865	32.82626	3.01148	84.78907	1.258085	1.02846E-05	0	2.582964498
	Mtr.35316.1.S1_at	32.57497	17.35974	21.72977	40.68389	62.21477	44.971	23.88816	7.833885	49.28989	11.39668	0.033490247	1.95146E-08	2.063360753
IMGA|Medtr1g110550.1	Mtr.35325.1.S1_at	42.98641	116.4415	69.16905	67.02926	233.6217	424.0084	76.199	37.22874	241.5531	178.6217	0.191577369	1.43694E-14	3.17002989
IMGA|Medtr7g011900.1	Mtr.35413.1.S1_at	10.31199	9.126064	8.464609	28.71414	19.52817	17.7371	9.300887	0.936016	21.99313	5.889051	0.02107988	5.7366E-122	2.364627576
	Mtr.35616.1.S1_at	7.37798	6.584245	5.765401	14.40577	13.07425	13.41844	6.575875	0.806322	13.63282	0.691165	0.000325394	0	2.073156598
	Mtr.35660.1.S1_at	116.865	91.91522	97.16023	296.1661	276.7182	257.1632	101.9801	13.1547	276.6825	19.50146	0.000210581	0	2.713101842
IMGA|Medtr3g087900.1	Mtr.35677.1.S1_at	7.606138	6.047388	9.577951	33.61266	22.91058	26.08709	7.743826	1.769304	27.53677	5.496349	0.004034891	1.23046E-83	3.555965124
	Mtr.35741.1.S1_at	54.17107	41.03182	52.11819	253.4144	191.365	345.395	49.10702	7.068262	263.3915	77.49817	0.008844166	0	5.363621087
	Mtr.35967.1.S1_s_at	70.39837	76.14996	53.63062	129.365	135.784	210.5316	66.72631	11.70014	158.5602	45.12287	0.02697225	4.29957E-42	2.376277224
	Mtr.35992.1.S1_at	4.089379	5.778014	5.992404	9.679979	15.20662	12.93323	5.286599	1.04235	12.60661	2.77776	0.012915472	4.88401E-34	2.384634873
IMGA|Medtr8g036660.1	Mtr.3624.1.S1_at	7.16813	5.686447	8.570647	15.82867	11.77923	60.51959	7.141741	1.442281	29.37583	27.04717	0.228141494	4.6147E-157	4.113258559
	Mtr.36965.1.S1_at	35.85737	33.11511	35.8072	121.4906	85.70135	258.0586	34.92656	1.568962	155.0835	90.95668	0.084064347	0	4.4402745
	Mtr.37204.1.S1_at	6.217299	9.270986	9.516503	25.38871	32.23745	25.92814	8.334929	1.838026	27.85143	3.807966	0.00132736	0	3.34153167
	Mtr.37222.1.S1_at	411.1834	424.7119	408.7944	814.6743	989.3727	901.0677	414.8966	8.583867	901.7049	87.35091	0.000656378	0	2.173324625
IMGA|Medtr4g108150.1	Mtr.37275.1.S1_at	1038.515	745.6983	1303.497	3359.599	3404.58	4321.127	1029.237	279.0151	3695.102	542.6198	0.001634449	0	3.590137829
	Mtr.37276.1.S1_at	6.135126	8.930318	5.977789	10.29493	16.92189	69.99632	7.014411	1.661088	32.40438	32.72376	0.250664858	1.9147E-154	4.619686291
IMGA|Medtr3g083130.1	Mtr.37316.1.S1_at	789.3712	690.5723	1159.585	4712.925	3905.481	4371.193	879.843	247.2493	4329.866	405.3052	0.000229344	0	4.921180848
	Mtr.37376.1.S1_s_at	12.25979	13.74831	23.12368	50.73094	29.72748	46.97581	16.37726	5.889783	42.47807	11.20083	0.023326683	1.64585E-14	2.593723202

IMGA|Medtr7g093870.1	Mtr.37393.1.S1_at	18.48222	14.8738	17.82385	116.3723	156.3207	157.0633	17.05995	1.921669	143.2521	23.28156	0.0007267	0	8.396980172
IMGA|Medtr2g045280.1	Mtr.37395.1.S1_at	33.78652	12.94468	10.95492	113.9982	119.703	362.2439	19.22871	12.64663	198.6484	141.7066	0.094287341	2.4646E-133	10.33082426
	Mtr.37425.1.S1_at	348.9067	442.8645	476.1337	880.1339	773.1509	1102.865	422.6349	65.98183	918.7166	168.2091	0.008935421	9.13135E-39	2.173782902
IMGA|Medtr3g089970.1	Mtr.37441.1.S1_s_at	24.76271	23.18786	33.1299	57.01841	76.80465	46.67791	27.02682	5.343751	60.16699	15.30818	0.024009882	6.49161E-27	2.226195304
	Mtr.37560.1.S1_at	16.16777	12.06213	27.22738	64.7597	67.79556	51.6428	18.48576	7.843856	61.39935	8.58469	0.003075325	0	3.321440995
IMGA|Medtr4g093580.2	Mtr.37568.1.S1_at	237.7355	285.9898	366.0045	880.4673	483.1001	724.7727	296.5766	64.78652	696.1134	200.2279	0.030263674	1.24232E-26	2.347162228
	Mtr.37585.1.S1_a_at	7.515745	9.126064	6.683858	19.62933	24.29226	22.80882	7.775222	1.241607	22.24347	2.382317	0.000735191	0	2.860814227
IMGA|Medtr7g088820.1	Mtr.37657.1.S1_at	48.87242	41.4318	82.20149	871.3345	1108.398	1020.422	57.5019	21.71158	1000.051	119.8372	0.00017913	0	17.39162268
IMGA|Medtr6g086170.1	Mtr.37708.1.S1_at	32.58118	56.78096	32.43024	188.9419	202.7799	260.2416	40.59746	14.01553	217.3211	37.80874	0.001615442	9.7483E-106	5.353071576
	Mtr.37718.1.S1_at	154.9756	113.5506	229.161	304.2524	332.6175	527.1449	165.8957	58.57373	388.0049	121.3305	0.04614385	5.10412E-11	2.338848423
	Mtr.37724.1.S1_at	16.29376	10.46297	10.33094	71.82149	114.3833	101.7961	12.36256	3.40516	96.00031	21.86483	0.002814435	0	7.765410015
	Mtr.37724.1.S1_s_at	4.116225	6.224902	5.811345	84.25431	139.4902	111.0316	5.384157	1.117361	111.5921	27.62222	0.002648642	0	20.72600194
IMGA|Medtr8g022810.1	Mtr.37773.1.S1_at	84.82202	90.3486	147.4147	232.2093	307.2449	357.3125	107.5284	34.65286	298.9223	62.96549	0.009937179	1.10672E-21	2.779936875
	Mtr.37779.1.S1_at	440.5316	490.865	494.7937	889.5516	1299.365	1534.168	475.3968	30.25793	1241.028	326.2439	0.015508717	0	2.610510594
IMGA|Medtr3g083760.1	Mtr.37781.1.S1_at	77.6739	63.1611	57.4594	96.23267	175.8407	140.7385	66.09814	10.42239	137.604	39.89649	0.039803139	1.4465E-32	2.081813408
	Mtr.37869.1.S1_at	276.2466	328.8009	314.4967	1738.188	535.5316	745.358	306.5147	27.17119	1006.359	642.407	0.132478671	0	3.283232751
IMGA|Medtr7g035060.1	Mtr.37906.1.S1_at	117.0873	159.3216	170.7497	321.7286	280.865	315.7436	149.0529	28.26655	306.1124	22.06873	0.001619723	0	2.053716467
IMGA|Medtr7g093950.1	Mtr.37969.1.S1_at	61.70518	58.28514	37.11618	121.5954	106.8852	120.3207	52.36884	13.31941	116.2671	8.149934	0.002092079	0	2.220157497
	Mtr.37972.1.S1_at	47.26001	48.69832	48.86499	180.449	242.4432	195.6578	48.27444	0.882464	206.1833	32.30959	0.001068726	0	4.271066028
	Mtr.38079.1.S1_at	15.30218	15.64152	22.05979	38.91384	36.69393	41.57204	17.66783	3.807331	39.05993	2.442338	0.001209971	0	2.210794203
IMGA|Medtr5g035610.1	Mtr.38093.1.S1_at	10.17269	9.318778	6.858476	10.41682	190.365	11.75835	8.783315	1.720769	70.84671	103.508	0.35773223	0	8.066056725
IMGA|Medtr8g045570.1	Mtr.38110.1.S1_at	12.30727	14.10344	14.5869	260.7642	107.3595	193.6313	13.66587	1.201156	187.2517	76.90108	0.017405386	0	13.70214192
IMGA|Medtr2g008160.3	Mtr.38116.1.S1_at	4.154127	6.035374	5.545236	13.34335	21.31176	42.61229	5.244912	0.975919	25.7558	15.13208	0.079131008	4.0648E-290	4.910625707
IMGA|Medtr2g008160.2	Mtr.38116.1.S1_s_at	12.54868	13.8545	11.21766	105.7102	142.2187	191.4635	12.54028	1.318439	146.4641	43.03399	0.005739076	0	11.67949375
IMGA|Medtr3g086830.1	Mtr.38121.1.S1_at	94.86102	101.0204	80.83397	181.3083	175.5535	203.9228	92.23847	10.34561	186.9282	14.99642	0.000843075	0	2.026575179
	Mtr.38171.1.S1_at	13.04095	14.07427	18.21923	29.32158	81.30389	66.24714	15.11148	2.740533	58.95753	26.74685	0.047607164	5.1271E-169	3.901505456
	Mtr.38202.1.S1_at	13.22113	13.18562	19.1999	35.45164	35.49687	47.73979	15.20222	3.462143	39.56277	7.081543	0.005874617	3.64012E-34	2.602434158
	Mtr.38252.1.S1_at	16.95988	10.6698	11.15752	46.54885	114.6396	122.3827	12.92907	3.499295	94.52372	41.72745	0.02791325	0	7.310945093
IMGA|Medtr3g069190.1	Mtr.38272.1.S1_at	103.3999	107.365	94.80107	220.4657	295.78	294.2128	101.8553	6.422791	270.1529	43.03746	0.002583477	0	2.652319902
IMGA|Medtr5g020020.1	Mtr.38274.1.S1_at	32.13217	28.908	32.4198	199.4459	168.859	159.995	31.15332	1.949822	176.1	20.69828	0.000269705	0	5.652685794
IMGA|Medtr1g088660.1	Mtr.38347.1.S1_at	46.25345	37.40681	44.74709	86.75794	74.53844	127.78	42.80245	4.73307	96.35878	27.88902	0.030522733	1.58542E-85	2.25124443
IMGA|Medtr4g098450.1	Mtr.38359.1.S1_at	13.03055	14.51741	18.41612	45.28275	38.36645	36.90664	15.32136	2.781336	40.18528	4.474472	0.001219641	0	2.622827599
	Mtr.38500.1.S1_at	9.648972	7.752487	9.709732	29.84355	19.56162	47.57783	9.037064	1.112891	32.32767	14.17234	0.046977401	1.0899E-287	3.577231262
IMGA|Medtr3g114750.1	Mtr.38513.1.S1_at	15.54276	16.89893	12.01859	33.23098	33.82431	37.46572	14.8201	2.519149	34.84033	2.29292	0.000524543	0	2.35088463
IMGA|Medtr3g101290.1	Mtr.38547.1.S1_at	22.2115	57.79518	30.68241	96.65501	91.7716	78.19831	36.89636	18.58789	88.87497	9.563224	0.012577076	0	2.408773333
IMGA|Medtr5g017850.1	Mtr.38635.1.S1_at	21.39663	24.36451	19.12032	80.66583	62.16779	106.5124	21.62715	2.629685	83.11534	22.27355	0.008980775	0	3.843100982
	Mtr.38695.1.S1_at	9.98712	9.6764	10.05032	20.30723	24.94818	37.49419	9.904613	0.200148	27.5832	8.89131	0.02622257	0	2.784884074
IMGA|Medtr5g012000.1	Mtr.38812.1.S1_at	190.9075	242.9547	179.5423	511.865	581.0316	800.0499	204.4682	33.81128	630.9822	150.4458	0.008706034	7.9388E-106	3.08596771
	Mtr.38883.1.S1_at	44.36502	63.99436	75.90876	150.8617	235.2527	315.3163	61.42271	15.92834	233.8103	82.23679	0.023490348	2.10848E-78	3.806576493
IMGA|AC233577_31.1	Mtr.38932.1.S1_at	2397.093	2288.986	2300.654	5049.615	4161.697	5459.379	2328.911	59.33489	4890.23	663.3608	0.002638662	0	2.099792952
IMGA|Medtr1g042820.1	Mtr.39059.1.S1_at	60.84784	90.77781	92.62179	152.3481	160.4188	204.3932	81.41581	17.83623	172.3867	28.01066	0.009004519	1.00953E-18	2.117361365
IMGA|AC233577_31.1	Mtr.39132.1.S1_at	433.5328	413.8149	442.8309	1081.032	801.6844	935.9447	430.0595	14.81655	939.5536	139.7086	0.00328036	0	2.184705752
	Mtr.39165.1.S1_at	85.65669	54.62668	140.3524	381.9129	162.173	351.0158	93.54525	43.40387	298.3672	118.955	0.048729636	2.9954E-16	3.189549783
	Mtr.39214.1.S1_at	12.4982	21.67785	12.15248	67.77471	80.03165	138.6291	15.44284	5.402438	95.47847	37.86869	0.02227797	3.2849E-145	6.182700366
IMGA|Medtr5g064240.1	Mtr.39322.1.S1_at	1137.935	1159.418	1491.287	2501.351	2303.198	2773.384	1262.88	198.0974	2525.978	236.0582	0.002079352	0	2.000172687
	Mtr.39456.1.S1_at	10.31827	10.19191	10.32432	27.52149	19.16468	22.62092	10.27817	0.074761	23.10236	4.199159	0.006133344	0	2.247711639
IMGA|Medtr3g096410.1	Mtr.39643.1.S1_at	22.52083	19.14705	15.00937	70.25524	54.39954	83.1161	18.89242	3.762194	69.25696	14.38428	0.004213894	6.1679E-119	3.665860476
IMGA|Medtr5g063200.1	Mtr.39659.1.S1_at	184.6233	202.3595	206.5214	470.4776	431.9841	463.383	197.8347	11.62914	455.2815	20.48565	4.58721E-05	0	2.301322518
IMGA|Medtr2g086880.1	Mtr.39754.1.S1_at	21.52018	25.19041	21.71356	57.69465	41.18799	55.5952	22.80805	2.065451	51.49261	8.985592	0.005735346	7.547E-128	2.257650943
IMGA|Medtr1g083290.1	Mtr.39812.1.S1_s_at	47.98632	56.41616	68.35637	236.8147	198.644	281.7559	57.58628	10.23531	239.0716	41.60187	0.0018369	3.9985E-207	4.15153655

	Mtr.39820.1.S1_at	18.56997	16.61219	22.40687	44.50604	74.56945	98.73294	19.19634	2.947683	72.60281	27.16689	0.027654523	3.6327E-216	3.782116279
	Mtr.39861.1.S1_at	29.53538	26.26975	37.36008	89.81152	64.36498	122.5047	31.05507	5.699206	92.22706	29.14502	0.023421836	3.81324E-77	2.969790921
	Mtr.39929.1.S1_at	46.91311	58.25038	13.60913	142.9088	184.1734	72.43063	39.59088	23.20391	133.171	56.50427	0.056772761	2.84322E-12	3.363678136
IMGA|Medtr8g020630.1	Mtr.40021.1.S1_at	48.1539	74.22485	66.72545	243.3933	423.6252	455.9668	63.03473	13.42161	374.3284	114.5404	0.009480709	0	5.938447184
	Mtr.40041.1.S1_at	7.665786	5.612967	9.526719	27.85491	24.58882	31.56909	7.601824	1.95766	28.00428	3.492532	0.000909445	0	3.683889112
IMGA|Medtr7g072420.1	Mtr.40048.1.S1_at	203.3997	204.9721	380.7369	689.8037	649.7324	1104.875	263.0362	101.9348	814.8039	252.0071	0.024547319	6.88483E-21	3.097687076
	Mtr.40058.1.S1_x_at	189.6822	160.3842	145.125	1047.481	2312.485	1170.014	165.0638	22.64419	1509.993	697.6735	0.028911645	0	9.147935951
	Mtr.40066.1.S1_at	14.5597	10.35004	10.04051	217.6691	534.2694	603.9153	11.65008	2.52455	451.9513	205.861	0.020757571	0	38.79383087
	Mtr.40069.1.S1_at	41.07693	40.77352	40.61099	82.2468	88.33778	96.16467	40.82048	0.236492	88.91642	6.976953	0.000282535	0	2.17823048
IMGA|Medtr8g045520.1	Mtr.40106.1.S1_s_at	88.36498	111.1638	102.3175	193.2912	338.789	193.9208	100.6154	11.49433	242.0003	83.82205	0.044366029	1.0218E-100	2.405200962
IMGA|Medtr7g072480.1	Mtr.40120.1.S1_at	5.158958	5.491681	4.732966	10.71875	70.16478	53.34328	5.127868	0.380312	44.74227	30.64214	0.088716655	0	8.725315945
IMGA|Medtr2g035100.1	Mtr.40147.1.S1_s_at	77.28804	29.54136	65.1869	608.1487	569.2039	811.3353	57.33877	24.82199	662.896	130.0187	0.001372916	0	11.56104319
IMGA|Medtr5g030620.1	Mtr.40239.1.S1_at	780.9997	829.2471	901.8815	3483.102	3984.501	5564.666	837.3761	60.8495	4344.09	1086.372	0.005050149	0	5.187740427
IMGA|Medtr7g065590.1	Mtr.40278.1.S1_at	37.17339	27.79895	29.69033	120.5548	117.0167	123.0434	31.55422	4.957376	120.2049	3.028544	1.21768E-05	0	3.809472308
IMGA|Medtr4g057450.1	Mtr.40322.1.S1_at	118.5634	161.9538	121.2943	791.6703	720.2998	675.3798	133.9372	24.30148	729.1167	58.64444	8.41336E-05	0	5.443721669
IMGA|Medtr1g093120.1	Mtr.40329.1.S1_at	12.48837	17.4637	10.5481	36.69929	47.69832	48.39362	13.50006	3.567071	44.26375	6.560227	0.002039782	1.87086E-50	3.278782642
	Mtr.40333.1.S1_x_at	76.89815	87.10835	111.8262	251.9561	175.0722	248.3994	91.94424	17.95917	225.1426	43.39864	0.007974933	9.03905E-38	2.448686095
	Mtr.4034.1.S1_at	35.43508	62.07787	30.54387	83.75807	106.8939	96.53514	42.68561	16.97133	95.72904	11.58896	0.011069524	2.22045E-15	2.242653693
	Mtr.40344.1.S1_at	31.09213	78.34506	116.2929	103.3184	426.5218	257.5926	75.24338	42.68501	262.4776	161.657	0.124432065	3.01914E-14	3.488381538
IMGA|Medtr1g088640.1	Mtr.40363.1.S1_at	164.6316	132.9231	152.0395	469.5334	438.9035	672.7684	149.8647	15.9657	527.0684	127.1059	0.006982529	0	3.516960882
	Mtr.40399.1.S1_at	8.639519	5.701231	15.62	154.1298	260.8396	240.4117	9.986915	5.094809	218.4604	56.6405	0.003152134	0	21.87465946
IMGA|AC146550_1005.1	Mtr.40419.1.S1_at	119.7264	117.0316	93.14477	227.0316	193.5039	290.365	109.9676	14.63117	236.9668	49.1889	0.012783329	4.37817E-51	2.154878602
	Mtr.40420.1.S1_at	341.4676	306.8392	212.7181	594.2312	877.1496	619.343	287.0083	66.62625	696.9079	156.598	0.014010748	1.63539E-26	2.428180451
	Mtr.40475.1.S1_at	32.70806	64.09136	37.95029	229.1899	139.2032	102.4042	44.91657	16.81144	156.9324	65.22575	0.044994448	8.21792E-31	3.493864903
IMGA|Medtr7g077340.1	Mtr.40476.1.S1_at	30.56049	31.12755	40.39833	59.15743	78.89479	112.1794	34.02879	5.523462	83.41056	26.7979	0.035320641	4.37037E-54	2.451176179
IMGA|Medtr6g091680.1	Mtr.40504.1.S1_at	79.55843	69.36498	132.7084	962.6712	650.4243	569.4473	93.87727	34.01277	727.5143	207.6377	0.006444692	2.0295E-228	7.749631765
	Mtr.40506.1.S1_at	109.2814	120.5842	132.116	341.5678	253.4762	348.4003	120.6606	11.41751	314.4814	52.94243	0.003444868	5.0486E-190	2.606331946
	Mtr.40509.1.S1_at	8.389205	6.327177	17.57027	31.097	20.23174	106.5296	10.76222	5.985415	52.61945	47.00258	0.20073771	9.06039E-34	4.889275863
	Mtr.40517.1.S1_s_at	74.83664	49.15777	57.33784	141.9402	210.5457	152.6038	60.44408	13.11822	168.3633	36.91811	0.008834251	4.54772E-46	2.785438238
	Mtr.40518.1.S1_at	14.36453	11.47832	10.96993	41.71877	99.96164	47.54304	12.27093	1.830846	63.07448	32.07767	0.051974634	0	5.140155764
IMGA|Medtr4g013930.1	Mtr.40553.1.S1_at	24.53559	8.684243	16.23012	167.5316	301.2461	173.4538	16.48332	7.928704	214.0772	75.54854	0.010778247	0	12.98750636
	Mtr.40588.1.S1_at	134.561	170.6796	213.506	491.1134	400.3532	469.1668	172.9155	39.51995	453.5445	47.35393	0.001401753	0	2.622924932
IMGA|Medtr1g093100.1	Mtr.40605.1.S1_at	124.7744	111.1664	125.6055	488.5575	665.4763	437.1023	120.5155	8.10714	530.3787	119.7933	0.004096922	0	4.400918495
	Mtr.40613.1.S1_at	146.2729	164.9437	172.5546	586.8967	473.2227	747.7202	161.2571	13.52315	602.6132	137.922	0.005271917	0	3.736972518
	Mtr.40631.1.S1_at	45.365	38.15929	43.9468	92.75501	60.16822	118.1743	42.49036	3.817263	90.36584	29.07675	0.0474607	1.2426E-104	2.126737326
IMGA|Medtr3g078640.1	Mtr.40678.1.S1_at	268.5188	304.5316	306.5264	563.6912	714.8313	731.7072	293.1923	21.39111	670.0766	92.51802	0.002345877	1.5725E-204	2.285450765
IMGA|Medtr3g101320.1	Mtr.40696.1.S1_at	142.5316	135.1027	137.8967	336.2713	326.1722	322.6225	138.5104	3.752298	328.3553	7.081455	2.10876E-06	0	2.370619565
IMGA|Medtr1g081660.1	Mtr.40711.1.S1_at	25.25278	29.13526	26.6042	127.2201	230.8434	214.11	26.99741	1.970882	190.7245	55.62922	0.007009176	0	7.064547665
	Mtr.40765.1.S1_at	8.823809	9.126064	9.140121	57.10416	10.77582	32.36467	9.029998	0.178703	33.41488	23.18202	0.142567264	0	3.700430388
IMGA|Medtr5g006950.1	Mtr.40769.1.S1_at	16.11017	18.82003	18.28251	71.22379	85.36498	84.05159	17.73757	1.434768	80.21345	7.812924	0.000168143	0	4.52223533
IMGA|Medtr7g092540.1	Mtr.4083.1.S1_at	10.44006	13.84334	20.03676	28.21736	28.8988	35.0203	14.77338	4.86548	30.71216	3.746489	0.010858673	1.72307E-13	2.078884322
IMGA|Medtr7g092540.1	Mtr.4083.1.S1_s_at	24.53199	16.90922	47.7414	66.52445	82.58281	80.67086	29.72754	16.0593	76.59271	8.771614	0.011369155	0	2.576490217
IMGA|Medtr3g088150.1	Mtr.40882.1.S1_at	318.865	292.2134	255.0119	565.153	484.7809	747.365	288.6967	32.07148	599.0996	134.5432	0.017734423	4.50497E-63	2.075186668
IMGA|Medtr2g008710.2	Mtr.40911.1.S1_s_at	387.2835	344.2252	415.6736	920.6983	1063.651	913.8129	382.3941	35.97428	966.054	84.59134	0.000388507	0	2.526330935
	Mtr.40959.1.S1_at	47.73323	41.90129	56.82374	315.5004	113.375	415.8479	48.81942	7.520291	281.5744	154.0639	0.059194683	0	5.767672021
IMGA|Medtr5g020800.1	Mtr.410.1.S1_s_at	126.8057	145.4174	226.7699	838.7786	556.7987	599.3938	166.331	53.16246	664.9904	152.0044	0.005832552	2.36525E-59	3.99799398
IMGA|Medtr4g069810.1	Mtr.41019.1.S1_at	6.310247	15.34715	12.53493	24.78753	42.06185	42.45537	11.39744	4.624589	36.43492	10.08885	0.017431043	6.76894E-21	3.196762689
IMGA|Medtr3g100470.1	Mtr.41053.1.S1_at	831.9686	885.6983	857.3988	2222.365	2318.09	2769.033	858.3553	26.87761	2436.496	291.936	0.000736571	0	2.838563861
	Mtr.41059.1.S1_at	37.32047	58.84385	67.27872	164.8299	126.9301	177.1867	54.48101	15.4483	156.3155	26.18781	0.004391505	3.41527E-30	2.869174338
IMGA|Medtr7g065260.1	Mtr.41149.1.S1_at	16.39238	13.11352	17.40358	122.9875	119.6293	122.628	15.63649	2.242691	121.7483	1.843899	3.73034E-07	0	7.786161915

IMGA|Medtr4g127930.1	Mtr.41159.1.S1_at	78.15696	157.9843	135.9724	353.243	379.187	397.806	124.0379	41.23016	376.7453	22.38159	0.000734637	0	3.037340626
	Mtr.41238.1.S1_at	12.57002	9.026404	14.07079	33.86814	49.0697	39.05176	11.88907	2.590216	40.6632	7.727829	0.003621505	1.68353E-82	3.420217152
IMGA|Medtr4g127530.1	Mtr.41282.1.S1_s_at	53.0335	56.8737	50.84726	99.9858	130.365	115.3046	53.58482	3.050812	115.2185	15.18977	0.00232564	3.0378E-268	2.150207432
	Mtr.41355.1.S1_at	20.60956	21.67906	19.02923	63.4484	50.36681	71.71145	20.43928	1.333096	61.84222	10.76259	0.002711448	0	3.025654828
IMGA|Medtr2g035170.1	Mtr.41478.1.S1_at	162.8911	130.2459	225.3548	526.0323	405.0292	482.4438	172.8306	48.32722	471.1685	61.2845	0.002698821	0	2.726186487
IMGA|AC233685_102.1	Mtr.41727.1.S1_at	27.14518	44.41395	29.03905	55.2536	75.22553	74.03656	33.53273	9.470875	68.1719	11.20336	0.014978492	2.37539E-10	2.032995905
	Mtr.41872.1.S1_at	21.71056	24.85713	18.72783	79.65895	59.29739	59.56862	21.76517	3.065014	66.17499	11.67824	0.003113112	5.4907E-139	3.040406874
	Mtr.41930.1.S1_at	23.0593	20.34346	24.7807	89.69831	43.49165	123.1983	22.72782	2.237114	85.46276	40.02178	0.053493442	0	3.760270761
IMGA|Medtr3g094630.1	Mtr.42141.1.S1_s_at	20.34135	16.6791	12.88861	39.30075	48.79125	57.3278	16.63635	3.726555	48.47327	9.017728	0.004829471	1.52443E-49	2.913695504
	Mtr.42258.1.S1_at	97.76064	127.0548	95.53165	187.6064	204.2054	291.5316	106.7824	17.59179	227.7812	55.82987	0.023161413	1.00917E-32	2.133134716
	Mtr.42268.1.S1_at	123.0703	97.32044	76.44371	401.3924	311.9861	624.8338	98.94481	23.35569	446.0708	161.1383	0.020970555	3.8861E-146	4.508278384
	Mtr.42416.1.S1_at	11.27856	5.881525	11.2678	29.69818	22.22573	40.41916	9.475962	3.112878	30.78102	9.144921	0.018779193	2.04163E-32	3.248327127
	Mtr.42847.1.S1_at	1133.115	1877.513	1506.774	3245.793	3506.969	3624.025	1505.801	372.1999	3458.929	193.6382	0.001284932	0	2.2970694
IMGA|Medtr3g072980.1	Mtr.42916.1.S1_at	20.14282	7.147873	9.256912	170.5087	110.0421	144.8186	12.18253	6.973995	141.7898	30.34691	0.001962469	2.5126E-227	11.63877558
IMGA|Medtr2g035150.1	Mtr.42966.1.S1_at	14.42694	17.80529	11.16009	116.0592	85.56017	92.41984	14.46411	3.322754	98.01306	16.00032	0.000898018	0	6.77629516
IMGA|Medtr1g079490.1	Mtr.42985.1.S1_at	18.04374	16.25536	10.25774	46.85019	168.6647	287.5466	14.85228	4.078225	167.6872	120.3512	0.092830271	0	11.29033169
IMGA|Medtr5g010640.1	Mtr.42989.1.S1_at	702.17	754.2489	1156.508	5608.18	8824.961	9343.556	870.9756	248.6454	7925.566	2023.597	0.003898847	0	9.09964185
	Mtr.43009.1.S1_s_at	13.32789	12.04529	60.36067	186.2972	135.7586	314.3258	28.57795	27.53211	212.1272	92.04323	0.029678746	7.63344E-31	7.422759241
IMGA|Medtr7g021850.1	Mtr.43023.1.S1_at	27.22738	28.52561	39.79098	153.1132	91.86498	69.86498	31.84799	6.909391	104.9477	43.13858	0.044203001	5.25895E-75	3.295269857
	Mtr.43048.1.S1_at	11.39794	13.36931	17.72976	31.6169	175.8574	62.57476	14.16567	3.24016	90.01636	75.93492	0.158950701	0	6.354542621
	Mtr.4306.1.S1_s_at	20.918	33.1693	54.65712	51.69831	188.3541	160.5316	36.24814	17.07898	133.528	72.2191	0.085683678	5.87058E-23	3.683720553
IMGA|Medtr3g055120.1	Mtr.43078.1.S1_at	18.86062	37.69896	38.32617	60.29198	73.37582	59.53165	31.62858	11.06182	64.39982	7.78274	0.013732531	3.02647E-13	2.03612726
	Mtr.43146.1.S1_at	171.7751	233.973	435.4639	1104.615	792.5654	1782.698	280.404	137.8399	1226.626	506.2172	0.03539585	1.33534E-32	4.374496434
IMGA|Medtr2g084000.1	Mtr.43159.1.S1_at	59.00946	100.4649	70.15981	615.0442	922.5762	1116.033	76.54472	21.45259	884.5512	252.6499	0.005260634	0	11.55600606
IMGA|Medtr5g016450.2	Mtr.43175.1.S1_s_at	187.356	175.0292	176.8934	362.8672	509.1983	438.1843	179.7595	6.644443	436.75	73.17608	0.003748043	0	2.429634256
IMGA|Medtr7g065150.1	Mtr.43230.1.S1_at	713.2393	568.8513	465.3384	1147.865	1199.038	1507.695	582.4763	124.5108	1284.866	194.6646	0.006234248	1.50206E-22	2.20586851
IMGA|Medtr7g065150.1	Mtr.43231.1.S1_s_at	388.865	299.5367	233.1071	695.2753	641.8011	848.4524	307.1696	78.15896	728.5096	107.2594	0.005332155	9.89575E-21	2.371685199
	Mtr.43254.1.S1_at	35.67221	37.69896	63.19831	115.134	74.34408	120.5269	45.52316	15.34064	103.335	25.25126	0.027553763	6.69631E-11	2.269943487
	Mtr.43259.1.S1_at	20.79776	23.34974	26.2624	131.9594	104.5798	162.7488	23.46997	2.7343	133.096	29.10112	0.00289637	0	5.670906994
	Mtr.43278.1.S1_at	140.5169	149.9325	137.0873	291.6531	285.9719	285.1019	142.5122	6.651033	287.5756	3.557909	4.84418E-06	0	2.01790114
	Mtr.43284.1.S1_at	16.05452	15.4479	27.02278	91.78306	64.58843	78.06458	19.5084	6.514711	78.14536	13.59749	0.002530977	8.55926E-55	4.005728438
	Mtr.43294.1.S1_at	1978.691	1016.71	1402.034	2606.111	4706.006	3311.824	1465.812	484.1511	3541.314	1068.592	0.037501464	1.12686E-13	2.415940762
IMGA|Medtr1g087920.1	Mtr.43313.1.S1_at	202.2595	184.865	228.7882	522.835	1089.714	565.865	205.3042	22.11934	726.1379	315.6001	0.046330058	0	3.536887436
IMGA|Medtr7g038050.1	Mtr.43323.1.S1_at	23.29658	21.61082	24.42585	56.69831	33.59702	68.58382	23.11108	1.41665	52.95972	17.7905	0.044258046	1.4066E-291	2.29152896
IMGA|Medtr5g077510.1	Mtr.43363.1.S1_at	26.2383	33.02616	36.62856	94.52274	96.56862	98.36498	31.96434	5.275887	96.48545	1.92247	3.76096E-05	0	3.018534271
IMGA|Medtr3g092430.1	Mtr.43392.1.S1_s_at	22.5981	25.64592	33.52523	54.78024	64.8287	83.36498	27.25642	5.638781	67.65797	14.50088	0.010841924	2.3055E-35	2.482276852
IMGA|Medtr7g023690.1	Mtr.43467.1.S1_at	145.148	79.35044	160.0485	905.5506	1793.554	1597.753	128.1823	42.94089	1432.286	466.5532	0.008516642	0	11.1738179
IMGA|Medtr3g117420.1	Mtr.43497.1.S1_at	112.1983	115.399	175.0316	282.355	203.9552	324.4914	134.2097	35.38908	270.2672	61.17052	0.028979957	2.75554E-11	2.013768711
IMGA|Medtr7g072760.1	Mtr.43563.1.S1_at	98.27652	70.53165	99.56104	193.0156	160.3362	240.1613	89.4564	16.4019	197.8377	40.1304	0.012348821	2.48727E-30	2.211554396
	Mtr.43594.1.S1_at	7.502404	7.302674	7.989109	20.82979	13.27337	21.34945	7.598062	0.353074	18.4842	4.520192	0.014160155	0	2.432752307
	Mtr.43656.1.S1_at	151.5511	182.7906	96.9585	446.8651	764.828	680.788	143.7667	43.44233	630.827	164.764	0.007756055	5.3161E-84	4.38785076
IMGA|Medtr3g109490.1	Mtr.43680.1.S1_at	35.79086	23.2357	28.32435	50.61004	133.1939	172.7589	29.11697	6.314999	118.8543	62.3242	0.068124641	9.2275E-134	4.081959141
	Mtr.43715.1.S1_at	26.76612	55.25121	36.94422	87.02371	61.19831	100.0796	39.65385	14.43456	82.7672	19.78703	0.038070257	2.29981E-07	2.087242525
	Mtr.43895.1.S1_at	13.00287	13.56633	12.60635	44.34725	57.90361	63.81126	13.05852	0.482403	55.35404	9.979333	0.001841382	0	4.238922243
IMGA|Medtr4g069770.1	Mtr.43908.1.S1_at	69.59262	90.44767	59.90426	137.7143	197.8986	193.0904	73.31485	15.60821	176.2344	33.44592	0.008462396	3.28301E-30	2.403802492
IMGA|Medtr5g020800.1	Mtr.43916.1.S1_at	14.95275	19.50948	21.74522	51.63354	104.2787	75.3491	18.73582	3.461692	77.08712	26.36559	0.01909235	2.1874E-187	4.114425164
IMGA|Medtr5g013030.1	Mtr.43917.1.S1_at	26.41435	29.68309	35.07868	178.4265	64.12355	177.6753	30.39204	4.375456	140.0751	65.77705	0.044930706	0	4.608940919
IMGA|Medtr5g019120.3	Mtr.43942.1.S1_s_at	280.9991	244.9578	274.9222	1181.341	969.6592	1704.651	266.9597	19.29493	1285.217	378.346	0.009621936	0	4.814273844
IMGA|Medtr5g062190.1	Mtr.43944.1.S1_at	244.0288	395.5417	320.6363	803.782	851.9199	970.3288	320.0689	75.75805	875.3435	85.70856	0.001095353	0	2.734859405
IMGA|Medtr3g113710.2	Mtr.43953.1.S1_at	154.365	116.2268	146.6933	334.365	205.4763	298.882	139.095	20.17253	279.5744	66.57822	0.024950547	1.68022E-33	2.009952945

IMGA|Medtr3g113710.2	Mtr.43953.1.S1_s_at	194.5316	179.2182	184.5898	466.3964	261.7357	408.4942	186.1132	7.769552	378.8754	105.4963	0.034307494	0	2.035725486
IMGA|Medtr3g113710.2	Mtr.43954.1.S1_at	249.5418	224.2229	220.8603	610.9754	417.7122	569.9043	231.5417	15.67901	532.864	101.8168	0.007149357	5.9752E-243	2.301374075
IMGA|Medtr7g102560.1	Mtr.43961.1.S1_at	33.3817	41.84205	60.25885	127.0619	202.5294	192.9085	45.16087	13.7425	174.1666	41.07651	0.006704187	1.91669E-59	3.856582258
	Mtr.44028.1.S1_at	53.86498	54.60366	53.49534	97.04707	89.69831	139.2569	53.988	0.564307	108.6674	26.74486	0.024005605	0	2.012807187
	Mtr.44069.1.S1_at	8.301508	12.01825	10.18506	45.365	78.58452	40.52661	10.16827	1.858425	54.82538	20.71775	0.020501251	0	5.391809299
IMGA|Medtr5g009970.2	Mtr.44072.1.S1_at	8.620812	9.989682	9.162348	30.37514	14.90726	39.20157	9.257614	0.68939	28.16132	12.29753	0.056488793	0	3.041963368
	Mtr.44094.1.S1_at	31.2422	41.03182	52.49288	108.2819	110.6158	94.84723	41.58896	10.63629	104.5816	8.510629	0.001317918	0	2.514648895
	Mtr.44270.1.S1_at	428.275	396.4642	230.9129	979.0004	679.5624	697.8713	351.884	105.9646	785.478	167.8451	0.019377262	1.36716E-12	2.232206972
IMGA|Medtr3g052760.1	Mtr.44271.1.S1_at	9.792328	12.91036	8.30109	22.62968	50.69832	20.88987	10.33459	2.351997	31.40596	16.7303	0.09686433	2.65289E-54	3.038915318
IMGA|Medtr5g026370.1	Mtr.44280.1.S1_at	9.807027	10.16122	8.258097	31.71203	30.05482	30.10397	9.408782	1.012137	30.62361	0.94292	1.19378E-05	0	3.254789586
IMGA|Medtr1g110510.1	Mtr.44300.1.S1_at	23.01614	61.32222	43.68775	64.84744	177.3558	170.0048	42.67537	19.1731	137.4027	62.94209	0.067225187	1.15409E-17	3.219718493
IMGA|AC146712_4.1	Mtr.44326.1.S1_at	13.11922	10.68161	9.995406	71.8119	81.47757	77.69489	11.26541	1.641696	76.99479	4.87072	2.45939E-05	0	6.834617885
IMGA|Medtr8g046210.1	Mtr.44354.1.S1_at	7.665786	9.279437	13.81991	25.25278	19.82167	16.90922	10.25505	3.190953	20.66122	4.234665	0.027295566	1.6189E-08	2.014737429
IMGA|Medtr5g019580.1	Mtr.4441.1.S1_at	71.81594	49.90023	115.7325	170.7384	170.865	177.8232	79.14957	33.52326	173.1422	4.054355	0.008515601	0	2.187531663
	Mtr.44416.1.S1_at	10.9402	13.38551	12.17507	33.58055	23.78544	31.61381	12.16693	1.222679	29.65993	5.181628	0.00470852	1.4693E-135	2.437750709
IMGA|Medtr3g104780.1	Mtr.44462.1.S1_at	26.1268	22.69494	17.30601	77.58166	39.14787	48.56364	22.04258	4.446435	55.09772	20.03272	0.049307885	6.12961E-38	2.499603941
IMGA|AC233572_25.1	Mtr.44504.1.S1_at	11.48102	13.05673	17.53303	53.64684	88.50648	69.86498	14.02359	3.139716	70.67277	17.44385	0.0052044	2.1708E-214	5.039562714
	Mtr.44516.1.S1_at	116.6709	183.9894	208.4954	654.977	412.0316	553.0219	169.7185	47.54656	540.0102	121.9942	0.008053017	1.81111E-41	3.181798442
IMGA|Medtr3g080860.1	Mtr.44658.1.S1_at	17.74844	11.79972	16.72876	26.21774	180.2595	38.23118	15.42564	3.181261	81.56947	85.67887	0.252427118	5.3967E-284	5.287914058
IMGA|Medtr5g023260.1	Mtr.44713.1.S1_at	20.18425	23.27258	27.3806	56.69831	48.87836	47.19832	23.61248	3.610199	50.925	5.069911	0.001607682	0	2.156698707
IMGA|Medtr7g070050.1	Mtr.45080.1.S1_at	239.043	225.1983	209.8468	666.7109	549.6315	600.2652	224.696	14.60455	605.5359	58.71739	0.000401947	0	2.694911223
	Mtr.45103.1.S1_at	6.122785	4.748973	5.899594	13.70045	18.97218	20.26995	5.590451	0.737236	17.64753	3.479313	0.004201745	1.7005E-176	3.15672678
	Mtr.45188.1.S1_at	8.281545	9.151173	7.251129	30.37514	19.51404	16.33336	8.227949	0.951155	22.07418	7.362651	0.031961696	2.8816E-140	2.682829058
IMGA|Medtr3g111120.1	Mtr.45211.1.S1_at	25.17006	28.21925	23.58859	61.86498	46.16744	92.03941	25.6593	2.353776	66.69061	23.31361	0.038668757	2.9136E-200	2.59908163
	Mtr.4531.1.S1_s_at	3.940089	4.83746	4.274285	4.916738	32.85405	4.961991	4.350611	0.453528	14.24426	16.11656	0.347757588	0	3.274081941
IMGA|Medtr7g084750.1	Mtr.45463.1.S1_at	11.63247	16.88078	25.00606	34.70526	37.88945	57.08492	17.83977	6.738173	43.22655	12.10684	0.033743722	6.76941E-11	2.423043987
	Mtr.4561.1.S1_at	7.613411	6.664411	5.325321	16.2428	13.17115	21.39878	6.534381	1.149574	16.93758	4.157584	0.013949409	2.2777E-55	2.592070567
IMGA|Medtr5g030030.1	Mtr.45653.1.S1_at	36.80043	47.77051	44.56378	146.7017	91.46376	244.365	43.0449	5.64056	160.8435	77.42537	0.058292499	1.6503E-286	3.736644181
IMGA|Medtr7g016820.1	Mtr.45667.1.S1_x_at	38.09975	24.98497	24.0582	196.5707	205.5316	182.6983	29.04764	7.853038	194.9336	11.50437	3.26282E-05	0	6.710822561
IMGA|Medtr4g100590.2	Mtr.45897.1.S1_at	27.80086	29.67756	29.54418	87.19343	79.59264	70.25424	29.00753	1.047134	79.01344	8.484438	0.000534262	0	2.723893612
IMGA|Medtr1g031640.1	Mtr.45999.1.S1_at	51.65097	105.4338	37.14628	134.78	139.2417	191.4162	64.74367	35.97721	155.1459	31.49009	0.03064632	6.61294E-07	2.396310429
IMGA|Medtr1g015780.1	Mtr.46412.1.S1_at	9.35304	19.79126	20.16232	77.36498	60.07204	146.9722	16.43554	6.136431	94.80308	45.99974	0.043032288	2.0397E-108	5.768175124
IMGA|Medtr8g087410.1	Mtr.46485.1.S1_at	11.31471	15.4453	15.40186	29.80803	25.96088	37.48863	14.05395	2.372357	31.08586	5.869146	0.00958948	1.69032E-35	2.211893004
	Mtr.4652.1.S1_at	169.5819	155.1949	148.7147	453.6983	408.741	518.1919	157.8305	10.68031	460.2104	55.01525	0.000729992	0	2.915852336
IMGA|Medtr4g013730.1	Mtr.4663.1.S1_at	8.095598	5.622203	10.39645	20.11067	14.39201	24.51298	8.038083	2.387643	19.67188	5.074729	0.022902334	3.19029E-17	2.447335206
IMGA|Medtr3g072170.1	Mtr.46631.1.S1_at	7.484424	7.254832	8.84076	8.773184	158.277	10.24922	7.860005	0.857081	59.09979	85.89312	0.359879461	0	7.519051726
IMGA|Medtr1g084010.1	Mtr.46919.1.S1_at	11.6676	10.24068	10.16686	18.70559	21.29611	25.31616	10.69171	0.845951	21.77262	3.330942	0.005041986	6.0644E-114	2.036401589
IMGA|Medtr7g071970.1	Mtr.47022.1.S1_s_at	4077.795	2698.282	3534.468	8437.774	7408.05	9098.777	3436.848	694.9183	8314.867	852.0382	0.001542394	0	2.419329115
IMGA|Medtr3g009030.1	Mtr.47087.1.S1_at	50.23961	52.0473	77.547	137.363	98.82146	125.4257	59.94464	15.27087	120.5367	19.73043	0.013625501	6.30996E-12	2.010801011
IMGA|Medtr7g088790.1	Mtr.4716.1.S1_at	7.151972	8.606409	13.13902	16.70686	36.03097	28.79215	9.632467	3.122624	27.17666	9.762821	0.041364617	2.21644E-22	2.821360019
IMGA|Medtr7g023730.1	Mtr.47292.1.S1_at	18.10219	20.47911	23.88528	38.00925	59.28692	55.31043	20.82219	2.906772	50.86887	11.31284	0.01119855	1.10205E-71	2.443012125
IMGA|Medtr7g023670.1	Mtr.47295.1.S1_x_at	90.20523	117.0947	175.2911	340.0764	678.5906	717.3542	127.5303	43.49225	578.6737	207.5384	0.021110092	3.56561E-72	4.537537889
IMGA|Medtr4g095450.2	Mtr.47795.1.S1_at	1093.304	1256.593	2060.579	3564.121	3521.691	4255.05	1470.159	517.7959	3780.287	411.7038	0.003769437	0	2.571346597
IMGA|Medtr2g007960.1	Mtr.47908.1.S1_at	66.01823	134.132	81.09146	189.1262	192.1004	258.404	93.74724	35.77708	213.2102	39.1672	0.017533211	7.31719E-09	2.274309299
IMGA|Medtr3g085640.1	Mtr.4792.1.S1_at	66.67605	82.77017	75.09088	136.4342	145.2876	186.0343	74.8457	8.04986	155.9187	26.45386	0.007089181	3.81773E-68	2.083201649
IMGA|Medtr3g109430.1	Mtr.47992.1.S1_at	10.52685	13.38957	19.90459	43.42733	39.11689	61.77214	14.607	4.805943	48.10545	12.03033	0.011000494	1.4714E-33	3.293314146
IMGA|Medtr3g106060.1	Mtr.48109.1.S1_at	8.738744	10.31104	11.15243	68.88729	21.68102	17.641	10.0674	1.225147	36.06977	28.49251	0.18943242	8.0393E-296	3.582827567
IMGA|Medtr2g007560.1	Mtr.48534.1.S1_at	39.98845	56.13098	69.69831	197.3423	134.0316	182.6983	55.27258	14.87352	171.3574	33.14397	0.005208613	1.21953E-41	3.100224952
IMGA|Medtr3g062600.1	Mtr.48713.1.S1_at	91.64131	88.83108	127.1785	213.8037	469.3903	239.8195	102.5503	21.37487	307.6712	140.6557	0.066965732	4.88016E-62	3.000197893
IMGA|Medtr4g106860.1	Mtr.48768.1.S1_at	2274.547	2657.285	2083.41	13433.23	12147.61	14895.29	2338.414	292.2196	13492.05	1374.779	0.000162326	0	5.769741636

IMGA|Medtr1g072420.1	Mtr.48829.1.S1_at	18.18719	23.94432	17.96344	38.40172	46.33669	54.53558	20.03165	3.390317	46.42467	8.067288	0.006409813	1.95156E-41	2.317565603
IMGA|Medtr1g014090.1	Mtr.48925.1.S1_at	288.5238	283.9488	422.4604	834.7424	714.0648	774.7427	331.6443	78.68233	774.5167	60.33908	0.001503697	0	2.335383429
IMGA|Medtr5g006450.1	Mtr.49090.1.S1_at	32.95877	42.922	102.6983	246.5021	179.3908	234.3999	59.52636	37.71843	220.0976	35.76867	0.005884394	7.54952E-15	3.697481081
IMGA|Medtr2g025120.1	Mtr.49441.1.S1_at	902.7466	1225.78	1281.666	1946.892	2167.008	2767.032	1136.731	204.5537	2293.644	424.4817	0.013130392	1.16991E-22	2.017755063
IMGA|Medtr6g023340.1	Mtr.49448.1.S1_at	407.6983	661.0738	561.365	1394.466	1347.992	2540.733	543.379	127.6417	1761.064	675.6131	0.037384681	2.48417E-61	3.24094886
IMGA|Medtr5g025150.1	Mtr.49487.1.S1_at	298.0625	329.1328	383.1686	1404.698	1013.532	1406.198	336.788	43.06637	1274.809	226.2745	0.002130322	0	3.785199163
IMGA|Medtr4g086330.1	Mtr.49557.1.S1_at	14.31372	16.26546	12.24727	50.77037	36.18836	65.78064	14.27548	2.009363	50.91312	14.79666	0.013161398	6.7285E-219	3.566472846
IMGA|Medtr6g082650.1	Mtr.49702.1.S1_at	22.69327	27.06588	26.23778	64.79579	51.30048	73.70714	25.33231	2.322675	63.26781	11.28121	0.004667632	4.739E-176	2.49751434
IMGA|Medtr8g013610.1	Mtr.50504.1.S1_at	8.060394	10.06374	15.72871	22.51363	27.29971	25.23738	11.28428	3.97719	25.01691	2.400643	0.006885727	0	2.216969786
IMGA|Medtr5g014150.1	Mtr.50780.1.S1_at	13.48292	13.42574	13.75675	37.77787	39.94243	57.53165	13.55514	0.176926	45.08398	10.83419	0.007283028	0	3.325970507
IMGA|Medtr5g017210.1	Mtr.50930.1.S1_at	81.04246	50.87857	52.0542	278.3898	189.4677	207.298	61.32508	17.08587	225.0519	47.04448	0.004784937	7.26299E-62	3.669817692
IMGA|Medtr8g104490.2	Mtr.51042.1.S1_at	39.86525	178.1148	126.9756	689.785	172.6829	230.4845	114.9852	69.90036	364.3175	283.3409	0.213027037	6.48495E-10	3.16838601
IMGA|Medtr4g108150.1	Mtr.51082.1.S1_at	33.25015	19.80651	40.71691	116.5314	142.2137	165.3545	31.25786	10.59661	141.3666	24.42259	0.002010004	2.03374E-72	4.522592774
IMGA|Medtr7g086320.1	Mtr.51154.1.S1_at	7.316809	8.214612	5.979902	6.769119	113.2136	11.30335	7.170441	1.124522	43.76203	60.18955	0.351827473	0	6.103115229
IMGA|Medtr5g030920.1	Mtr.51192.1.S1_at	21.20955	16.94887	16.67692	56.1498	43.36505	61.73137	18.27844	2.54205	53.74874	9.415641	0.003245677	4.84E-129	2.940553291
IMGA|Medtr3g019080.1	Mtr.51222.1.S1_at	7.403398	14.99804	8.414724	44.95856	20.28378	31.3841	10.27205	4.123941	32.20881	12.35805	0.043396755	3.15958E-20	3.13557715
IMGA|Medtr2g062600.1	Mtr.51360.1.S1_at	52.35269	54.85605	64.09466	78.29673	245.4568	299.3224	57.10113	6.184559	207.692	115.2507	0.086689039	0	3.637265268
	Mtr.51374.1.S1_at	31.81327	28.09249	38.03986	43.52045	218.8634	53.44563	32.64854	5.026013	105.2765	98.49423	0.271164689	2.9703E-138	3.224538681
IMGA|Medtr3g096910.1	Mtr.51564.1.S1_at	129.6921	109.7717	66.35806	252.591	195.9044	269.1279	101.9406	32.3851	239.2078	38.40248	0.009085047	2.11292E-13	2.346539847
IMGA|Medtr2g099630.1	Mtr.51785.1.S1_at	19.45948	22.80882	15.1517	32.32797	38.56075	49.27236	19.14	3.838543	40.05369	8.570285	0.018187311	3.84395E-21	2.092669661
IMGA|Medtr4g106590.1	Mtr.51829.1.S1_at	29.55289	27.98585	40.63237	154.5718	153.865	118.1566	32.72371	6.893776	142.1978	20.82327	0.000984936	0	4.345406905
IMGA|Medtr4g106590.1	Mtr.51829.1.S1_s_at	264.7506	281.0316	379.5316	1528.698	1138.365	1092.088	308.438	62.10475	1253.05	239.8369	0.002724562	5.9388E-153	4.062568338
IMGA|Medtr7g081500.1	Mtr.51889.1.S1_s_at	451.3138	574.1983	409.6222	987.1799	1065.46	1228.559	478.3781	85.56097	1093.733	123.1481	0.002070112	0	2.286335361
IMGA|Medtr5g037700.1	Mtr.51997.1.S1_at	5.728851	9.140906	7.766433	16.3352	24.4608	20.51845	7.545397	1.716733	20.43815	4.063395	0.007168662	1.10704E-38	2.708690729
IMGA|Medtr4g094240.1	Mtr.52124.1.S1_s_at	4322.687	3914.796	4267.3	8699.811	7855.746	9799.498	4168.261	221.247	8785.018	974.6734	0.001323453	4.8511E-286	2.107597785
IMGA|Medtr3g087330.1	Mtr.52244.1.S1_at	7.760182	10.23911	7.482159	15.88736	20.30787	21.28441	8.493816	1.517845	19.15988	2.875839	0.004738296	4.42835E-34	2.255744817
IMGA|Medtr1g098550.1	Mtr.52248.1.S1_at	123.0766	117.3168	134.0882	275.1646	294.2232	277.9831	124.8272	8.521636	282.457	10.28683	3.38397E-05	0	2.262783599
	Mtr.5260.1.S1_at	23.6076	20.92261	41.12926	296.4368	108.6449	220.2508	28.55316	10.97365	208.4441	94.45103	0.030592295	2.433E-177	7.300213362
	Mtr.5335.1.S1_at	55.601	61.06067	63.81135	151.8108	122.2132	108.0856	60.15767	4.178995	127.3699	22.31403	0.006848175	8.8331E-171	2.11726729
IMGA|Medtr4g106590.1	Mtr.5343.1.S1_s_at	78.67955	73.70312	99.36498	443.5914	337.3596	304.7114	83.91588	13.60872	361.8874	72.61631	0.002862529	3.6111E-274	4.312502292
IMGA|Medtr5g055680.1	Mtr.5486.1.S1_s_at	7.942212	5.403197	8.479815	16.21295	31.37208	30.84845	7.275075	1.643228	26.14449	8.604952	0.02028411	5.05302E-88	3.59370795
IMGA|Medtr4g101280.1	Mtr.5687.1.S1_s_at	10.43629	10.49112	10.95158	25.80796	48.12715	103.3614	10.62633	0.283009	59.09882	39.92387	0.103318064	0	5.561546545
IMGA|Medtr2g010470.1	Mtr.5765.1.S1_at	11.91915	11.35792	19.10784	45.365	45.29395	37.27836	14.1283	4.321528	42.64577	4.648449	0.001470172	0	3.018463456
IMGA|Medtr3g108240.3	Mtr.5950.1.S1_s_at	343.8815	338.1499	388.3276	897.0316	712.2273	1023.346	356.7863	27.46544	877.5349	156.4729	0.004749348	1.5865E-236	2.459553035
IMGA|Medtr4g027240.1	Mtr.6484.1.S1_at	61.19831	62.00213	92.35073	354.6676	202.8288	275.0853	71.85039	17.75836	277.5272	75.94886	0.010281253	1.63229E-89	3.862571143
IMGA|Medtr1g090150.1	Mtr.6489.1.S1_at	10.29517	14.34987	11.32936	58.23934	35.00388	25.14434	11.99146	2.106878	39.46252	16.99204	0.049873094	6.2795E-113	3.290884293
	Mtr.6543.1.S1_at	10.42107	13.76917	15.67601	74.12879	39.83612	51.51326	13.28875	2.660207	55.15939	17.43466	0.014707423	1.2079E-163	4.150833833
	Mtr.6645.1.S1_at	9.722878	13.4401	12.70764	277.5291	29.09417	58.29553	11.95687	1.969053	121.6396	135.7915	0.234410661	0	10.17319672
IMGA|Medtr8g046210.1	Mtr.6667.1.S1_at	19.58937	16.53867	43.99647	97.71165	75.03165	82.09714	26.70817	15.0496	84.94681	11.60543	0.00605531	0	3.180555301
IMGA|Medtr3g102020.1	Mtr.7249.1.S1_at	24.28662	24.03334	31.2437	51.85043	71.69831	52.40057	26.52122	4.09175	58.64977	11.30372	0.009814101	3.99933E-42	2.211427993
	Mtr.7260.1.S1_at	67.77453	92.26848	125.2592	340.4898	416.021	393.3432	95.10075	28.84683	383.2847	38.75722	0.000495312	0	4.030301061
IMGA|Medtr4g027040.1	Mtr.7267.1.S1_at	9.430828	6.440316	11.71853	17.91327	21.16725	17.64537	9.196557	2.646892	18.90863	1.960604	0.006949126	0	2.056055219
	Mtr.7268.1.S1_at	6.400543	7.572959	8.129459	15.70202	107.0723	26.02194	7.367654	0.882553	49.59874	50.04028	0.21767723	0	6.731959125
IMGA|Medtr7g085810.1	Mtr.7556.1.S1_at	8.170352	6.542034	21.6205	30.02345	31.5099	94.65201	12.11096	8.275645	52.06179	36.89171	0.141196051	6.19244E-17	4.298733087
	Mtr.7658.1.S1_at	285.5908	255.4359	293.9925	729.5802	600.8478	690.3977	278.3397	20.27523	673.6085	65.98798	0.000580332	0	2.420094895
	Mtr.7741.1.S1_at	24.07138	37.4252	30.20885	124.6231	100.48	330.5904	30.56848	6.684168	185.2312	126.4622	0.101875837	0	6.05954779
	Mtr.7854.1.S1_at	277.2884	238.994	394.4019	693.3639	625.3889	780.3285	303.5614	80.96673	699.6938	77.66353	0.003619961	0	2.304949481
IMGA|Medtr1g025340.1	Mtr.7906.1.S1_at	44.81952	42.35919	61.25663	289.865	109.0093	187.3034	49.47845	10.27412	195.3926	90.69878	0.050394141	1.3026E-133	3.949044042
IMGA|Medtr5g018750.1	Mtr.81.1.S1_at	116.8554	108.809	85.04682	259.5914	305.6341	352.6763	103.5704	16.53868	305.9673	46.54337	0.002081642	1.0293E-99	2.954196405
IMGA|Medtr1g022970.1	Mtr.8179.1.S1_at	7.933874	13.23795	7.274796	26.65117	33.53496	27.42184	9.482207	3.269221	29.20266	3.771619	0.002385959	0	3.079732176

IMGA|Medtr5g018770.1	Mtr.82.1.S1_at	327.022	365.8288	379.578	818.4424	906.1531	810.3203	357.4763	27.25538	844.9719	53.13984	0.000145282	0	2.363714664
IMGA|Medtr5g017850.1	Mtr.8319.1.S1_at	14.81921	28.5613	12.68629	90.46798	104.9754	125.9594	18.68893	8.615981	107.1343	17.84392	0.001507465	0	5.732497328
	Mtr.8398.1.S1_at	13.66656	12.81257	10.26609	79.7244	25.08959	22.5871	12.24841	1.76904	42.46703	32.29008	0.180863851	2.2308E-192	3.467146805
	Mtr.8442.1.S1_at	265.081	327.5878	253.4036	684.5738	641.4209	610.1234	282.0242	39.8889	645.3727	37.38218	0.000325087	0	2.288359787
IMGA|Medtr1g072610.3	Mtr.8458.1.S1_at	8.84559	9.577211	8.040924	15.12919	22.03211	20.73177	8.821242	0.768433	19.29769	3.668107	0.008389179	2.857E-123	2.187638475
	Mtr.8460.1.S1_s_at	10.44179	8.263957	8.220572	26.31267	23.61648	38.08934	8.97544	1.270082	29.3395	7.696566	0.010645174	9.8415E-170	3.268864675
	Mtr.8498.1.S1_at	356.8033	192.8762	298.5866	2610.886	2769.421	4515.293	282.7554	83.10233	3298.533	1056.722	0.007884769	0	11.6656792
	Mtr.8498.1.S1_s_at	145.8853	84.20551	120.9474	1006.466	900.0581	2277.881	117.0127	31.02756	1394.802	766.6176	0.044805373	0	11.92008527
	Mtr.8507.1.S1_at	91.69622	56.77539	59.1071	1558.017	697.149	755.4364	69.1929	19.52329	1003.534	481.0801	0.028273933	0	14.5034278
IMGA|Medtr7g050980.1	Mtr.8508.1.S1_at	51.5324	75.72769	71.18557	274.0627	260.5934	394.3222	66.14855	12.86008	309.6595	73.62876	0.004855828	6.4793E-236	4.681273623
IMGA|Medtr7g093530.1	Mtr.8522.1.S1_at	16.93881	37.54964	18.92185	91.07547	46.8515	134.0137	24.4701	11.37053	90.6469	43.58269	0.063655836	6.73262E-24	3.704394063
IMGA|Medtr1g115820.1	Mtr.8531.1.S1_at	880.1216	823.599	1207.354	2332.17	1889.84	1908.598	970.3581	207.1808	2043.536	250.1404	0.004613932	2.91525E-19	2.105960957
IMGA|Medtr1g090060.1	Mtr.8546.1.S1_at	254.6983	311.2594	271.7594	1712.942	3137.41	1768.97	279.239	29.01287	2206.441	806.7301	0.014434695	0	7.901619963
IMGA|Medtr3g082100.1	Mtr.8548.1.S1_at	1064.453	1508.546	1632.274	3507.561	5419.888	3975.195	1401.757	298.5936	4300.882	996.8965	0.008490453	1.83392E-63	3.068206613
IMGA|Medtr5g094210.1	Mtr.8557.1.S1_s_at	317.155	401.2225	235.8167	942.7868	1059.663	691.9573	318.0647	82.70664	898.1358	187.8755	0.008075952	5.8853E-34	2.823751801
	Mtr.8558.1.S1_at	6.938022	9.940736	7.267377	19.15503	35.87714	46.87129	8.048712	1.646796	33.96782	13.95643	0.033073622	1.2436E-163	4.220280099
	Mtr.8559.1.S1_at	336.5237	469.865	318.7373	760.7342	996.0316	824.0256	375.042	82.59927	860.2638	121.7626	0.004646253	2.56998E-24	2.293779975
IMGA|AC235753_8.1	Mtr.8574.1.S1_at	119.688	195.4856	197.4478	614.6219	1297.145	684.9766	170.8738	44.33905	865.5811	375.397	0.033432894	3.5186E-162	5.065615557
	Mtr.8601.1.S1_at	29.76819	50.34174	44.22215	208.6983	165.5316	230.8148	41.44403	10.56438	201.6816	33.20239	0.001345814	0	4.866360824
IMGA|Medtr7g011990.1	Mtr.8606.1.S1_at	10.28534	11.87128	9.796098	15.30841	43.90131	40.9261	10.65091	1.084818	33.37861	15.7198	0.066890906	2.6243E-288	3.13387484
IMGA|Medtr8g005420.1	Mtr.8617.1.S1_at	351.1317	506.5787	454.396	1641.311	890.3905	1601.134	437.3688	79.10993	1377.612	422.4242	0.019279386	3.67164E-94	3.149772241
IMGA|Medtr8g037800.1	Mtr.8630.1.S1_at	13.12561	45.36235	11.13705	166.8278	140.1983	292.5316	23.20834	19.21169	199.8526	81.35931	0.021582011	4.21247E-57	8.611242283
	Mtr.8652.1.S1_at	79.86498	61.40565	121.0031	352.0316	358.6893	343.0356	87.42458	30.50942	351.2522	7.855895	0.000131367	0	4.017773697
	Mtr.8654.1.S1_at	37.80458	29.32334	60.4905	100.7778	205.2669	210.0887	42.53947	16.11404	172.0445	61.76584	0.024583146	4.78203E-44	4.044348663
IMGA|Medtr4g093580.2	Mtr.8656.1.S1_s_at	100.1786	129.4251	188.0355	493.4244	231.2118	369.183	139.2131	44.73879	364.6064	131.1662	0.047975736	2.63732E-18	2.619053046
IMGA|AC235753_1.1	Mtr.8672.1.S1_at	137.7904	125.0266	117.3723	927.2705	612.7029	1008.408	126.7298	10.31508	849.4603	209.0129	0.00392587	0	6.702926235
	Mtr.8779.1.S1_at	7.820858	8.973592	8.365531	93.45643	132.5257	165.0351	8.38666	0.576657	130.3391	35.83938	0.004146897	0	15.54123519
	Mtr.8789.1.S1_s_at	245.5675	256.9889	285.5986	483.2173	623.865	778.1796	262.7183	20.62142	628.4206	147.5339	0.013136913	3.4427E-207	2.391993847
IMGA|Medtr1g023260.2	Mtr.8813.1.S1_at	268.8431	233.8722	301.9419	632.4879	555.8213	651.2386	268.2191	34.03913	613.1826	50.55329	0.00060678	0	2.286125842
	Mtr.8827.1.S1_at	9.149926	6.147004	6.950499	16.58721	20.04867	21.02146	7.41581	1.554597	19.21911	2.330617	0.001874864	0	2.591640274
	Mtr.8884.1.S1_at	23.29658	20.55645	17.95419	33.37287	48.96505	44.13684	20.6024	2.671488	42.15825	7.982176	0.011372837	2.19655E-44	2.046278239
IMGA|Medtr6g008500.1	Mtr.8948.1.S1_at	247.5158	249.7591	642.7574	796.125	693.741	957.8748	380.0108	227.548	815.9136	133.1741	0.045761844	1.4337E-08	2.147080335
	Mtr.8987.1.S1_at	5.737196	6.681267	6.681267	17.79767	22.6444	31.55119	6.366577	0.54506	23.99776	6.975924	0.012021206	0	3.769334174
IMGA|Medtr8g107620.1	Mtr.8993.1.S1_at	3256.752	3194.163	2033.365	6264.734	5641.038	6668.36	2828.093	688.9661	6191.378	517.5746	0.002497325	0	2.189240819
	Mtr.9079.1.S1_at	43.9991	70.81627	57.89977	133.0553	93.00046	183.9882	57.57171	13.41159	136.6813	45.60212	0.044893581	1.66977E-24	2.37410533
IMGA|Medtr5g020020.2	Mtr.9112.1.S1_at	129.365	121.9365	107.1353	725.9426	627.0339	636.6251	119.4789	11.31676	663.2005	54.54746	7.17861E-05	0	5.550774112
IMGA|Medtr3g087270.1	Mtr.9232.1.S1_at	23.76237	45.80821	16.47421	103.3045	231.6983	230.6774	28.6816	15.27318	188.5601	73.83527	0.021339919	1.81519E-73	6.57425358
IMGA|Medtr5g098720.1	Mtr.9254.1.S1_at	34.70051	50.47077	47.96248	516.9861	185.4617	232.7667	44.37792	8.474201	311.7382	179.3167	0.061353372	0	7.024623816
IMGA|Medtr3g031140.1	Mtr.9319.1.S1_at	87.36498	91.77647	96.53165	481.3862	449.6989	350.0266	91.89103	4.584407	427.0372	68.54921	0.001074862	0	4.647213318
	Mtr.9372.1.S1_at	7.943718	6.356757	17.24871	84.51795	39.34215	49.69355	10.51639	5.884099	57.85122	23.66693	0.028256901	3.96307E-44	5.501051089
	Mtr.9382.1.S1_at	87.87424	75.63939	65.27512	154.8679	134.3525	185.8372	76.26292	11.31246	158.3525	25.91867	0.007345227	3.13676E-36	2.076402701
IMGA|Medtr1g086790.1	Mtr.9388.1.S1_at	138.543	134.9816	122.1029	349.7862	262.6812	371.6244	131.8758	8.648889	328.0306	57.63794	0.004314683	0	2.487420128
	Mtr.9496.1.S1_at	75.21352	66.55395	81.46458	143.993	226.0147	179.0157	74.41068	7.487664	183.0078	41.15634	0.010851902	2.9525E-139	2.459429078
IMGA|Medtr2g030530.1	Mtr.9568.1.S1_at	42.70817	41.03182	33.87968	266.5542	57.03165	94.68098	39.20655	4.688737	139.4223	111.6972	0.195463581	5.3995E-300	3.556096142
	Mtr.9637.1.S1_at	6.858476	6.073749	6.509731	45.16678	14.04084	13.34335	6.480652	0.393171	24.18365	18.17526	0.16694728	0	3.731669797
IMGA|Medtr5g073210.1	Mtr.9645.1.S1_at	17.80764	29.42128	19.81601	34.9405	50.96937	54.23935	22.34831	6.207137	46.71641	10.32846	0.02483764	1.04828E-11	2.090377664
IMGA|Medtr5g036480.1	Mtr.9656.1.S1_at	20.5763	32.3699	36.88235	56.65317	77.5286	89.14693	29.94285	8.419605	74.4429	16.46518	0.014055476	5.46673E-20	2.486166429
	Mtr.9658.1.S1_at	184.9018	230.6183	372.2137	697.5797	688.5955	724.7051	262.5779	97.66014	703.6268	18.79899	0.001544859	0	2.679687577
	Mtr.9830.1.S1_at	253.7018	233.6748	266.9418	708.865	777.0013	666.3234	251.4395	16.74846	717.3965	55.82998	0.000157721	0	2.853158238
	Mtr.9846.1.S1_at	48.54425	49.25989	61.80792	104.9671	79.56096	143.0468	53.20402	7.459785	109.1916	31.95306	0.041744847	1.23187E-38	2.052318665

	Mtr.9931.1.S1_at	105.5367	135.6464	77.92743	205.9023	250.0177	361.4238	106.3702	28.8685	272.4479	80.15031	0.027872935	2.18355E-23	2.561318903
	Msa.1096.1.S1_at	253.6983	296.1983	253.7851	150.2869	72.8758	168.1906	267.8939	24.51236	130.4511	50.65887	0.013369416	0	0.486950575
	Msa.1101.1.S1_at	102.4417	133.0316	201.6983	56.05729	44.36502	27.10201	145.7239	50.83097	42.50811	14.56668	0.027760813	1.26684E-34	0.291703075
	Msa.1286.1.S1_at	1186.83	1265.246	1590.1	653.8423	550.494	541.8802	1347.392	213.8168	582.0722	62.30381	0.003998371	1.896E-100	0.43199905
IMGA|Medtr7g011060.2	Msa.1315.1.S1_at	622.3058	686.4042	631.7749	196.0894	234.5847	158.1154	646.8283	34.59919	196.2632	38.23492	0.000111118	0	0.303423928
IMGA|Medtr6g012500.1	Msa.1357.1.S1_at	585.8006	605.1881	812.4461	148.4067	190.3368	93.4012	667.8116	125.6317	144.0483	48.61457	0.002533231	1.03261E-77	0.215701943
	Msa.1378.1.S1_at	215.0998	149.9851	170.5587	81.03173	57.7611	95.45392	178.5479	33.28441	78.08225	19.01872	0.010503122	5.72046E-20	0.437318295
IMGA|Medtr5g010150.1	Msa.1445.1.S1_at	194.3631	206.865	226.1301	68.09966	112.3724	93.91798	209.1194	16.00304	91.46336	22.23823	0.001744655	0	0.437373846
IMGA|Medtr4g097700.1	Msa.1752.1.S1_s_at	413.127	363.7359	404.9931	212.2676	240.4812	103.8884	393.952	26.48204	185.5457	72.11065	0.009315748	0	0.470985628
	Msa.1860.1.S1_at	120.4524	126.865	208.3629	64.36498	52.45848	48.27993	151.8934	49.009	55.03446	8.346194	0.027925711	7.27165E-90	0.362322849
	Msa.1890.1.S1_at	112.1983	129.365	323.0316	12.59936	11.45783	10.60037	188.1983	117.0841	11.55252	1.002854	0.059229075	0	0.061384838
	Msa.1897.1.S1_at	30.29674	26.2383	58.36498	11.88868	8.526581	6.981003	38.30001	17.49486	9.132088	2.509243	0.046000404	3.74894E-90	0.238435681
IMGA|Medtr3g031650.1	Msa.2715.1.S1_at	3856.662	4029.975	4415.045	1472.022	1376.567	1922.031	4100.561	285.8055	1590.207	291.3048	0.00043948	0	0.387802354
	Msa.2788.1.S1_at	40.65151	43.20778	51.69831	15.47548	17.8226	14.71924	45.18587	5.782957	16.00577	1.618213	0.001091028	0	0.354220774
	Msa.2799.1.S1_at	654.9483	532.5316	586.2103	146.1983	120.9625	120.0316	591.2301	61.36252	129.0641	14.84593	0.000222814	0	0.218297649
IMGA|Medtr4g097700.1	Msa.3055.1.S1_at	2577.995	2377.72	2397.172	1266.707	1403.219	599.7578	2450.962	110.4428	1089.894	429.9237	0.006042334	0	0.444680215
	Msa.3080.1.S1_at	146.9959	132.3213	100.4696	71.75893	43.66689	47.19832	126.5956	23.78579	54.20805	15.30173	0.011394869	2.53164E-16	0.428198534
	Msa.3097.1.S1_at	837.4237	648.1933	870.8082	246.5064	139.2833	256.7805	785.475	120.0556	214.1901	65.07425	0.001925467	3.24518E-52	0.272688563
	Msa.925.1.S1_at	1125.495	1160.198	1510.154	541.865	715.652	588.3191	1265.283	212.7737	615.2787	89.97555	0.00819934	6.36001E-36	0.486277689
	Mtr.10131.1.S1_at	59.36498	92.50877	128.2676	27.70935	38.69873	42.80351	93.38045	34.45958	36.40386	7.804375	0.04915667	1.19229E-36	0.389844586
IMGA|Medtr5g021820.1	Mtr.10147.1.S1_at	86.30758	87.67469	88.73033	26.22608	31.22099	21.10578	87.57086	1.214706	26.18428	5.057735	3.3826E-05	0	0.299006783
	Mtr.10192.1.S1_at	285.0926	214.1641	266.4445	145.6875	117.365	89.53165	255.2337	36.76921	117.528	28.07829	0.006718998	1.98655E-17	0.460472278
	Mtr.10312.1.S1_at	185.865	228.06	59.97546	51.19811	58.93059	47.32549	157.9668	87.44617	52.48473	5.908564	0.105480292	6.1902E-210	0.332251613
IMGA|Medtr4g079160.1	Mtr.10331.1.S1_at	257.3632	338.9229	287.2913	45.48586	64.33581	46.54321	294.5258	41.25833	52.12163	10.591	0.000594285	0	0.17696794
IMGA|AC235757_49.1	Mtr.10452.1.S1_at	87.19976	723.664	181.1247	19.69661	120.8145	16.79287	330.6628	343.5737	52.43465	59.23645	0.239069623	4.10976E-16	0.158574363
IMGA|Medtr4g130510.1	Mtr.10615.1.S1_at	280.5803	227.2436	268.0235	104.0316	104.8336	119.1983	258.6158	27.88512	109.3545	8.534408	0.000894156	0	0.422845409
IMGA|Medtr5g045300.1	Mtr.10948.1.S1_at	263.0316	374.6983	85.65847	42.32542	60.02687	24.52143	241.1295	145.7593	42.29124	17.75275	0.078906496	7.76266E-84	0.175388101
IMGA|Medtr7g116510.2	Mtr.11001.1.S1_at	1144.498	698.3471	1246.985	309.1328	103.3244	295.516	1029.943	291.7069	235.9911	115.0943	0.01182668	6.63709E-33	0.229130129
	Mtr.11016.1.S1_at	187.5652	160.997	222.7061	84.36498	58.25051	91.81966	190.4228	30.95365	78.14505	17.62775	0.005472337	2.6761E-28	0.410376544
IMGA|Medtr5g091690.1	Mtr.11240.1.S1_at	515.1907	493.3589	581.8056	219.3746	270.1894	250.7928	530.1184	46.07419	246.7856	25.64333	0.000741705	0	0.465529205
	Mtr.11489.1.S1_at	340.811	333.2168	294.865	152.556	176.5851	133.1206	322.9643	24.62916	154.0872	21.77265	0.000881616	0	0.477103074
IMGA|Medtr8g076940.1	Mtr.11744.1.S1_at	58.64589	48.20463	47.72711	34.35467	18.40492	23.90069	51.52588	6.170732	25.55343	8.102302	0.011537369	3.0953E-13	0.495933891
	Mtr.11798.1.S1_at	1293.478	966.576	1017.709	455.5316	359.0966	495.865	1092.588	175.8448	436.8311	70.27575	0.003887573	9.33278E-59	0.399813318
	Mtr.11810.1.S1_at	763.7488	643.0666	560.811	305.824	371.8613	281.9462	655.8755	102.0735	319.8772	46.57579	0.006574693	7.94601E-36	0.487710194
	Mtr.11844.1.S1_at	286.5861	357.0679	261.5693	114.2897	123.653	128.2293	301.7411	49.52022	122.0573	7.105453	0.003399111	0	0.404510166
IMGA|Medtr2g028530.1	Mtr.1186.1.S1_s_at	940.2665	841.2642	502.0038	435.3703	394.3683	287.6074	761.1782	229.8453	372.4487	76.28116	0.049806142	1.07956E-18	0.489305457
IMGA|Medtr7g035200.1	Mtr.11999.1.S1_at	47.12731	57.42089	64.23656	27.87007	22.74681	27.63001	56.26158	8.613336	26.0823	2.891108	0.00452599	4.5628E-73	0.46358978
IMGA|Medtr6g069600.1	Mtr.12069.1.S1_at	943.6983	1200.695	1394.665	210.196	422.6983	137.1295	1179.686	226.2161	256.6746	148.3495	0.004104079	4.44034E-27	0.217578753
IMGA|Medtr3g031650.1	Mtr.12393.1.S1_at	9252.471	9291.395	10108.38	3905.198	3685.532	5190.188	9550.748	483.3142	4260.306	812.7566	0.000634683	0	0.446070366
IMGA|Medtr3g072500.1	Mtr.12531.1.S1_s_at	353.3166	465.874	443.1965	166.0064	219.7633	195.5874	420.7957	59.52849	193.7857	26.92372	0.003839666	2.65001E-48	0.460522018
IMGA|Medtr4g113650.1	Mtr.1260.1.S1_at	65.57635	60.03289	59.73813	23.69154	19.65271	24.28384	61.78246	3.288909	22.5427	2.52026	8.08704E-05	0	0.364872128
IMGA|Medtr3g080410.1	Mtr.12816.1.S1_at	131.3189	252.6218	280.9742	72.48508	85.01409	85.31655	221.6383	79.49315	80.93857	7.322502	0.037926617	7.3144E-243	0.365183157
	Mtr.12848.1.S1_at	219.643	351.436	294.5703	99.38265	135.2324	107.7392	288.5498	66.10241	114.1181	18.75681	0.011718837	2.26117E-58	0.395488367
IMGA|Medtr2g025740.1	Mtr.12962.1.S1_at	356.3775	432.9882	456.3183	153.6491	228.1983	189.4669	415.228	52.2839	190.4381	37.28411	0.003736403	1.58213E-25	0.458635017
	Mtr.13037.1.S1_at	265.9896	477.5316	508.7638	158.991	215.365	219.8616	417.4284	132.0762	198.0725	33.92022	0.049502868	4.03815E-29	0.474506609
	Mtr.13147.1.S1_at	332.1513	505.4894	537.3488	118.7996	273.2797	183.3418	458.3298	110.4288	191.807	77.58717	0.026768052	2.6841E-09	0.418491292
	Mtr.13545.1.S1_at	576.6541	678.8736	1108.865	74.53071	23.44978	20.95098	788.1309	282.4269	39.64382	30.23875	0.010306049	0	0.050301066
	Mtr.13640.1.S1_at	102.2315	110.8905	114.865	32.3699	36.46549	34.43294	109.329	6.459885	34.42278	2.047817	4.38584E-05	0	0.314855028
IMGA|Medtr5g026350.1	Mtr.13758.1.S1_at	133.0316	116.8529	84.63337	45.49787	43.57564	33.91854	111.506	24.6382	40.99735	6.205311	0.008606538	3.16033E-86	0.367669478
IMGA|AC225529_15.1	Mtr.13825.1.S1_at	43.85804	43.18721	33.71574	24.03635	21.18519	14.89069	40.25366	5.671935	20.03741	4.679619	0.008892487	7.28897E-14	0.497778587

	Mtr.13898.1.S1_at	281.4565	268.2421	214.6789	127.0944	123.2691	119.4766	254.7925	35.3621	123.28	3.808886	0.003053007	0	0.483844819
IMGA|Medtr8g092320.1	Mtr.14131.1.S1_at	206.8137	179.9978	142.4396	26.2383	17.08997	27.84521	176.417	32.33605	23.72449	5.80157	0.001292704	0	0.134479597
IMGA|Medtr4g112350.1	Mtr.14236.1.S1_at	86.25832	137.3038	154.5924	50.62815	62.36498	60.43593	126.0515	35.52954	57.80969	6.293742	0.030622525	1.09502E-78	0.458619495
IMGA|Medtr6g072710.1	Mtr.14426.1.S1_at	71.68805	89.13259	102.6823	39.51044	43.3788	44.22096	87.83432	15.53786	42.37006	2.512051	0.007474185	1.0592E-215	0.482386214
IMGA|Medtr2g081300.1	Mtr.14435.1.S1_at	174.3456	129.365	79.70818	45.06011	46.50019	32.30704	127.8063	47.33797	41.28911	7.811958	0.035412327	5.19388E-82	0.323060163
IMGA|Medtr3g084250.1	Mtr.14486.1.S1_at	266.8257	274.365	294.5472	46.19833	49.3207	56.65271	278.5793	14.33322	50.72392	5.36659	1.34356E-05	0	0.182080708
IMGA|Medtr3g084220.1	Mtr.14493.1.S1_x_at	389.3112	409.9283	474.8539	150.6492	144.1364	151.5376	424.6978	44.64291	148.7744	4.041138	0.00043833	0	0.350306472
IMGA|Medtr5g022770.1	Mtr.14580.1.S1_at	82.03165	76.15099	48.19343	27.26499	32.01437	32.01437	68.79202	18.0796	30.43125	2.742059	0.022092047	1.0516E-129	0.442365907
IMGA|Medtr7g085150.1	Mtr.14668.1.S1_at	757.148	1143.147	1888.652	354.3691	276.2079	394.1553	1262.982	575.1918	341.5774	60.00514	0.050869859	7.4729E-156	0.270453047
IMGA|Medtr8g014330.1	Mtr.14743.1.S1_at	42.40145	56.4163	56.10745	22.3994	20.98977	17.95419	51.64173	8.003811	20.44779	2.271625	0.002899955	4.8473E-125	0.395954693
IMGA|Medtr3g093020.1	Mtr.14816.1.S1_at	13.58923	12.93919	20.91394	6.800607	9.142714	5.548303	15.81412	4.428517	7.163875	1.824533	0.035248513	2.18074E-16	0.453004923
IMGA|Medtr4g108100.1	Mtr.15618.1.S1_at	252.8434	352.0316	422.6101	106.5671	191.2751	164.7271	342.4951	85.28422	154.1897	43.32596	0.027038021	5.15548E-14	0.450195495
IMGA|Medtr2g034680.1	Mtr.15775.1.S1_at	1220.292	842.8174	925.979	430.8082	275.8713	454.8381	996.3628	198.336	387.1725	97.13565	0.008789816	1.73677E-27	0.38858588
IMGA|Medtr5g035150.1	Mtr.15901.1.S1_at	10.98317	15.33302	10.92429	6.5649	5.608835	5.723112	12.4135	2.528553	5.965616	0.522131	0.012393365	1.7721E-101	0.480575012
IMGA|Medtr4g128820.1	Mtr.16226.1.S1_at	38.37654	39.48374	29.34093	17.14126	18.91789	15.05246	35.73374	5.563942	17.03721	1.934814	0.005337509	7.0402E-63	0.476782077
IMGA|Medtr4g128770.1	Mtr.16234.1.S1_at	154.9417	92.23847	94.96132	39.94252	49.46319	44.06442	114.0472	35.44188	44.49004	4.774586	0.028073952	1.7481E-140	0.390102081
IMGA|Medtr4g106480.1	Mtr.17068.1.S1_at	59.04646	58.01553	47.2748	18.9033	14.36409	11.75902	54.77893	6.519179	15.0088	3.615515	0.00076246	0	0.273988626
IMGA|Medtr8g092340.1	Mtr.17470.1.S1_at	89.69702	86.3088	91.16801	11.77501	11.55108	10.5143	89.05795	2.491847	11.28013	0.672614	8.06483E-07	0	0.126660575
IMGA|Medtr2g027410.1	Mtr.17669.1.S1_at	16.66875	29.23742	42.95807	10.4312	6.626667	10.99204	29.62141	13.14886	9.349968	2.375061	0.058322987	1.88044E-49	0.315648961
IMGA|Medtr1g014120.1	Mtr.17895.1.S1_at	735.0766	1414.576	1524.631	319.9046	658.4699	475.1035	1224.761	427.6347	484.4927	169.4778	0.049443524	3.86402E-14	0.395581289
IMGA|Medtr6g012220.1	Mtr.18494.1.S1_at	296.6983	360.5141	163.2074	11.45783	11.13576	15.88931	273.4733	100.6828	12.82764	2.656377	0.010970139	0	0.046906359
IMGA|Medtr4g081390.1	Mtr.18524.1.S1_at	57.29717	33.21986	31.14551	16.6695	17.79767	14.75474	40.55418	14.5369	16.4073	1.538316	0.045878933	9.1131E-163	0.404577348
IMGA|Medtr8g088080.1	Mtr.18775.1.S1_s_at	10.05336	14.45963	66.11352	11.06902	7.287546	7.656095	30.20884	31.17232	8.670885	2.085	0.298423144	1.36459E-71	0.287031422
IMGA|Medtr3g086630.1	Mtr.19208.1.S1_at	93.7726	109.9765	56.76956	29.61658	33.57795	39.28264	86.83957	27.27262	34.15906	4.859163	0.030107874	1.14379E-78	0.393358245
IMGA|Medtr8g077830.1	Mtr.19622.1.S1_s_at	115.0897	179.9763	46.47147	41.42273	35.69979	28.74245	113.8458	66.76111	35.28832	6.350148	0.112348292	7.4635E-102	0.309965895
IMGA|Medtr2g083250.1	Mtr.19874.1.S1_at	242.4816	366.2348	207.1702	112.1578	108.448	109.9114	271.9622	83.52972	110.1724	1.868626	0.028463112	0	0.40510195
IMGA|Medtr4g072670.1	Mtr.19878.1.S1_x_at	207.1983	216.1611	218.4363	31.2798	34.5339	40.26053	213.9319	5.94139	35.35807	4.546737	2.04598E-06	0	0.16527724
IMGA|Medtr4g072650.1	Mtr.19879.1.S1_at	78.38246	73.01614	63.76599	17.84967	15.45383	30.18377	71.72153	7.393733	21.16242	7.904018	0.001268079	0	0.29506371
IMGA|Medtr4g072640.1	Mtr.19880.1.S1_at	129.1918	111.3993	92.94537	12.95294	9.201372	17.67123	111.1788	18.12423	13.27518	4.244111	0.000805403	0	0.11940385
IMGA|Medtr4g072620.1	Mtr.19881.1.S1_x_at	146.5804	106.1983	86.52612	56.9605	57.80248	38.60117	113.1016	30.61652	51.12138	10.85099	0.029795157	4.44803E-23	0.451995186
IMGA|Medtr4g072530.1	Mtr.19891.1.S1_s_at	196.186	147.1839	178.848	33.36835	21.20273	21.15575	174.0727	24.84763	25.24228	7.037421	0.000565964	0	0.145010014
IMGA|Medtr4g072450.1	Mtr.19898.1.S1_x_at	135.4134	119.0378	127.2782	21.76144	20.90513	35.09669	127.2431	8.187864	25.92108	7.957831	0.000104539	0	0.203713025
IMGA|Medtr4g072380.1	Mtr.19907.1.S1_at	61.82004	38.82697	36.80471	15.1594	17.88048	19.82984	45.81724	13.89567	17.62324	2.345826	0.025694269	3.03352E-96	0.384642132
IMGA|Medtr4g072310.1	Mtr.19917.1.S1_at	127.8349	114.7062	95.90417	40.39293	51.2571	38.25191	112.8151	16.04913	43.30064	6.973151	0.002337814	8.39794E-67	0.383819666
IMGA|Medtr4g072300.1	Mtr.19918.1.S1_at	1300.306	1183.93	895.7095	321.4296	354.428	298.9119	1126.649	208.2917	324.9231	27.92243	0.002718938	0	0.288397949
IMGA|Medtr4g072300.1	Mtr.19918.1.S1_x_at	1353.799	1296.093	977.7218	348.5831	347.3436	312.7987	1209.205	202.5357	336.2418	20.3118	0.001753449	0	0.278068582
IMGA|Medtr4g072270.1	Mtr.19925.1.S1_x_at	172.168	176.365	159.0316	53.65425	56.6552	58.72902	169.1882	9.0427	56.34616	2.551463	3.15567E-05	0	0.333038332
IMGA|Medtr4g072220.1	Mtr.19928.1.S1_at	1301.618	1517.365	1066.387	419.3217	503.7024	386.0459	1295.123	225.559	436.3567	60.64984	0.003117877	8.0267E-133	0.336922849
IMGA|Medtr3g028450.1	Mtr.19964.1.S1_at	61.05007	109.8719	112.7704	50.82909	27.1107	25.25278	94.5641	29.06016	34.39753	14.26044	0.032301027	2.71682E-13	0.363748239
IMGA|Medtr1g093720.1	Mtr.20055.1.S1_at	23.81601	18.48509	22.81376	8.327995	6.735237	11.59617	21.70495	2.833152	8.886466	2.47812	0.004132566	3.26826E-19	0.409421147
IMGA|Medtr1g075180.1	Mtr.20408.1.S1_s_at	71.45299	91.36798	109.782	33.94361	36.12978	56.91569	90.86766	19.1694	42.32969	12.67905	0.021620091	3.3424E-11	0.465838959
IMGA|Medtr4g038440.1	Mtr.20438.1.S1_at	8258.741	11082.44	10224.74	1774.99	2331.657	1942.266	9855.307	1447.645	2016.305	285.6237	0.000774892	0	0.204590742
IMGA|Medtr3g084240.1	Mtr.20619.1.S1_x_at	926.5662	1019.019	958.7981	236.2988	253.865	212.6983	968.1278	46.92729	234.2874	20.65691	1.57162E-05	0	0.242000449
IMGA|Medtr3g084230.1	Mtr.20620.1.S1_x_at	315.6073	353.1176	397.5816	76.94599	67.7765	62.90656	355.4355	41.03625	69.20968	7.128595	0.000285366	0	0.194717968
IMGA|Medtr3g084200.1	Mtr.20624.1.S1_at	173.6891	212.8687	151.795	51.69831	79.50074	46.2489	179.4509	30.94187	59.14932	17.83422	0.004300824	1.54538E-31	0.329612728
IMGA|Medtr3g084190.1	Mtr.20625.1.S1_x_at	381.6462	406.7256	403.365	86.36498	102.8274	100.1888	397.2456	13.61356	96.4604	8.841877	5.61907E-06	0	0.242823081
IMGA|Medtr3g084150.1	Mtr.20626.1.S1_s_at	265.5777	321.8538	309.3425	62.27606	80.74587	79.23564	298.9246	29.54909	74.08585	10.25542	0.000239297	0	0.247841244
IMGA|Medtr3g084170.1	Mtr.20627.1.S1_x_at	411.531	428.7094	415.8624	107.4086	96.60437	91.02898	418.7009	8.934015	98.34732	8.32775	1.40391E-06	0	0.234886793
IMGA|Medtr1g031530.1	Mtr.20947.1.S1_x_at	19.65755	30.37514	17.95419	13.41394	10.23583	9.852766	22.66229	6.733598	11.16751	1.954868	0.046891037	2.32515E-24	0.492779269
IMGA|Medtr1g025780.1	Mtr.20992.1.S1_at	505.0203	476.9954	521.8867	170.7253	114.0316	141.1983	501.3008	22.67562	141.9851	28.35501	6.7947E-05	0	0.283233295

IMGA|Medtr1g025780.1	Mtr.20993.1.S1_at	109.0087	93.78926	117.1272	36.76636	28.65854	29.6776	106.6417	11.84765	31.70083	4.416369	0.000507677	0	0.297264816
IMGA|Medtr4g098800.1	Mtr.21006.1.S1_at	172.3823	193.5959	110.4656	32.56818	42.30945	21.59912	158.8146	43.19406	32.15891	10.36123	0.007824026	1.7089E-99	0.202493403
IMGA|Medtr3g071890.2	Mtr.21261.1.S1_s_at	103.4407	137.9068	94.8313	57.54218	37.42143	45.365	112.0596	22.7945	46.7762	10.13434	0.010554896	6.57995E-29	0.41742258
IMGA|Medtr2g087840.1	Mtr.21521.1.S1_at	125.0174	214.0036	219.9823	57.75402	105.5802	104.5179	186.3345	53.18619	89.28405	27.31097	0.048242449	7.51263E-10	0.479160182
IMGA|Medtr5g019950.1	Mtr.21616.1.S1_at	131.2322	203.5316	58.89649	54.03165	41.62972	35.41342	131.2201	72.31758	43.69159	9.478825	0.106193081	1.40831E-57	0.332964181
IMGA|Medtr4g127620.1	Mtr.22448.1.S1_s_at	50.95187	83.68131	91.87892	28.14781	44.18607	33.6984	75.50403	21.65425	35.34409	8.144794	0.039682467	1.33868E-17	0.468108653
IMGA|Medtr2g089130.1	Mtr.23279.1.S1_at	38.54565	40.0275	40.8193	14.82385	12.14628	8.344153	39.79748	1.154149	11.77143	3.25607	0.000148838	0	0.295783189
IMGA|Medtr3g099740.1	Mtr.2338.1.S1_at	129.365	155.3442	94.53165	57.11777	62.38348	69.31206	126.4136	30.51351	62.93777	6.116012	0.0241687	2.9877E-72	0.497871788
IMGA|Medtr2g097950.1	Mtr.23402.1.S1_at	76.63864	109.0963	144.1203	37.557	22.07341	50.23723	109.9517	33.74897	36.62255	14.10515	0.025529991	2.16639E-19	0.33307839
IMGA|Medtr4g116360.1	Mtr.24683.1.S1_s_at	81.54089	89.92011	64.31341	21.71384	14.7484	17.88907	78.59147	13.05565	18.1171	3.488312	0.001492774	4.3284E-198	0.230522504
IMGA|Medtr7g074820.1	Mtr.25013.1.S1_at	137.425	149.2531	93.22084	72.35659	50.85687	48.5008	126.633	29.53396	57.23809	13.1459	0.02050931	6.06346E-20	0.451999784
IMGA|Medtr5g032660.1	Mtr.25132.1.S1_at	134.67	113.03	124.8811	54.3285	64.3544	64.83602	124.1937	10.83635	61.17297	5.932375	0.000905711	0	0.492560985
IMGA|Medtr5g024560.1	Mtr.25341.1.S1_at	353.5821	267.0056	289.234	136.6636	73.34723	108.6205	303.2739	44.96346	106.2105	31.72692	0.003436676	5.42313E-27	0.350212957
IMGA|Medtr5g024560.1	Mtr.25341.1.S1_x_at	291.5316	241.9995	238.1184	120.6983	75.3193	107.251	257.2165	29.78107	101.0896	23.30851	0.002023877	0	0.393013459
IMGA|Medtr5g046750.1	Mtr.25344.1.S1_at	163.6983	184.2113	169.3469	85.32722	69.78416	90.9649	172.4188	10.59593	82.02543	10.96961	0.000507712	0	0.475733539
IMGA|Medtr4g037560.1	Mtr.25652.1.S1_at	1534.479	1445.292	2592.338	719.9775	244.4436	511.4398	1857.37	638.0616	491.9536	238.3651	0.025534346	3.35232E-23	0.264865738
IMGA|Medtr4g037480.1	Mtr.25657.1.S1_at	589.953	471.5803	753.4593	237.4288	85.29055	188.7603	604.9975	141.5404	170.4932	77.69671	0.009582318	3.45234E-22	0.281808126
IMGA|Medtr4g072980.1	Mtr.25922.1.S1_at	73.46352	79.40138	81.37048	14.47373	27.22738	17.66167	78.07846	4.116136	19.78759	6.637289	0.00020653	0	0.253432156
IMGA|Medtr4g072820.1	Mtr.25931.1.S1_x_at	318.7938	273.5678	272.9458	62.0849	53.5911	63.21323	288.4358	26.2926	59.62974	5.259963	0.000121988	0	0.20673489
IMGA|Medtr4g072730.1	Mtr.25939.1.S1_at	155.5368	134.234	135.9072	31.7482	32.4469	46.00913	141.8927	11.84574	36.73474	8.03945	0.000219875	0	0.258890999
	Mtr.25941.1.S1_at	38.398	52.00054	64.19831	20.69868	11.77582	16.48928	51.53228	12.90653	16.32126	4.463801	0.011110902	1.69781E-42	0.316719077
IMGA|Medtr3g069690.1	Mtr.2625.1.S1_at	13.5545	20.09972	17.60452	6.986691	5.711567	7.615588	17.08625	3.303246	6.771282	0.970116	0.00656347	9.81806E-76	0.396300112
IMGA|Medtr7g090140.1	Mtr.26276.1.S1_at	38.88221	13.21743	27.22738	10.89288	5.989786	6.981003	26.44234	12.85039	7.954557	2.592478	0.071003103	4.76652E-35	0.300826499
IMGA|Medtr7g113160.1	Mtr.26323.1.S1_at	331.8834	489.6409	501.5383	157.4733	289.5574	175.256	441.0209	94.70285	207.4289	71.67898	0.027114477	1.65646E-08	0.470338048
IMGA|Medtr6g069600.1	Mtr.26465.1.S1_at	476.9413	606.6023	840.0316	112.4037	163.5874	75.32041	641.1918	183.9999	117.1038	44.3208	0.008671985	3.16231E-93	0.182634679
IMGA|Medtr6g069600.1	Mtr.26465.1.S1_s_at	1343.641	1765.247	2255.436	307.1688	456.4552	213.92	1788.108	456.3274	325.848	122.3418	0.005842882	3.32913E-95	0.182230602
	Mtr.26785.1.S1_at	484.1959	443.8997	409.3198	167.9762	241.3482	138.7497	445.8051	37.47438	182.6914	52.85842	0.00215336	0	0.409800941
IMGA|Medtr2g100880.1	Mtr.27132.1.S1_at	53.84878	88.51349	34.85063	13.34335	14.53077	19.94683	59.07097	27.20991	15.94032	3.520169	0.052834074	6.0093E-100	0.269850267
IMGA|Medtr2g100880.1	Mtr.27132.1.S1_s_at	82.86506	204.002	64.89819	18.13365	23.69896	21.51155	117.2551	75.66025	21.11472	2.803796	0.092716538	0	0.180075071
IMGA|Medtr2g027410.1	Mtr.27184.1.S1_at	33.33238	60.00001	61.03979	23.269	26.79814	18.77387	51.45739	15.70533	22.947	4.021817	0.038178782	1.18361E-34	0.445941814
	Mtr.27212.1.S1_at	65.69831	79.94332	86.57025	36.19174	37.29729	36.9152	77.40396	10.66516	36.80141	0.561492	0.002754109	0	0.475446114
IMGA|Medtr3g019390.1	Mtr.27305.1.S1_at	22.51015	22.80882	31.19642	13.82872	9.193088	11.00271	25.50513	4.931062	11.34151	2.336311	0.010856444	8.61046E-26	0.444675502
	Mtr.27494.1.S1_at	205.7216	283.5748	249.2592	112.2483	115.236	140.6824	246.1852	39.01756	122.7222	15.62556	0.007041851	1.23901E-42	0.498495517
	Mtr.27729.1.S1_at	18.25508	20.84196	13.43167	8.663264	7.597883	8.107094	17.50957	3.760978	8.122747	0.532863	0.012845762	2.039E-204	0.463903336
	Mtr.27931.1.S1_at	115.8698	96.16717	107.4002	44.94082	51.86498	36.73869	106.4791	9.883579	44.51483	7.572138	0.000995723	0	0.418061814
IMGA|Medtr6g045140.1	Mtr.28492.1.S1_at	302.9513	351.9356	314.1642	191.4302	130.5133	146.145	323.017	25.66406	156.0295	31.6385	0.002078913	0	0.483037976
	Mtr.28691.1.S1_at	66.33202	59.99491	58.13771	33.86814	27.75002	25.36623	61.48822	4.296412	28.9948	4.385516	0.000786199	0	0.471550459
	Mtr.29286.1.S1_at	83.18267	115.2315	91.69831	31.95891	44.55379	47.91155	96.70415	16.60046	41.47475	8.410233	0.006789223	5.61841E-30	0.428882783
IMGA|Medtr7g105070.1	Mtr.29418.1.S1_at	183.5058	165.5759	80.86498	60.90059	48.58166	47.61541	143.3156	54.82179	52.36589	7.40704	0.04650811	2.2696E-100	0.365388665
IMGA|Medtr1g092420.1	Mtr.3015.1.S1_at	248.4253	203.5206	223.0307	123.1231	92.86847	111.942	224.9922	22.51654	109.3112	15.29795	0.001815034	0	0.485844382
IMGA|Medtr7g111280.1	Mtr.30198.1.S1_at	200.3942	219.3685	196.6122	47.95062	95.27504	59.69501	205.4583	12.19408	67.64022	24.64234	0.000968709	0	0.329216348
IMGA|Medtr7g090310.1	Mtr.30679.1.S1_at	44.0245	51.48199	28.76494	21.58986	21.56142	17.57475	41.42381	11.57967	20.24201	2.309956	0.035973138	8.38586E-57	0.488656431
IMGA|Medtr8g011460.1	Mtr.31281.1.S1_at	179.9465	291.3648	302.3223	75.1462	149.1307	128.5456	257.8779	67.71257	117.6075	38.18583	0.035344457	1.98563E-10	0.456058958
IMGA|Medtr2g098060.1	Mtr.31592.1.S1_at	139.5674	155.3515	125.2092	59.77722	36.83773	17.31544	140.0427	15.07673	37.97679	21.25379	0.002464553	0	0.27118009
IMGA|Medtr2g018990.1	Mtr.31632.1.S1_at	404.6983	426.3861	318.5316	165.9311	164.6983	109.8461	383.2054	57.04917	146.8252	32.03076	0.003326042	2.06243E-37	0.383150114
	Mtr.31658.1.S1_s_at	740.4281	715.7207	753.6686	343.1983	376.8585	292.7031	736.6058	19.26054	337.5867	42.35742	0.000119643	0	0.45830032
IMGA|Medtr8g040520.1	Mtr.31772.1.S1_s_at	169.0573	153.9126	195.2443	61.69225	62.28056	57.30311	172.7381	20.91023	60.42531	2.719857	0.000767228	0	0.349808818
IMGA|Medtr4g084280.1	Mtr.32155.1.S1_at	624.2482	568.2104	540.3518	319.2306	253.4829	239.23	577.6035	42.72963	270.6478	42.67316	0.000918279	0	0.468570289
	Mtr.32301.1.S1_at	280.4098	205.9335	243.9122	111.4827	77.81395	80.62148	243.4185	37.24062	89.97272	18.68104	0.003098216	6.22665E-46	0.369621573
	Mtr.32529.1.S1_at	53.95404	57.61718	53.68868	26.69933	28.96796	21.84452	55.08663	2.195528	25.83727	3.639121	0.000283754	0	0.469029765

IMGA|Medtr7g067330.1	Mtr.32576.1.S1_at	182.0534	182.0376	255.2302	102.3325	106.16	69.68194	206.4404	42.25319	92.72481	20.04727	0.013570113	8.80128E-23	0.449160135
IMGA|Medtr2g089300.1	Mtr.3259.1.S1_at	50.01966	50.69832	30.83503	28.38135	18.10809	14.19792	43.851	11.27727	20.22912	7.325742	0.03831004	2.33697E-08	0.461314759
	Mtr.32691.1.S1_at	509.8228	496.2552	544.5006	209.4677	195.9912	206.6489	516.8596	24.88054	204.0359	7.108072	3.07413E-05	0	0.394760855
IMGA|AC232874_1004.1	Mtr.32965.1.S1_at	278.676	235.9415	154.0325	106.4236	127.8327	73.22222	222.8833	63.33947	102.4928	27.5166	0.039182005	3.5076E-14	0.459849641
	Mtr.33530.1.S1_at	83.47604	84.36498	68.35299	40.12166	34.34208	33.70154	78.73133	8.998896	36.05509	3.536284	0.001572783	5.08002E-97	0.457951015
IMGA|Medtr7g117980.1	Mtr.33765.1.S1_s_at	137.365	151.068	124.1904	66.70628	56.13734	71.48438	137.5411	13.43968	64.776	7.8535	0.001264758	0	0.470957312
IMGA|Medtr5g039180.1	Mtr.33785.1.S1_at	505.7774	385.3381	359.364	169.5085	144.3991	139.6505	416.8265	78.1208	151.186	16.04434	0.004480679	7.3965E-181	0.36270736
	Mtr.33845.1.S1_at	205.4839	182.6095	243.0335	51.57145	54.86498	52.69831	210.3756	30.50757	53.04491	1.6739	0.000873672	0	0.252143817
IMGA|Medtr5g017350.1	Mtr.33852.1.S1_at	47.05817	39.57198	41.91803	21.55067	20.7688	19.60881	42.84939	3.829011	20.64276	0.977047	0.000623957	0	0.481751588
	Mtr.34752.1.S1_s_at	34.70051	28.23361	15.62	7.595207	7.480983	7.127864	26.1847	9.703864	7.401351	0.243636	0.028526409	0	0.282659351
IMGA|Medtr4g097670.1	Mtr.35240.1.S1_s_at	763.5096	704.9913	727.9272	391.8484	389.467	184.8329	732.1427	29.48602	322.0494	118.839	0.004391578	0	0.439872458
IMGA|Medtr1g098430.1	Mtr.35246.1.S1_at	20.23565	26.76013	30.72381	12.58378	11.45783	14.66346	25.90653	5.29593	12.90169	1.626285	0.015274525	1.26434E-43	0.498009202
	Mtr.35334.1.S1_s_at	38.51357	181.1422	103.4758	18.87913	40.9656	19.47184	107.7105	71.40856	26.43886	12.58402	0.124181612	4.76722E-29	0.245462146
	Mtr.35592.1.S1_at	106.7752	146.309	147.6983	63.98047	72.24147	63.69831	133.5942	23.23632	66.64008	4.852996	0.008128992	3.362E-126	0.49882483
	Mtr.35605.1.S1_at	668.0316	729.6983	526.2659	319.9533	311.8025	327.258	641.332	104.3113	319.6713	7.73163	0.005979535	0	0.49844898
	Mtr.35647.1.S1_at	167.6429	154.7598	124.2949	47.63633	33.5705	30.17868	148.8992	22.2603	37.1285	9.256726	0.001305097	0	0.249353249
	Mtr.35957.1.S1_at	183.0557	169.8105	196.7183	92.39281	86.14586	75.39477	183.1948	13.45447	84.64448	8.5979	0.000433766	0	0.462046215
	Mtr.36075.1.S1_at	333.5825	369.4746	383.4558	124.9045	194.6044	151.0378	362.171	25.72632	156.8489	35.21143	0.0012306	0	0.43307965
	Mtr.36075.1.S1_x_at	345.7443	383.8477	384.2745	136.4661	202.109	165.6789	371.2889	22.12324	168.0847	32.8875	0.000888565	0	0.452705908
	Mtr.36078.1.S1_at	202.3014	242.121	241.6301	115.4892	126.7409	99.85295	228.6842	22.84946	114.0277	13.50342	0.001706001	0	0.498625179
	Mtr.36333.1.S1_at	1473.469	1394.707	909.6767	622.4203	822.0316	415.7288	1259.284	305.3196	620.0602	203.1617	0.039202262	5.04571E-08	0.492390925
	Mtr.36426.1.S1_at	189.8434	193.9399	185.6053	89.96297	95.45644	99.26118	189.7962	4.167508	94.89353	4.674594	1.25204E-05	0	0.499975922
	Mtr.36871.1.S1_at	98.33874	88.32027	66.54517	40.25432	43.50026	30.96089	84.40139	16.25503	38.23849	6.508195	0.010288317	1.08374E-34	0.453055218
	Mtr.37181.1.S1_at	1402.553	938.6412	839.4191	507.5349	384.7875	538.6947	1060.204	300.6045	477.0057	81.36879	0.031566238	2.18827E-35	0.449918688
	Mtr.37188.1.S1_at	534.865	579.3736	645.1983	205.4935	168.0513	221.864	586.479	55.50879	198.4696	27.58539	0.000410637	0	0.338408727
	Mtr.37355.1.S1_at	3282.34	3307.841	3339.698	1678.763	1770.032	1485.41	3309.96	28.73776	1644.735	145.3299	4.10349E-05	0	0.49690485
	Mtr.37375.1.S1_at	421.1188	1366.084	721.1994	142.0316	335.1481	209.532	836.134	482.8532	228.9039	98.00479	0.099682174	7.22917E-27	0.273764606
IMGA|Medtr8g087710.1	Mtr.37525.1.S1_at	153.3234	108.2718	153.5963	79.50937	59.09506	49.49226	138.3972	26.08969	62.6989	15.32962	0.012321701	1.20003E-17	0.453036054
	Mtr.37590.1.S1_s_at	5164.547	3982.801	4548.112	1700.198	2229.721	1335.434	4565.153	591.0574	1755.118	449.6657	0.002803199	2.65327E-27	0.384459766
IMGA|Medtr2g035780.1	Mtr.37806.1.S1_at	828.6852	711.5232	639.0316	293.7039	249.5077	333.6889	726.4133	95.69954	292.3002	42.10812	0.001980842	2.57016E-71	0.402388215
	Mtr.37919.1.S1_at	477.0225	385.177	437.1647	185.8198	235.3128	207.763	433.1214	46.05606	209.6319	24.79938	0.001778541	0	0.484002636
IMGA|Medtr4g115920.2	Mtr.38047.1.S1_at	201.139	662.0615	394.6585	113.9564	164.7778	117.1983	419.2863	231.4461	131.9775	28.45208	0.099753595	1.70076E-68	0.314767015
	Mtr.38088.1.S1_at	157.0895	101.4916	199.7933	71.36498	44.71355	75.03165	152.7914	49.2916	63.70339	16.54756	0.041238698	1.11045E-20	0.416930375
	Mtr.38270.1.S1_at	40.78013	43.15285	66.53737	15.34752	18.70559	24.91468	50.15678	14.23552	19.65593	4.853863	0.024616178	1.37552E-27	0.391889826
	Mtr.38407.1.S1_at	1204.365	1305.349	1639.408	467.9257	436.8325	379.4801	1383.041	227.6899	428.0794	44.86781	0.002048715	1.6957E-297	0.30952045
	Mtr.38426.1.S1_at	182.882	304.0341	328.9643	77.36498	130.1075	152.5316	271.9601	78.14448	120.0014	38.58895	0.039164181	9.06523E-12	0.441246152
IMGA|Medtr6g090590.1	Mtr.38431.1.S1_at	135.6618	230.4651	245.9643	67.19883	108.2892	88.12041	204.0304	59.71395	87.86948	20.54634	0.033342836	1.21462E-22	0.430668584
	Mtr.38471.1.S1_at	1233.406	996.6193	702.5045	434.8453	421.4866	345.6624	977.51	265.9663	400.6647	48.09947	0.020897137	7.74572E-96	0.409883001
IMGA|Medtr1g087880.1	Mtr.38511.1.S1_at	427.839	304.2073	320.3975	161.5929	154.8341	171.582	350.8146	67.19452	162.6697	8.425755	0.0085724	0	0.46369126
	Mtr.38539.1.S1_at	92.99409	142.797	138.7109	47.86499	40.30999	68.86498	124.834	27.64974	52.34665	14.79564	0.016081535	2.14376E-17	0.419330148
IMGA|Medtr5g065010.1	Mtr.38650.1.S1_at	685.4471	471.383	466.5027	126.2028	125.3727	121.7345	541.1109	125.0226	124.4367	2.376656	0.004474165	0	0.229965236
IMGA|Medtr8g070540.1	Mtr.38972.1.S1_at	740.4687	960.9483	1120.032	234.8635	509.6427	348.0267	940.4829	190.6073	364.1776	138.0997	0.013255074	4.90029E-13	0.387224112
IMGA|Medtr4g010340.1	Mtr.39011.1.S1_at	528.4194	557.0316	108.0629	72.96461	125.7491	105.0457	397.838	251.36	101.2531	26.59584	0.111914649	4.0132E-83	0.254508497
	Mtr.39027.1.S1_at	26.52753	37.95617	26.2383	10.74003	18.10946	10.32911	30.24067	6.683391	13.05953	4.378188	0.020392703	1.06819E-11	0.431853241
IMGA|Medtr7g065510.1	Mtr.39126.1.S1_at	11.6676	10.60022	11.23601	5.325648	4.381649	4.71533	11.16794	0.536938	4.807542	0.478707	0.000106044	0	0.430476938
	Mtr.39376.1.S1_at	58.65404	67.07598	40.36524	27.43978	18.78704	30.33431	55.36509	13.65572	25.52038	6.008156	0.025703206	7.71713E-18	0.460947143
IMGA|Medtr7g101290.1	Mtr.39530.1.S1_s_at	392.8565	306.9765	362.8676	177.2569	165.0239	181.7161	354.2335	43.58617	174.6656	8.642539	0.002192725	1.3274E-283	0.493080411
IMGA|Medtr3g098980.1	Mtr.39535.1.S1_at	53.24757	49.34538	52.22818	24.03677	24.62714	26.70044	51.60704	2.023887	25.12145	1.398943	4.87026E-05	0	0.486783322
	Mtr.39804.1.S1_at	29.35663	58.36498	59.21013	16.61771	21.84336	20.80038	48.97725	16.99721	19.75382	2.765557	0.042419707	7.9225E-75	0.403326399
IMGA|AC225518_18.2	Mtr.40137.1.S1_s_at	1071.94	1127.859	1361.512	258.3521	234.1164	196.9108	1187.104	153.6082	229.7931	30.94799	0.000451327	0	0.19357456

	Mtr.40143.1.S1_at	20.10334	28.09201	17.94461	7.819725	12.33316	9.126064	22.04666	5.345536	9.759651	2.322467	0.021743244	5.03093E-20	0.442681698
	Mtr.40190.1.S1_at	220.1856	251.0555	181.8763	113.3249	104.1811	73.09395	217.7058	34.65621	96.86662	21.08928	0.006701935	3.25872E-23	0.444942749
IMGA|Medtr5g016320.1	Mtr.40263.1.S1_at	90.53596	104.1354	105.6983	36.53094	26.38358	48.20882	100.1232	8.33951	37.04112	10.92156	0.001355046	0	0.369955268
IMGA|Medtr4g132020.1	Mtr.40306.1.S1_at	1024.115	942.1604	949.5626	502.6202	377.3338	280.0316	971.946	45.33101	386.6619	111.5871	0.001090859	0	0.397822388
IMGA|Medtr3g064420.1	Mtr.40695.1.S1_at	1363.911	1105.35	1333.625	855.2842	411.0033	418.3687	1267.629	141.3507	561.5521	254.4061	0.0136729	0	0.442994055
IMGA|Medtr3g064420.1	Mtr.40695.1.S1_s_at	772.0437	638.9089	838.8918	513.3473	254.9143	223.7528	749.9481	101.8059	330.6715	158.9673	0.01834945	9.80105E-13	0.440925768
IMGA|Medtr7g072560.1	Mtr.40715.1.S1_at	565.7893	611.9814	506.7536	288.3325	215.453	261.0861	561.5081	52.74436	254.9572	36.82429	0.001175258	0	0.454057923
	Mtr.40946.1.S1_at	449.2439	359.0255	789.7825	18.46249	55.44076	5.38403	532.6839	227.1774	26.42909	25.96188	0.01854118	4.6135E-250	0.049614958
IMGA|Medtr8g070540.1	Mtr.40990.1.S1_s_at	145.9144	150.9126	218.6821	53.52289	80.58472	70.50573	171.8364	40.64648	68.20444	13.6769	0.013857436	2.39897E-39	0.396915068
IMGA|Medtr5g090630.1	Mtr.41054.1.S1_at	147.1979	112.472	120.7806	59.11265	86.87877	32.04941	126.8168	18.13279	59.34694	27.41543	0.02368543	1.15792E-10	0.467973719
	Mtr.41121.1.S1_at	1034.781	1771.956	1883.752	397.0967	969.8388	682.4482	1563.496	461.2802	683.1279	286.3716	0.048392158	1.0112E-07	0.436923258
	Mtr.41140.1.S1_at	580.5566	508.4682	444.892	156.8948	183.8221	133.7971	511.3056	67.8768	158.1713	25.03689	0.001072445	0	0.30934792
	Mtr.41157.1.S1_at	138.6766	141.1228	99.41755	63.26436	46.72139	65.7674	126.4057	23.40438	58.58438	10.3496	0.010104132	7.40047E-30	0.463463263
	Mtr.41215.1.S1_at	771.8585	896.0316	567.7617	217.2363	222.865	240.5447	745.2173	165.7486	226.882	12.16234	0.005684596	0	0.304450837
	Mtr.4126.1.S1_at	88.10896	130.2327	105.018	43.80151	29.2859	49.32274	107.7865	21.19787	40.80338	10.34941	0.007939431	3.63672E-29	0.378557298
	Mtr.41286.1.S1_at	128.5404	173.9629	875.5267	109.1008	57.98755	221.2285	392.6767	418.7766	129.439	83.49932	0.345798849	4.75018E-08	0.329632404
IMGA|Medtr4g016510.1	Mtr.41305.1.S1_at	363.5316	381.4741	339.5285	125.8323	129.7473	140.5307	361.5114	21.04565	132.0368	7.612004	5.90584E-05	0	0.365235397
	Mtr.41407.1.S1_at	665.4701	519.2569	375.4425	288.0426	143.6525	183.8666	520.0565	145.0154	205.1872	74.51879	0.028702622	2.50662E-13	0.394547902
IMGA|Medtr2g077690.1	Mtr.41715.1.S1_at	159.0515	178.2471	153.9596	51.15088	61.74875	75.79329	163.7527	12.80811	62.89764	12.36131	0.000604427	0	0.384101276
	Mtr.41864.1.S1_at	56.75079	56.95325	35.3296	29.06663	18.69837	20.39445	49.67788	12.42638	22.71982	5.561538	0.026542581	4.63356E-17	0.457342784
	Mtr.41926.1.S1_at	203.641	309.8092	270.2333	57.2051	66.26426	64.66713	261.2278	53.65395	62.71216	4.835652	0.003091967	0	0.240066936
	Mtr.42164.1.S1_at	1159.839	1428.698	1245.371	521.905	896.0789	397.3983	1277.969	137.3623	605.1274	259.5478	0.016558268	0	0.473507056
IMGA|Medtr3g089570.1	Mtr.42189.1.S1_at	345.7912	411.793	476.4747	133.0316	145.5509	113.5464	411.3529	65.34289	130.7096	16.12813	0.001949335	1.4872E-199	0.317755428
IMGA|Medtr2g100880.1	Mtr.42339.1.S1_at	482.9786	961.3059	267.865	79.19831	115.1113	89.28773	570.7165	354.9486	94.53245	18.52204	0.081093195	0	0.165638196
IMGA|Medtr8g091320.3	Mtr.42849.1.S1_at	446.8542	749.8255	623.7483	38.22419	106.3038	45.69214	606.8093	152.1943	63.40671	37.33716	0.003868078	3.2602E-140	0.104491987
IMGA|Medtr7g093140.1	Mtr.43089.1.S1_at	142.3043	174.0548	142.4171	31.30232	40.33281	40.27768	152.9254	18.2987	37.30427	5.197911	0.000460426	0	0.243937644
IMGA|Medtr4g015120.1	Mtr.43288.1.S1_at	274.3967	255.4158	179.7499	111.4328	65.6524	135.5675	236.5208	50.07264	104.2176	35.51164	0.0202443	1.09673E-10	0.440627603
	Mtr.43345.1.S1_at	3755.159	3875.948	5089.87	2179.95	1707.449	1832.532	4240.326	738.2014	1906.643	244.8136	0.006528673	3.07027E-61	0.449645498
IMGA|AC225518_3.1	Mtr.4357.1.S1_at	254.2623	246.5129	317.4671	69.39631	60.90278	71.60543	272.7474	38.9217	67.30151	5.650475	0.000826868	0	0.246753964
	Mtr.43771.1.S1_at	444.5056	405.6382	226.5399	129.1104	140.6333	136.4282	358.8945	116.2583	135.3907	5.831118	0.029224046	0	0.377243555
IMGA|Medtr2g062280.1	Mtr.43826.1.S1_at	63.1481	68.30734	68.21062	18.70559	22.6694	29.65997	66.55535	2.951161	23.67832	5.546443	0.000293194	0	0.355768844
	Mtr.44100.1.S1_at	226.021	182.2285	109.0774	79.19831	88.01327	50.47974	172.4423	59.08284	72.56377	19.62662	0.049884816	1.20449E-18	0.420800335
IMGA|Medtr2g086790.1	Mtr.44119.1.S1_at	418.9933	416.5831	453.0316	205.1983	174.0316	236.9905	429.536	20.38347	205.4068	31.47994	0.000491618	0	0.478206278
IMGA|Medtr7g058750.1	Mtr.44423.1.S1_at	247.8399	213.4663	164.3096	106.6503	99.84505	100.1025	208.5386	41.98262	102.1993	3.856822	0.011979528	0	0.490073753
	Mtr.44458.1.S1_at	74.71354	55.71145	74.5399	13.56139	17.77094	11.61491	68.32163	10.92108	14.31574	3.146578	0.001188221	0	0.209534586
	Mtr.44509.1.S1_at	2467.787	3155.83	3365.179	1003.683	1925.895	1504.877	2996.265	469.493	1478.152	461.6864	0.016220367	1.23144E-08	0.493331409
IMGA|CU633465_8.1	Mtr.44525.1.S1_at	96.46003	96.31746	62.29465	44.36502	40.37303	38.21983	85.02405	19.68436	40.98596	3.11811	0.018662077	3.7287E-132	0.482051401
IMGA|Medtr6g005480.1	Mtr.44648.1.S1_at	109.116	94.44713	59.69738	45.77344	43.11605	37.65614	87.75352	25.38019	42.18188	4.138493	0.037312554	4.25711E-81	0.480685857
IMGA|Medtr4g064530.1	Mtr.44766.1.S1_at	55.4651	56.73147	66.05993	30.87395	21.4151	23.33277	59.41883	5.786104	25.20727	5.000276	0.001494553	0	0.424230367
	Mtr.44950.1.S1_at	108.7572	101.0082	144.2615	46.88203	31.64282	47.40487	118.009	23.06315	41.97657	8.953112	0.005993273	5.63783E-49	0.355706603
	Mtr.45240.1.S1_at	54.43334	68.86199	54.72307	22.4422	24.55341	14.48819	59.33947	8.248016	20.4946	5.307734	0.002364753	8.02579E-37	0.345378961
IMGA|Medtr4g082230.1	Mtr.45436.1.S1_at	153.9234	128.6591	98.27061	68.87963	53.13444	63.84237	126.951	27.86566	61.95215	8.040986	0.017814582	1.53833E-44	0.488000312
IMGA|Medtr3g031040.1	Mtr.45460.1.S1_at	504.7513	868.3772	795.2918	252.8066	410.7933	409.5196	722.8067	192.3448	357.7065	90.84822	0.04103081	3.38454E-12	0.49488539
IMGA|Medtr2g099780.1	Mtr.45681.1.S1_s_at	75.52508	115.996	129.0732	35.3367	57.87494	64.03641	106.8648	27.91747	52.41602	15.10854	0.041106249	4.31932E-10	0.490489293
IMGA|Medtr3g095180.1	Mtr.45802.1.S1_at	454.2753	430.7566	484.4822	141.1674	144.365	164.2338	456.5047	26.93209	149.922	12.49702	5.74352E-05	0	0.328412913
IMGA|Medtr8g045300.1	Mtr.45935.1.S1_at	2364.89	2640.665	3252.382	1047.494	1213.694	1150.023	2752.645	454.2196	1137.07	83.85368	0.003747434	3.6465E-244	0.413082774
IMGA|Medtr8g087860.1	Mtr.46049.1.S1_at	17.69933	17.07397	28.72219	5.577539	6.961278	8.918741	21.16517	6.552043	7.152519	1.678791	0.02299532	2.26328E-47	0.337938242
IMGA|Medtr4g114570.1	Mtr.46451.1.S1_at	64.53165	69.68992	123.7754	33.36835	24.78207	33.095	85.999	32.81686	30.41514	4.880296	0.044039924	1.26278E-86	0.353668531
IMGA|Medtr7g073380.1	Mtr.47266.1.S1_at	697.124	779.0349	682.5316	370.1012	205.67	472.865	719.5635	52.01797	349.5454	134.7783	0.0113673	0	0.485774166
IMGA|Medtr4g009170.1	Mtr.47758.1.S1_at	148.1793	124.4739	156.1983	72.9685	44.81979	81.19831	142.9505	16.4959	66.32887	19.0765	0.006244799	8.88178E-16	0.463998801

IMGA|Medtr7g080550.1	Mtr.48575.1.S1_at	912.0136	1506.314	1554.857	366.0316	834.9435	586.5092	1324.395	357.9566	595.8281	234.5948	0.042030719	7.48482E-08	0.449886991
IMGA|Medtr7g101080.1	Mtr.48994.1.S1_at	2017.48	1993.803	1956.374	710.8311	484.1412	708.7148	1989.219	30.80958	634.5623	130.2728	6.22176E-05	0	0.319000748
IMGA|Medtr3g110550.1	Mtr.49056.1.S1_at	49.5454	81.64142	93.19777	22.55227	25.95039	33.89977	74.79486	22.61723	27.46748	5.823885	0.024674437	5.382E-45	0.367237458
IMGA|Medtr5g029770.1	Mtr.49103.1.S1_s_at	748.6473	1243.801	1306.199	314.4912	720.1937	467.5711	1099.549	305.487	500.752	204.8764	0.047844692	4.14223E-07	0.455415882
IMGA|Medtr3g099580.1	Mtr.49274.1.S1_at	504.4296	449.6957	251.9823	153.5474	90.18547	117.836	402.0359	132.8006	120.523	31.76633	0.023357121	3.57375E-53	0.299781663
IMGA|Medtr4g072240.1	Mtr.49768.1.S1_x_at	671.2477	756.9342	476.7737	193.7849	211.8505	171.9427	634.9852	143.5573	192.526	19.98368	0.006139346	0	0.303197671
IMGA|Medtr4g072490.1	Mtr.49785.1.S1_x_at	19.97232	13.4798	14.31565	7.637352	5.787001	6.375997	15.92259	3.53198	6.600117	0.945316	0.011544737	2.07522E-65	0.414512713
IMGA|Medtr4g072580.1	Mtr.49791.1.S1_at	90.03418	85.68029	91.69831	22.86668	7.562889	7.570704	89.13759	3.107581	12.66676	8.833394	0.000145028	0	0.14210344
IMGA|Medtr4g072580.1	Mtr.49791.1.S1_x_at	79.21261	58.08539	82.72051	30.15815	5.56877	9.894154	73.3395	13.32637	15.20702	13.12743	0.005758533	1.71892E-14	0.207351074
IMGA|Medtr4g072610.1	Mtr.49793.1.S1_x_at	95.69446	89.83411	100.3937	20.10334	24.774	24.10099	95.30743	5.290443	22.99278	2.524846	2.83718E-05	0	0.241248505
IMGA|Medtr4g072660.1	Mtr.49795.1.S1_at	767.0219	621.8174	583.2235	149.1984	148.5132	181.3719	657.3543	96.91548	159.6945	18.77632	0.000947775	0	0.242935265
IMGA|Medtr4g130510.1	Mtr.50224.1.S1_s_at	410.0474	324.7122	353.7747	147.1857	107.6983	165.5141	362.8447	43.38459	140.1327	29.54615	0.001825768	0	0.386205694
IMGA|Medtr4g079500.1	Mtr.50393.1.S1_s_at	159.6983	149.4918	195.9082	83.53165	83.8977	82.67903	168.3661	24.39201	83.36946	0.625313	0.003803979	0	0.495167696
IMGA|Medtr8g065810.1	Mtr.50411.1.S1_at	34.45753	37.06668	18.86448	14.11912	13.51288	14.92674	30.12956	9.842691	14.18625	0.709314	0.04889417	0	0.470841413
IMGA|Medtr8g013680.1	Mtr.50501.1.S1_at	3055.465	2603.02	2482.866	1186.949	1393.731	1268.151	2713.784	301.9419	1282.944	104.1817	0.001487005	4.4319E-125	0.472750824
IMGA|Medtr7g011060.2	Mtr.50574.1.S1_at	799.6627	841.4206	868.854	223.672	246.7622	201.6543	836.6458	34.84191	224.0295	22.55607	1.39051E-05	0	0.267771005
IMGA|Medtr4g115460.1	Mtr.50722.1.S1_at	28.38516	38.69873	43.55266	20.95976	14.04246	19.67229	36.87885	7.74579	18.22483	3.678805	0.019640294	1.5968E-18	0.494181164
IMGA|Medtr4g064530.1	Mtr.50799.1.S1_s_at	97.90086	82.34003	130.73	44.29302	17.26634	49.32104	103.657	24.70317	36.96013	17.23962	0.018540184	2.0703E-11	0.356562009
IMGA|Medtr4g116190.1	Mtr.50874.1.S1_at	167.1423	136.952	104.7587	61.07744	64.7989	60.6167	136.2843	31.19716	62.16435	2.29319	0.014804173	0	0.45613726
IMGA|Medtr4g059720.1	Mtr.50904.1.S1_at	502.7366	366.4527	481.1535	133.9698	104.7287	173.7234	450.1142	73.2523	137.474	34.63059	0.002606322	4.09284E-55	0.305420186
IMGA|Medtr2g010250.1	Mtr.51059.1.S1_x_at	396.4541	397.2662	328.5622	162.2744	233.842	159.5598	374.0942	39.43395	185.2254	42.12507	0.00477453	0	0.495130398
IMGA|Medtr2g035780.1	Mtr.51462.1.S1_s_at	662.7546	471.0883	443.0399	231.0037	164.0486	224.7701	525.6276	119.5807	206.6074	36.98862	0.011560621	1.84746E-50	0.39306812
IMGA|Medtr2g081590.1	Mtr.51510.1.S1_at	943.9709	1296.662	2392.313	50.52779	48.07612	50.91371	1544.315	755.2634	49.83921	1.539023	0.026601753	0	0.032272686
IMGA|Medtr6g009320.1	Mtr.51616.1.S1_at	67.97149	104.2598	118.4958	31.17606	43.36505	47.58483	96.90902	26.05189	40.70864	8.520814	0.023770082	3.17245E-30	0.420070714
IMGA|Medtr3g077410.1	Mtr.51752.1.S1_at	198.8477	150.0852	105.4869	48.90694	55.72134	82.84282	151.4732	46.69589	62.49036	17.95204	0.036904885	9.06353E-18	0.412550522
IMGA|Medtr3g088970.1	Mtr.52006.1.S1_at	883.8336	731.6428	957.5671	179.903	411.2785	238.1347	857.6811	115.2103	276.4388	120.3497	0.003782964	0	0.322309472
IMGA|Medtr3g088970.1	Mtr.52006.1.S1_s_at	1265.878	1080.617	1415.472	249.3037	624.8296	362.5422	1253.989	167.7439	412.2251	192.6298	0.00465811	0	0.328731125
IMGA|Medtr4g014070.1	Mtr.5210.1.S1_at	108.4841	160.5994	169.5316	50.09827	91.46639	77.41178	146.205	32.97119	72.99215	21.03522	0.031603101	1.65604E-09	0.499245121
IMGA|Medtr4g072600.1	Mtr.5470.1.S1_at	70.94149	52.39558	45.68347	15.06448	12.58063	16.32209	56.34018	13.08288	14.65574	1.903925	0.005466257	0	0.260129383
IMGA|Medtr4g072600.1	Mtr.5470.1.S1_x_at	74.14692	74.46261	80.62	17.22189	17.69482	18.43582	76.40985	3.649518	17.78418	0.611879	1.04892E-05	0	0.232747173
IMGA|Medtr1g099440.1	Mtr.5500.1.S1_at	74.17072	76.00669	75.3523	29.8327	19.22557	25.40801	75.17657	0.930513	24.8221	5.327784	8.64947E-05	0	0.330183929
IMGA|Medtr4g072680.1	Mtr.5540.1.S1_at	540.1569	433.3388	427.4917	70.58743	83.64585	79.42744	466.9958	63.4268	77.88691	6.664122	0.00045369	0	0.16678287
IMGA|Medtr5g070330.1	Mtr.5594.1.S1_at	246.7451	330.3635	71.77926	60.523	70.80336	33.40525	216.296	131.9538	54.91053	19.32044	0.104119343	1.93058E-47	0.253867603
	Mtr.5719.1.S1_at	295.7656	252.8464	446.7732	92.78288	78.26999	92.53165	331.7951	101.8602	87.8615	8.307448	0.014444822	0	0.264806511
IMGA|Medtr6g012520.1	Mtr.6141.1.S1_s_at	350.4945	329.5653	475.4329	97.26805	95.5197	63.48824	385.1642	78.87227	85.42533	19.01818	0.003062841	4.4496E-164	0.221789372
	Mtr.6179.1.S1_at	78.11472	84.88977	98.65733	34.98696	43.41517	50.20979	87.2206	10.46778	42.87064	7.626013	0.004049821	7.2772E-24	0.491519643
	Mtr.6371.1.S1_s_at	1005.147	941.2754	1565.658	571.3208	542.3145	528.5399	1170.693	343.537	547.3917	21.83769	0.034974113	0	0.467579086
IMGA|Medtr1g110390.1	Mtr.6507.1.S1_at	56.6705	54.35721	39.38619	17.4271	12.96119	13.23916	50.13797	9.382875	14.54248	2.502018	0.003152971	4.5666E-134	0.2900493
IMGA|Medtr5g010150.1	Mtr.6632.1.S1_at	1595.315	1941.987	1738.683	656.9583	997.0287	795.2666	1758.662	174.1975	816.4178	171.019	0.002603046	0	0.46422677
	Mtr.6694.1.S1_at	150.1754	191.0387	82.33801	49.01993	63.068	28.36176	141.184	54.90532	46.81657	17.45772	0.047011489	7.78135E-21	0.331599544
	Mtr.6710.1.S1_at	27.73587	40.34551	26.2383	13.20086	21.34299	11.38983	31.43989	7.748756	15.31122	5.301564	0.040923892	1.36929E-07	0.486999802
IMGA|Medtr4g072890.1	Mtr.697.1.S1_at	1050.995	980.904	817.527	346.7758	345.5226	393.3232	949.8088	119.8	361.8739	27.24315	0.001156654	0	0.380996573
IMGA|Medtr4g072890.1	Mtr.697.1.S1_s_at	167.4051	205.964	157.1498	58.77144	57.97373	59.86009	176.8396	25.73836	58.86842	0.946916	0.001366618	0	0.332891598
IMGA|Medtr4g072860.1	Mtr.699.1.S1_at	508.4879	498.4589	423.8387	158.962	168.6171	162.6983	476.9285	46.24975	163.4258	4.868492	0.000307616	0	0.342663038
IMGA|Medtr4g072850.1	Mtr.701.1.S1_x_at	379.6703	434.865	424.6513	133.6291	176.6745	183.815	413.0622	29.36568	164.7062	27.14935	0.000423565	0	0.398744232
IMGA|Medtr4g072830.1	Mtr.702.1.S1_at	147.7947	151.8748	185.7103	25.3234	20.65025	30.37514	161.7933	20.81301	25.4496	4.863672	0.000381538	0	0.157297026
	Mtr.703.1.S1_at	41.5128	43.8058	51.69831	9.508369	6.035124	9.010658	45.6723	5.343132	8.184717	1.878162	0.000330385	0	0.179205259
IMGA|Medtr4g072720.1	Mtr.705.1.S1_x_at	241.2415	184.2006	144.0015	38.43209	42.19681	70.66593	189.8145	48.86249	50.43161	17.62425	0.009678198	1.0425E-42	0.265688881
IMGA|Medtr4g107840.1	Mtr.7573.1.S1_at	115.7413	135.2914	114.1391	46.33737	39.54899	39.80659	121.724	11.77704	41.89765	3.847066	0.000366975	0	0.344202143
IMGA|Medtr7g105070.1	Mtr.762.1.S1_at	269.1189	199.6245	114.8808	84.37892	49.59725	65.11372	194.5414	77.24459	66.3633	17.42447	0.048628693	3.484E-37	0.341126867

	Mtr.8618.1.S1_at	1390.222	1729.581	2128.948	640.9047	1036.148	724.4009	1749.584	369.7692	800.4845	208.3166	0.017942534	2.99064E-15	0.457528517
IMGA|Medtr4g097670.1	Mtr.8650.1.S1_at	3003.843	3108.241	2781.204	1591.599	2150.904	682.5316	2964.43	167.0429	1475.012	741.0965	0.027383269	0	0.497570194
	Mtr.8680.1.S1_at	4915.207	4048.07	4242.868	1616.978	2232.791	1314.087	4402.048	454.9564	1721.285	468.1498	0.002064583	0	0.391019148
	Mtr.8859.1.S1_at	1685.809	1738.144	1243.306	606.1926	737.3251	560.6965	1555.753	271.8496	634.738	91.70904	0.005122522	9.06093E-68	0.407994082
	Mtr.8959.1.S1_at	316.309	347.499	542.4158	100.2213	84.32751	76.12275	402.0746	122.5354	86.89052	12.25201	0.011395027	0	0.216105452
	Mtr.9169.1.S1_at	207.1983	212.8385	67.87895	48.84117	50.80593	50.34881	162.6386	82.11271	49.99864	1.028121	0.076337857	0	0.307421711
	Mtr.9315.1.S1_at	101.9127	90.54499	111.7053	55.77458	40.74052	49.30008	101.3877	10.5899	48.60506	7.541088	0.00215476	0	0.47939814
	Mtr.9328.1.S1_at	125.4456	106.9691	154.6464	73.33568	34.5339	62.77285	129.0203	24.03883	56.88081	20.0607	0.016254694	4.70737E-10	0.440866993
	Mtr.9594.1.S1_at	380.6852	336.2333	426.7921	213.349	141.9931	165.9695	381.2369	45.28194	173.7705	36.31194	0.003460356	4.33345E-23	0.455807225

Supplementary Table S2. Total up- or down-regulated genes analysed by MapMan. Elements (genes) in different subgroups but under the same bin numbers are grouped together. 312 elements from 20 groups are down-regulated and 603 elements from 22 groups are up-regulated.

Down-regulated genes

bin	name	elements	p-value
35	not assigned	139	1.93E-08
35	hormone metabolism	45	0.194
11	lipid metabolism	9	0.409
28	DNA	5	0.488
15	metal handling	2	0.488
21	redox.regulation	2	0.488
27	RNA	7	0.488
31	cell	6	0.488
20	stress	5	0.488
16	secondary metabolism	17	0.488
29	protein	2	0.622
34	transport	15	0.638
26	misc	18	0.721
33	development	4	0.721
30	signalling	12	0.721
10	cell wall	15	0.721
23	nucleotide metabolism	3	0.721
5	fermentation	1	0.782
3	minor CHO metabolism	2	0.911
13	amino acid metabolism	3	0.951
		312	
17.2	hormone metabolism.auxin	41	1.29E-08
17.2.3	hormone metabolism.auxin.induced-regulated-responsive-activated	41	1.29E-08
11.3	lipid metabolism.Phospholipid synthesis	4	0.194
35.2	not assigned.unknown	139	0.194
30.10	signalling.phosphorelay	3	0.303
30.2	signalling.receptor kinases	3	0.409
27.3.35	RNA.regulation of transcription.bZIP transcription factor family	2	0.488
34.19	transport.Major Intrinsic Proteins	5	0.488
20.2.3	stress.abiotic.drought/salt	1	0.488
30.2.3	signalling.receptor kinases.leucine rich repeat III	1	0.488
26.28	misc.GDSL-motif lipase	4	0.488
26.10	misc.cytochrome P450	3	0.488
3.4	minor CHO metabolism.myo-inositol	1	0.488
3.4.3	minor CHO metabolism.myo-inositol.InsP Synthases	1	0.488
26.2	misc.UDP glucosyl and glucoronyl transferases	4	0.488
34.10	transport.nucleotides	1	0.488
16.10	secondary metabolism.simple phenols	6	0.488
34.16	transport.ABC transporters and multidrug resistance systems	1	0.488
21.2	redox.ascorbate and glutathione	2	0.488
13.2	amino acid metabolism.degradation	1	0.488
13.2.3	amino acid metabolism.degradation.aspartate family	1	0.488
13.2.3.5	amino acid metabolism.degradation.aspartate family.lysine	1	0.488

20.2	stress.abiotic	3	0.488
30.2.17	signalling.receptor kinases.DUF 26	2	0.488
15.1	metal handling.acquisition	1	0.488
16.4	secondary metabolism.N misc	1	0.488
16.4.1	secondary metabolism.N misc.alkaloid-like	1	0.488
34.13	transport.peptides and oligopeptides	1	0.488
16.2.1	secondary metabolism.phenylpropanoids.lignin biosynthesis	1	0.488
16.2.1.9	secondary metabolism.phenylpropanoids.lignin biosynthesis.COMT	1	0.488
27.3	RNA.regulation of transcription	7	0.488
34.19.2	transport.Major Intrinsic Proteins.TIP	2	0.488
28.1.3	DNA.synthesis/chromatin structure.histone	2	0.488
34.9	transport.metabolite transporters at the mitochondrial membrane	1	0.488
3.5	minor CHO metabolism.others	1	0.488
16.8.2	secondary metabolism.flavonoids.chalcones	1	0.488
17.1	hormone metabolism.abscisic acid	1	0.488
17.1.1	hormone metabolism.abscisic acid.synthesis-degradation	1	0.488
17.1.1.1	hormone metabolism.abscisic acid.synthesis-degradation.synthesis	1	0.488
17.1.1.1.1	hormone.ABA.synthesis-degradation.synthesis.zeaxanthin epoxidase	1	0.488
34.8	transport.metabolite transporters at the envelope membrane	1	0.488
31.1	cell.organisation	6	0.488
34.19.1	transport.Major Intrinsic Proteins.PIP	3	0.488
16.8	secondary metabolism.flavonoids	5	0.488
28.1	DNA.synthesis/chromatin structure	4	0.488
29.4	protein.postranslational modification	1	0.488
11.9	lipid metabolism.lipid degradation	1	0.488
11.9.3	lipid metabolism.lipid degradation.lysophospholipases	1	0.488
11.9.3.3	lipid.lipid degradation.lysophospholipases.glycerophosphodiesterase	1	0.488
17.5	hormone metabolism.ethylene	3	0.488
17.5.1	hormone metabolism.ethylene.synthesis-degradation	3	0.488
34.21	transport.calcium	1	0.522
28.2	DNA.repair	1	0.523
27.3.11	RNA.regulation of transcription.C2H2 zinc finger family	1	0.543
23.2	nucleotide metabolism.degradation	1	0.572
10.5	cell wall.cell wall proteins	2	0.572
10.5.1	cell wall.cell wall proteins.AGPs	2	0.572
30.3	signalling.calcium	3	0.615
16.2	secondary metabolism.phenylpropanoids	3	0.622
13.1	amino acid metabolism.synthesis	2	0.622
13.1.2	amino acid metabolism.synthesis.glutamate family	2	0.622
13.1.2.3	amino acid metabolism.synthesis.glutamate family.arginine	2	0.622
13.1.2.3.22	amino acid.synthesis.glutamate family.arginine.arginosuccinate synthase	2	0.622
30.5	signalling.G-proteins	1	0.622
16.1	secondary metabolism.isoprenoids	1	0.622
16.1.5	secondary metabolism.isoprenoids.terpenoids	1	0.622
16.7	secondary metabolism.wax	1	0.646
11.1	lipid metabolism.FA synthesis and FA elongation	2	0.668
11.1.8	lipid metabolism.FA synthesis and FA elongation.acyl coa ligase	2	0.668
15.2	metal handling.binding, chelation and storage	1	0.668
16.8.3	secondary metabolism.flavonoids.dihydroflavonols	3	0.668
11.10	lipid metabolism.glycolipid synthesis	1	0.668

11.10.1	lipid metabolism.glycolipid synthesis.MGDG synthase	1	0.668
33.99	development.unspecified	4	0.721
20.2.99	stress.abiotic.unspecified	2	0.721
10.8	cell wall.pectin*esterases	1	0.721
10.8.2	cell wall.pectin*esterases.acetyl esterase	1	0.721
30.2.24	signalling.receptor kinases.S-locus glycoprotein like	1	0.728
5.10	fermentation.aldehyde dehydrogenase	1	0.782
27.3.6	RNA.regulation of transcription.bHLH,Basic Helix-Loop-Helix family	2	0.786
27.3.24	RNA.regulation of transcription.MADS box transcription factor family	1	0.786
34.2	transporter.sugars	1	0.818
11.8	lipid metabolism."exotics" (steroids, squalene etc)	1	0.83
26.11	misc.alcohol dehydrogenases	2	0.843
27.3.3	RNA.regulation. AP2/Ethylene-responsive element binding protein family	1	0.843
26.19	misc.plastocyanin-like	3	0.86
34.99	transport.misc	2	0.899
26.4	misc.beta 1,3 glucan hydrolases	1	0.908
10.7	cell wall.modification	8	0.911
26.22	misc.short chain dehydrogenase/reductase (SDR)	1	0.913
16.8.5	secondary metabolism.flavonoids.isoflavonols	1	0.936
10.2	cell wall.cellulose synthesis	4	0.936
10.2.1	cell wall.cellulose synthesis.cellulose synthase	4	0.936
34.7	transport.phosphate	1	0.958
20.1	stress.biotic	2	0.995
30.11	signalling.light	2	0.995
23.1	nucleotide metabolism.synthesis	2	0.995
23.1.1	nucleotide metabolism.synthesis.pyrimidine	2	0.995
23.1.1.10	nucleotide metabolism.synthesis.pyrimidine.CTP synthetase	2	0.995
29.5	protein.degradation	1	0.995
29.5.11	protein.degradation.ubiquitin	1	0.995
29.5.11.4	protein.degradation.ubiquitin.E3	1	0.995
29.5.11.4.2	protein.degradation.ubiquitin.E3.RING	1	0.995

Up-regulated genes

13	amino acid metabolism	7	0.00429
3	minor CHO metabolism	6	0.0182
26	misc	60	0.0658
2	major CHO metabolism	3	0.126
29	protein	14	0.142
5	fermentation	3	0.175
30	signalling	25	0.187
20	stress	33	0.201
17	hormone metabolism	22	0.202
33	development	16	0.222
27	RNA	33	0.232
35	not assigned	307	0.375
31	cell	4	0.394
23	nucleotide metabolism	7	0.559
15	metal handling	1	0.584
21	redox.regulation	2	0.604
10	cell wall	12	0.643

34	transport	17	0.663
6	gluconeogenese/ glyoxylate cycle	5	0.683
16	secondary metabolism	21	0.855
28	DNA	1	0.925
11	lipid metabolism	4	0.951
		603	
13.2	amino acid metabolism.degradation	5	0.00133
3.5	minor CHO metabolism.others	4	0.00317
26.12	misc.peroxidases	16	0.00996
13.2.3	amino acid metabolism.degradation.aspartate family	3	0.0141
13.2.3.5	amino acid metabolism.degradation.aspartate family.lysine	3	0.0141
10.2	cell wall.cellulose synthesis	3	0.0258
10.2.1	cell wall.cellulose synthesis.cellulose synthase	3	0.0258
34.3	transport.amino acids	2	0.0564
2.2	major CHO metabolism.degradation	2	0.0564
2.2.2	major CHO metabolism.degradation.starch	2	0.0564
2.2.2.1	major CHO metabolism.degradation.starch.starch cleavage	2	0.0564
6.2	gluconeogenese/ glyoxylate cycle.malate synthase	2	0.0685
26.4	misc.beta 1,3 glucan hydrolases	3	0.0711
27.3.67	RNA.regulation of transcription.putative transcription regulator	5	0.0731
17.7.1.2	hormone metabolism.jasmonate.synthesis-degradation.lipoxygenase	1	0.0848
17.4	hormone metabolism.cytokinin	1	0.104
17.4.2	hormone metabolism.cytokinin.signal transduction	1	0.104
23.4	nucleotide metabolism.phosphotransfer and pyrophosphatases	1	0.106
23.4.99	nucleotide metabolism.phosphotransfer and pyrophosphatases.misc	1	0.106
27.3.3	RNA.regulation. AP2/Ethylene-responsive element binding protein family	3	0.116
11.1	lipid metabolism.FA synthesis and FA elongation	1	0.117
11.1.10	lipid.FA synthesis and FA elongation.beta ketoacyl CoA synthase	1	0.117
30.1	signalling.in sugar and nutrient physiology	2	0.123
20.2.99	stress.abiotic.unspecified	9	0.129
27.3.6	RNA.regulation of transcription.bHLH,Basic Helix-Loop-Helix family	5	0.131
5.10	fermentation.aldehyde dehydrogenase	1	0.137
33.99	development.unspecified	11	0.142
13.2.4	amino acid metabolism.degradation.branched chain group	1	0.145
13.2.4.3	amino acid metabolism.degradation.branched-chain group.valine	1	0.145
13.2.2	amino acid metabolism.degradation.glutamate family	1	0.147
13.2.2.3	amino acid metabolism.degradation.glutamate family.arginine	1	0.147
17.6.3	hormone metabolism.gibberelin.induced-regulated-responsive-activated	2	0.148
17.5.1	hormone metabolism.ethylene.synthesis-degradation	6	0.149
20.2	stress.abiotic	13	0.159
30.3	signalling.calcium	8	0.159
16.10	secondary metabolism.simple phenols	1	0.161
34.13	transport.peptides and oligopeptides	5	0.161
26.13	misc.acid and other phosphatases	3	0.164
17.5	hormone metabolism.ethylene	10	0.165
16.2.1.6	secondary metabolism.phenylpropanoids.lignin biosynthesis.CCoAOMT	1	0.165
29.4	protein.postranslational modification	6	0.181
30.10	signalling.phosphorelay	1	0.188
16.2.1.1	secondary metabolism.phenylpropanoids.lignin biosynthesis.PAL	1	0.188
29.5.3	protein.degradation.cysteine protease	1	0.202

30.2.11	signalling.receptor kinases.leucine rich repeat XI	5	0.212
16.8.4	secondary metabolism.flavonoids.flavonols	3	0.212
17.6.1	hormone metabolism.gibberelin.synthesis-degradation	3	0.212
27.3	RNA.regulation of transcription	33	0.231
31.1	cell.organisation	2	0.235
30.2.2	signalling.receptor kinases.leucine rich repeat II	5	0.258
30.2.3	signalling.receptor kinases.leucine rich repeat III	5	0.258
34.6	transport.sulphate	1	0.259
29.5.11	protein.degradation.ubiquitin	3	0.271
29.5.11.4	protein.degradation.ubiquitin.E3	3	0.271
29.5.11.4.2	protein.degradation.ubiquitin.E3.RING	3	0.271
26.16	misc.myrosinases-lectin-jacalin	1	0.271
5.3	fermentation.ADH	1	0.278
11.8	lipid metabolism."exotics" (steroids, squalene etc)	1	0.281
17.7.1.5	hormone.jasmonate.synthesis-degradation.12-Oxo-PDA-reductase	3	0.309
27.3.37	RNA.regulation of transcription.AS2,lateral organ ooundaries gene family	2	0.324
29.5.1	protein.degradation.subtilases	1	0.328
6.4	gluconeogenese/ glyoxylate cycle.PEPCK	3	0.335
26.21	misc.lipid transfer protein (LTP) family protein	6	0.348
21.1	redox.thioredoxin	1	0.348
16.2.1	secondary metabolism.phenylpropanoids.lignin biosynthesis	4	0.353
26.24	misc.GCN5-related N-acetyltransferase	2	0.353
26.3	misc.gluco-, galacto- and mannosidases	5	0.363
35.2	not assigned.unknown	307	0.375
16.8.3	secondary metabolism.flavonoids.dihydroflavonols	1	0.385
20.2.4	stress.abiotic.touch/wounding	1	0.425
29.5	protein.degradation	5	0.471
27.3.5	RNA.regulation of transcription.ARR	6	0.488
27.3.25	RNA.regulation of transcription.MYB domain transcription factor family	3	0.496
16.8.2	secondary metabolism.flavonoids.chalcones	5	0.515
26.2	misc.UDP glucosyl and glucoronyl transferases	4	0.539
16.2.1.9	secondary metabolism.phenylpropanoids.lignin biosynthesis.COMT	2	0.547
20.1.7	stress.biotic.PR-proteins	6	0.552
26.9	misc.glutathione S transferases	8	0.559
34.7	transport.phosphate	1	0.56
15.2	metal handling.binding, chelation and storage	1	0.584
27.3.32	RNA.regulation of transcription.WRKY domain transcription factor family	4	0.585
27.3.12	RNA.regulation of transcription.C3H zinc finger family	1	0.604
16.8.5	secondary metabolism.flavonoids.isoflavonols	4	0.628
20.1	stress.biotic	20	0.63
20.1.1	stress.biotic.respiratory burst	2	0.635
27.3.7	RNA.regulation of transcription.C2C2(Zn)CO-like, constans-like ZF family	2	0.635
10.7	cell wall.modification	4	0.636
10.6	cell wall.degradation	3	0.639
10.6.3	cell wall.degradation.pectate lyases and polygalacturonases	3	0.639
26.1	misc.misc2	1	0.649
11.9	lipid metabolism.lipid degradation	2	0.665
11.9.4	lipid metabolism.lipid degradation.beta-oxidation	2	0.665
11.9.4.5	lipid metabolism.lipid degradation.beta-oxidation.acyl-CoA thioesterase	2	0.665
16.2	secondary metabolism.phenylpropanoids	7	0.677

17.5.3	hormone metabolism.ethylene.induced-regulated-responsive-activated	2	0.695
10.8	cell wall.pectin*esterases	2	0.716
10.8.1	cell wall.pectin*esterases.PME	2	0.716
29.2	protein.synthesis	3	0.743
26.28	misc.GDSL-motif lipase	1	0.744
16.8	secondary metabolism.flavonoids	13	0.751
29.2.3	protein.synthesis.initiation	2	0.776
30.2	signalling.receptor kinases	14	0.789
13.1	amino acid metabolism.synthesis	2	0.795
13.1.1	amino acid metabolism.synthesis.central amino acid metabolism	2	0.795
13.1.1.3	amino acid metabolism.synthesis.central amino acid metabolism.alanine	2	0.795
13.1.1.3.11	Aa.synthesis.central Aa metabolism.Ala.Ala-glyoxylate aminotransferase	2	0.795
5.2	fermentation.PDC	1	0.824
27.3.23	RNA.regulation of transcription.HSF,Heat-shock transcription factor family	1	0.829
34.99	transport.misc	2	0.83
21.2	redox.ascorbate and glutathione	1	0.838
21.2.1	redox.ascorbate and glutathione.ascorbate	1	0.838
17.5.2	hormone metabolism.ethylene.signal transduction	2	0.843
20.2.1	stress.abiotic.heat	3	0.863
29.2.1	protein.synthesis.ribosomal protein	1	0.871
29.2.1.2	protein.synthesis.ribosomal protein.eukaryotic	1	0.871
29.2.1.2.2	protein.synthesis.ribosomal protein.eukaryotic.60S subunit	1	0.871
29.2.1.2.2.1	protein.synthesis.ribosomal protein.eukaryotic.60S subunit.L10	1	0.871
34.2	transporter.sugars	1	0.875
30.2.99	signalling.receptor kinases.misc	2	0.882
27.3.64	RNA.regulation of transcription.PHOR1	1	0.888
17.2	hormone metabolism.auxin	2	0.898
17.2.3	hormone metabolism.auxin.induced-regulated-responsive-activated	2	0.898
26.10	misc.cytochrome P450	9	0.905
34.16	transport.ABC transporters and multidrug resistance systems	2	0.921
28.1	DNA.synthesis/chromatin structure	1	0.925
3.4	minor CHO metabolism.myo-inositol	2	0.927
3.4.4	minor CHO metabolism.myo-inositol.myo inositol oxygenases	2	0.927
30.2.25	signalling.receptor kinases.wall associated kinase	1	0.939
2.1	major CHO metabolism.synthesis	1	0.958
2.1.1	major CHO metabolism.synthesis.sucrose	1	0.958
2.1.1.3	major CHO metabolism.synthesis.sucrose.FBPase	1	0.958
34.12	transport.metal	3	0.959
17.6	hormone metabolism.gibberelin	5	0.96
26.18	misc.invertase/pectin methylesterase inhibitor family protein	1	0.972
23.2	nucleotide metabolism.degradation	6	0.975
17.7	hormone metabolism.jasmonate	4	0.986
17.7.1	hormone metabolism.jasmonate.synthesis-degradation	4	0.986
31.4	cell.vesicle transport	2	0.986
29.4.1	protein.postranslational modification.kinase	3	0.991
29.4.1.57	protein.postranslation modification.kinase.receptor like cytoplasmic kinase VII	3	0.991
33.1	development.storage proteins	5	0.997
30.2.17	signalling.receptor kinases.DUF 26	2	1
30.2.24	signalling.receptor kinases.S-locus glycoprotein like	2	1

Supplementary Table S3. Fold changes in expression of genes related to BR metabolism and signaling in microarray analysis of *mtbri1* mutants. Red font indicates down-regulation and green font indicates up-regulation.

Gene	Probe Sets	Fold Change	P value
MtBSK1	Mtr.37962.1.S1_at	1.007332463	0.881517439
MtCDG1	Mtr.22348.1.S1_at	0.920531091	0.189735812
MtPP2A	Mtr.12546.1.S1_at	0.912187001	1.44E-05
MtBIN2	Mtr.8603.1.S1_at	1.034859534	0.149000518
MtBSU1	Mtr.38507.1.S1_at	1.156552255	0.038567326
MtBZR1	Mtr.9644.1.S1_at	0.756033991	3.42E-103
	Msa.1199.1.S1_at	0.748788352	7.53E-09
	Mtr.4853.1.S1_at	0.737510568	1.02E-20
MtBZR2	Mtr.12430.1.S1_s_at	1.522488479	9.53E-17
MtCPD	Mtr.41645.1.S1_at	1.293997	1.20E-07
MtDWF4	Mtr.38274.1.S1_at;	5.652685794	0.000269705
MtDET2	Mtr.32396.1.S1_at	0.883043407	0.059397682
MtDET2-1	Mtr.38769.1.S1_at	0.589623117	3.58E-12
MtBAS1	Mtr.1260.1.S1_at	0.364872128	8.09E-05

Supplementary Table S4. E-box and BRRE in the promoter regions of regulated genes in *mtbri1* mutants

Gene ID	# of E-box	Position to ATG	Annotation	# of BRRE	Position to ATG
Medtr3g084150.1	4	2 bp	Auxin responsive SAUR protein	0	
Medtr3g084170.1	5	2 bp	Auxin responsive SAUR protein	0	
Medtr3g084180.1	7	2 bp	Auxin responsive SAUR protein	0	
Medtr3g084190.1	6	150bp	Auxin-induced SAUR protein 6B	0	
Medtr3g084200.1	6	30bp	Auxin responsive SAUR protein	0	
Medtr3g084220.1	4	33bp	Auxin responsive SAUR protein	0	
Medtr3g084230.1	4	200bp	Auxin responsive SAUR protein	0	
Medtr3g084240.1	5	32 bp	Auxin responsive SAUR protein	0	
Medtr3g084250.1	6	140bp	Auxin-induced SAUR protein 6B	0	
Medtr3g110035.1	7	130	Auxin-induced SAUR protein 6B	0	
Medtr4g072240.1	4	195	Auxin-induced SAUR protein 6B	0	
Medtr4g072270.1	7	167 bp	Auxin-induced SAUR protein 6B	0	
Medtr4g072300.1	2	198 bp	Auxin-induced SAUR protein 6B	0	
Medtr4g072310.1	2	499bp	Auxin responsive SAUR protein	0	
Medtr4g072380.1	4	217bp	Auxin-induced SAUR protein 6B	0	
Medtr4g072490.1	8	46 bp	Auxin-induced SAUR protein X10A	0	
Medtr4g072500.1	5	210 bp	Auxin-induced SAUR protein X10A	0	
Medtr4g072530.1	8	210 bp	Auxin-induced SAUR protein 6B	0	
Medtr4g072580.1	5	235	Auxin-induced SAUR protein X10A	0	
Medtr4g072600.1	5	100 bp	Auxin responsive SAUR protein	0	
Medtr4g072620.1	8	204 bp	Auxin-induced SAUR 1	0	
Medtr4g072640.1	6	209 bp	Auxin-induced SAUR protein 6B	0	
Medtr4g072650.1	4	221 bp	Auxin-induced SAUR protein X10A	0	
Medtr4g072660.1	3	126 bp	Auxin responsive SAUR protein	0	
Medtr4g072670.1	9	73 bp	Auxin responsive SAUR protein	0	
Medtr4g072680.1	9	211 bp	Auxin-induced SAUR protein 6B	0	
Medtr4g072720.1	11	79 bp	Auxin-induced SAUR protein X10A	0	
Medtr4g072730.1	11	39 bp	Auxin-induced SAUR protein 6B	0	

Medtr4g072820.1	6	237 bp	Auxin-induced SAUR protein X10A	0	
Medtr4g072830.1	5	222 bp	Auxin-induced SAUR protein X10A	0	
Medtr4g072850.1	2	267 bp	Auxin responsive SAUR protein	0	
Medtr4g072860.1	2	227 bp	Auxin responsive SAUR protein	0	
Medtr4g072890.1	4	169 bp	Auxin-induced SAUR protein 6B	0	
Medtr4g072980.1	4	207 bp	Auxin-induced SAUR protein X10A	0	
Medtr8g461400.1	5	2bp	Auxin responsive SAUR protein	0	
Medtr5g016320.1	0		IAA synthetase GH3.3	0	
Medtr4g133620.1	0		ferric reductase transmembrane	0	
Medtr7g021850.1	3	590 bp	Auxin-induced protein, aldo reductase	0	
Medtr3g095420.1	4	575 bp	Auxin-induced protein 5NG4	0	
Medtr1g014090.1	10	119 bp	Auxin-induced protein 5NG4	0	
Medtr2g007960.1	3	1008 bp	Auxin-induced protein 5NG4	0	
Medtr6g036450.1	4	169 bp	Auxin-induced protein 5NG4	0	
Medtr5g082520.1	2	721 bp	MTCPD	1	184 bp
Medtr5g020020.1	4	115 bp	MtDWF4	0	
Medtr7g066760.1	3	553 bp	MTDET2	0	
Medtr6g043740.1	2	10 bp	MtDET2-l	0	
Medtr4g113650.1	3	298	MtBAS1	0	
Medtr6g015910.1	6	52 bp	Mt BR6ox1	0	
Medtr3g007320.1	2	360 bp	Mt BR6ox2	0	
Medtr3g111680.1	4	420 bp	MtBZR1	0	
Medtr5g019550.1	2	380	MtBZR2	0	
Medtr5g098970.1	0		MtBSK1	0	
Medtr7g083500.1	2	181	MtCDG1	0	
Medtr8g062430.1	3	0 bp	MtPP2A	0	
Medtr2g016490.1	1	256	MtBIN2	0	
Medtr1g089270.1	3	60 bp	MtBSU1	0	
Medtr7g102550.1	4	720	EF transcription factor 12	0	
Medtr7g020980.1	3	361	EF transcription factor	0	
Medtr1g043350.1	1	403	EF transcription factor 2	0	
Medtr1g087920.1	1	550bp	EF transcription factor 2b	0	

Medtr4g127930.1	2	630 bp	AP2-like EF transcription factor	0
Medtr4g100380.1	7	71 bp	EF transcription factor 1A	0
Medtr3g117420.1	1	345 bp	Ethylene-forming-enzyme dioxygenas	0
Medtr4g114570.1	3	89	EF transcription factor WIN1	0
Medtr5g078220.1	7	56 bp	GASA5-like protein	0
Medtr8g076940.1	2	250	Abscisic acid 8prime-hydroxylase	0
Medtr4g106860.1	5	45	Gibberellin regulated protein	0
Medtr2g070870.1	1	996	gibberellin 2-oxidase	0

Supplementary Table S5. Primers used in this study

Primer Name	Sequence (5' to 3')
MtBRI1-F	GAGAGTGTCACATGGCATGTGAG
MtBRI1-F1	ACAACAGCTGTGTCCATCCATCCA
MtBRI1-R	TTCTTGCCACCCTAATGCTTGGT
MtBRI1-R1	ACCATCTCAACGGCGTTGAAACCT
MtBRI1PB1	AAAAAGCAGGCTATGAAACCCTTGTATAGCACAAAC
MtBRI1PB2	AGAAAGCTGGGTCATGCTTGGTTAATTCAGGGACTTC
MtSERK1PB1	AAAAAGCAGGCTTCATGGAGGAGACAAAGTTCTGTGCTT
MtSERK1PB2	AGAAAGCTGGGTCTCTTGGACCAGATAATTCGACTGCA
MtBRI1-RT-F	CTCATCATTCACCCATCTTTCCA
MtBRI1-RT-F1	TGGCGCGTGTACTTCGTTGAC
MtBRI1-RT-R	TATCGAGGTACTGTAGAGATGAA
MtBRI1-RT-R1	ACTCCGGTGGTATTGTTCCTGTGA
MtBR6ox1-F	CAATGATGGCTGTGAAATACCTC
MtBR6ox1-R	GGCCAATCTAGATGTCTCAAAAAT
MtBR6ox2-F	TTAAGCAATTGGGATGACA
MtBR6ox2-R	GCCAGGAAGGTTAATAGGTAGAGA
MtCPD-F	ACCGGGTTTTACTAATGGAGGAAG
MtCPD-R	GGAGTGGTAAGGGAAGGGTAAAAA
MtDET2-F	AACCGCACAATAATCTACCCTCTT
MtDET2-R	ACAACCCAAAAACACCATCCAT
MtDWF4-F	TACTTGGGAAATGGTCTATGTT
MtDWF4-R	TGAGCTGCAAATGTGGTT
MtBAS1-F	TCCGACGGGCTAAAACAGATG
MtBAS1-R	CCAAGGCCAAAAGGTATGAATG
MtBZR1-RF	AGGGGATCAAGGGCAGAAACAC
MtBZR1-RR	AGACGGGCTCGGGAATGAA
MtBZR2-RF	GCCACCCCCTTTTCGCTACC
MtBZR2-RR	GCACCATGGGCAGTTGTTGTCT
MtBRI1-RF	TAACAGGCTTGGTGGTGAGATTCC
MtBRI1-RR	TCCGGTGGTATTGTTCCTGTGAGT
MtABA-D1F	ACACGTGGACCCTTGATGATT
MtABA-D1R	ATTCTTAGGCCGTTTATGTCTCTG
MtABA-U1F	GACCTGGCACCATCAAAAAG
MtABA-U1R	CATCAGAGCCAGCCACAATAC
MtABA-U2F	TTGCAGGCACATCATCTAAAACAC
MtABA-U2R	GATGACGGCGGAGCAACACTGGT
MtAuxin-D1F	GAATGGGTTAGCCTTGTCC
MtAuxin-D1R	ACGCACTATACTTAATTTTCATCC
MtAuxin-D2F	GGAAGTCCCAAAAGGCTATT
MtAuxin-D2R	ATTGTAAGACCACCCATTTGAT
MtAuxin-D3F	CAAAAGGCTATCTCGCAGTCT

MtAuxin-D3R	ATCATATCCAAATTCTTCTTC
MtAuxin-D4F	TGAACCAAGCCGAGGAAC
MtAuxin-D4R	GGAGGAAGACTGTAATGGAAAAG
MtAuxin-D5F	ATGGGTTTCCGCATAGCA
MtAuxin-D5R	GAATCACGAACCGCCTCAT
MtAuxin-D6F	CGTGAGGCGAAAGAACC
MtAuxin-D6R	AAAGCTCCCAATAAATAACATAAT
MtAuxin-D7F	TGGCTGGTGCACCTTACTTG
MtAuxin-D7R	AATCACCATCCTTGTCTTCATA
MtAuxin-U1F	GTTTTGGGTGGGATTGTGC
MtAuxin-U1R	CATTGCGGTGGTTTGGTG
MtAuxin-U2F	GTGACAGAACCGGAAAGGAAC
MtAuxin-U2R	AAAGTACGCAGTCTAACCACAATC
MtCK-U1F	CAAAAACTCTAGAGGCAAAACAG
MtCK-U1R	GGAATGGCTAAATAGTAAAGAACC
MtEth-U1F	AGAGGCTAAAGAGAAAGAGGAGTA
MtEth-U1R	ATTTGTGGGTTATTATGATTGGTA
MtEth-U2F	GGGGTTGGCACACTCGTT
MtEth-U2R	GCACTAAAAGCAGAAGCCACACT
MtEth-D1F	TGCTGAAATTTTAAACCACAAC
MtEth-D1R	TACCCGGCCTTATAGAAAATGAA
MtGA-D1F	ACCCCTTTTCTCCCATTCTTCTAA
MtGA-D1R	CTAATCACACCCCCACCAAAACTT
MtGA-U1F	GTGCGAGACGGAAACAGAAG
MtGA-U1R	AAGCTCCACAAAACTCCCTATTAC
MtGA-U2F	GTCAATACAAATAAGGGGATACAA
MtGA-U2R	AGCAAAAGGGGAATAAACACTA
Tnt1-Fw	ACAGTGCTACCTCCTCTGGATG
Tnt1-F1	TCCTTGTTGGATTGGTAGCCAACTTTGTTG
Tnt1-Re	CAGTGAACGAGCAGAACCTGTG
Tnt1-R1	TGTAGCACCGAGATACGGTAATTAACAAGA
MtACT2-F	TCAATGTGCCTGCCATGTATGT
MtACT2-R	ACTCACACCGTCACCAGAATCC

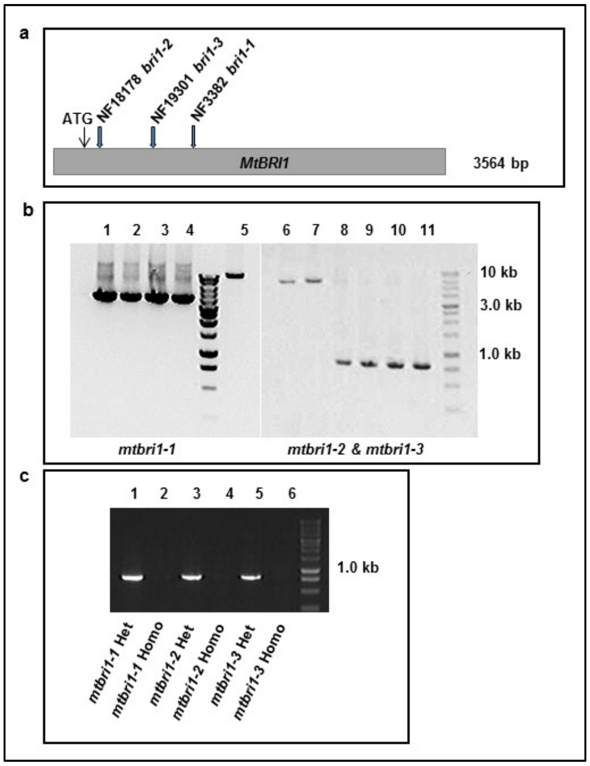

Supplementary Figure S1. Identification of *mtbri1* mutants. (a) Schematic *MtBRI1* gene showing *Tnt1* insertion sites of *mtbri1-1*, *mtbri1-2* and *mtbri1-3*. (b) Genotyping of three insertion mutant lines. Left panel shows genotypes of *mtbri1-1* with MtBRI-F and MtBRI-R primers. Lanes 1-4 are wild-type or heterozygous plants and lane 5 is a homozygous plant for the *Tnt1* insertion; right panel shows genotypes of *mtbri1-2* and *mtbri1-3* with MtBRI-RT-F and MtBRI-RT-R primers. Lanes 6-7 are homozygous plants and lanes 8-11 are wild-type or heterozygous plants. (c) RT-PCR analysis with MtBRI-RF and MtBRI-RR primer pairs spanning *Tnt1* insertions in *mtbri1-1* (lanes 1-2), *mtbri1-2* (lanes 3-4), and *mtbri1-3* (lanes 5-6). Lanes 1, 3, and 5 are heterozygous (Het); lanes 2, 4, and 6 are homozygous (Homo).

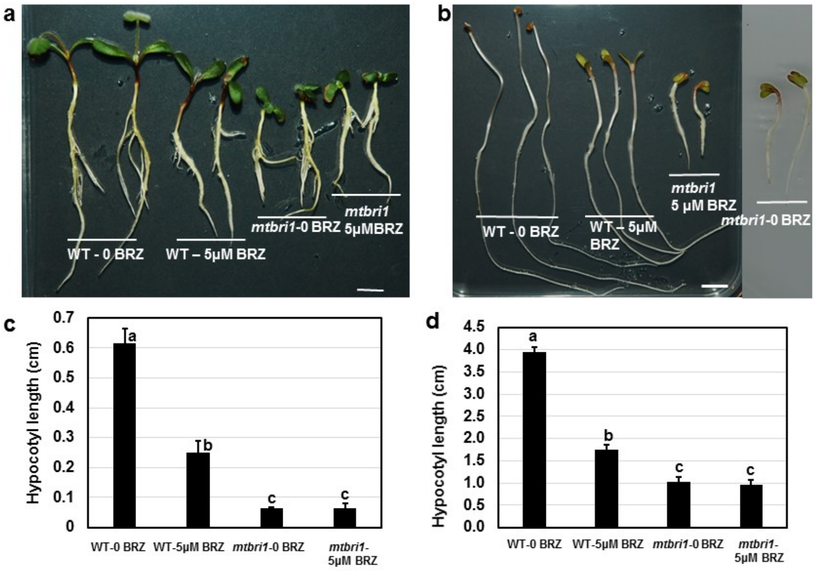

Figure S2. Effects of brassinazole on the elongation of hypocotyls of wild-type and *mtbri1* seedlings under light and dark conditions. (a) Germinated seeds were grown on ½ MS media supplemented with or without 5 µM BRZ for 5 days in light; (b) *mtbri1-1* and wildtype seedlings were grown on ½ MS media supplemented with or without 5 µM BRZ for 5 days in the dark. (c) Measurement of hypocotyl length of seedlings in (a). Error bars represent the standard error of the means ($n \geq 20$). Means with different letters are significantly different ($P < 0.005$; Tukey's test). (d) Measurement of hypocotyl length of seedlings in (b). Error bars represent the standard error of the means ($n \geq 15$). Means with different letters are significantly different ($P < 0.005$; Tukey's test). Scale bars: 10 mm.

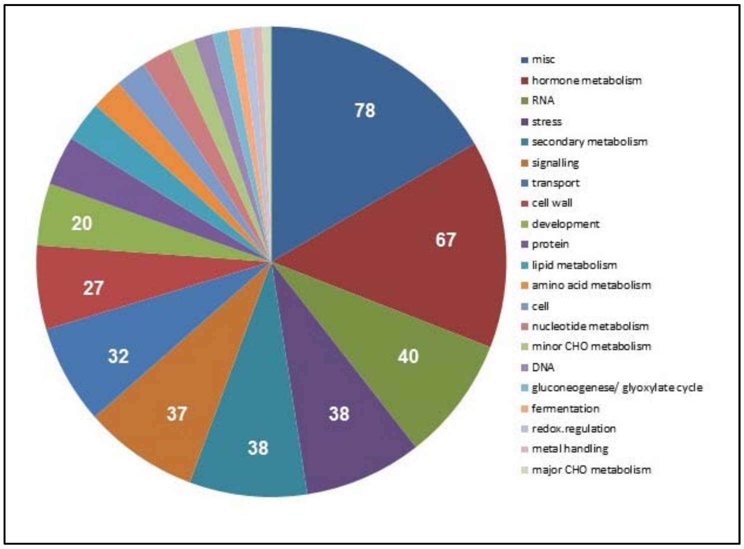

Supplementary Figure S3. Overview of functional classification of the 903 differentially expressed genes (fold change ≥2 for up-regulated genes or ≤ 0.5 for down-regulated genes, P≤ 0.05) from the microarray analysis by MapMan 3.5. 446 of the genes are classified as unknown function and are not displayed.

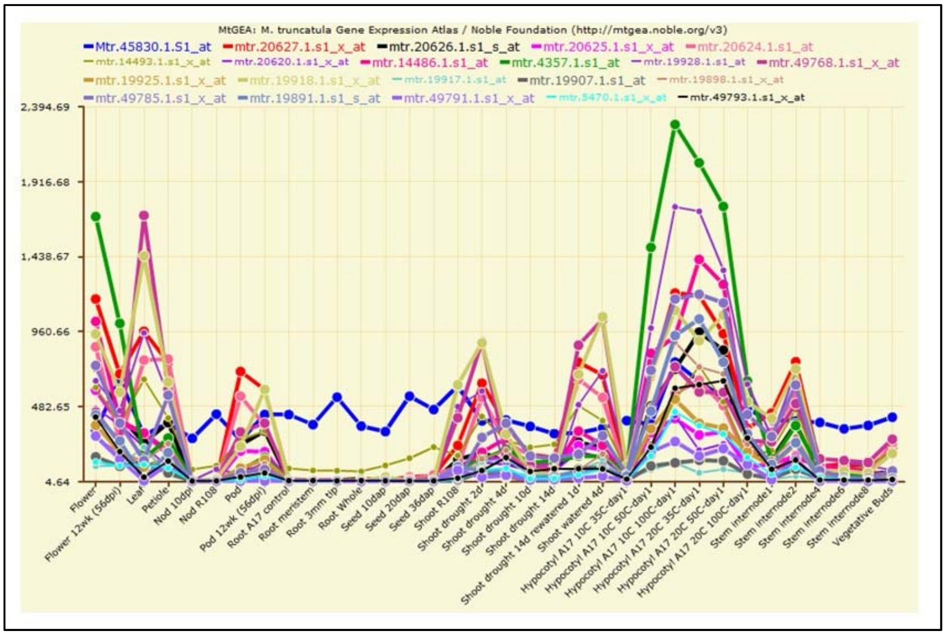

Supplementary Figure S4. *SAUR* gene expression patterns from the Medicago Gene Expression Atlas. Twenty one probe sets corresponding to 21 SAUR genes exhibit similar expression patterns in different tissues and in response to drought stress.

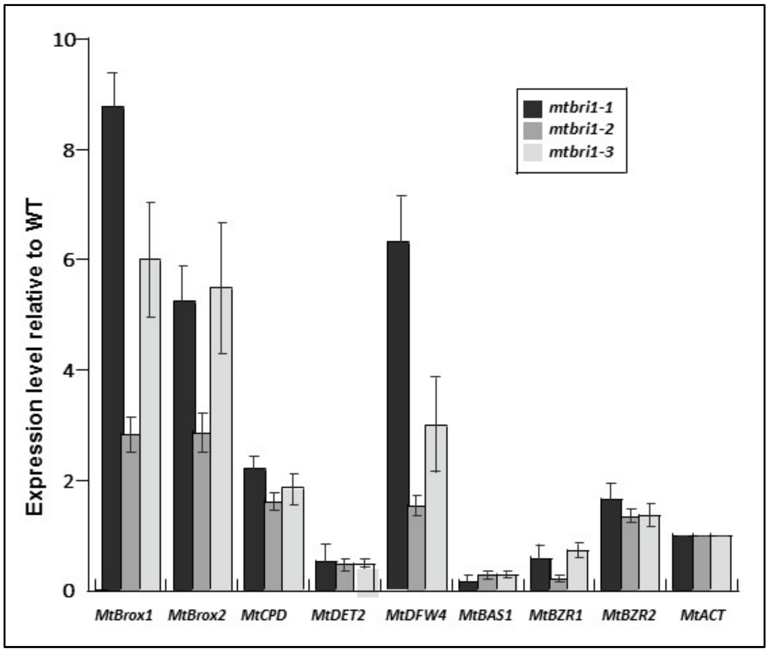

Supplementary Figure S5. qRT-PCR expression analysis of genes in BR metabolism and BR signaling pathway in *mtbri1* mutants. *MtACT* was used as the internal control. The values in Y axis show the expression levels of each gene in *mtbri1* mutants relative to the levels in wild type.

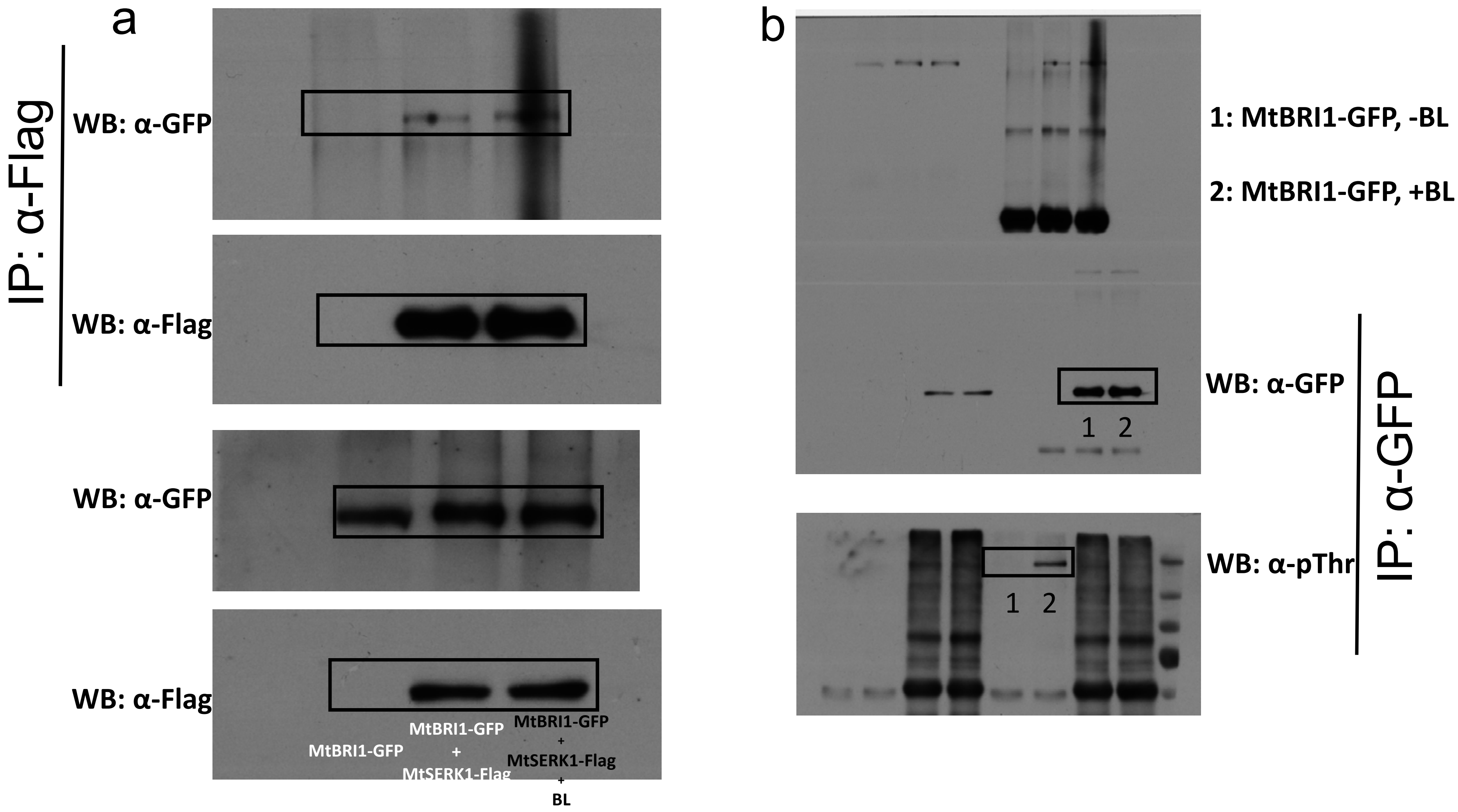

Supplementary Figure S6. Full-length western blots from Figure 5. Lanes used in Figure 5 are outlined with black rectangles.
